# Arylhydrazines: Convenient Homogeneous Reductants for Scalable Cross‐Coupling

**DOI:** 10.1002/anie.9252206

**Published:** 2026-02-06

**Authors:** Nils Kurig, David A. Cagan, Kaid C. Harper, Yu Kawamata, Donna G. Blackmond, Phil S. Baran

**Affiliations:** ^1^ Department of Chemistry Scripps Research La Jolla CA USA; ^2^ Abbvie Process Research and Development North Chicago Illinois USA

**Keywords:** cross‐coupling, Ni‐catalysis, reaction calorimetry, reaction mechanisms, scale‐up

## Abstract

Reductive cross‐couplings have emerged as a powerful strategy for forging C–C bonds directly from electrophiles, circumventing the need for preformed organometallic reagents, yet they often suffer from limitations associated with heterogeneous reductants like Zn (e.g., poor reproducibility and scalability) or costly homogeneous alternatives such as TDAE. Inspired by prior explorations of hydrazide chemistry, we disclose arylhydrazines as inexpensive, readily available homogeneous sacrificial reductants that enable Ni‐catalyzed sp^2^‐sp^3^ cross‐coupling of aryl halides with secondary alkyl iodides under mild, operationally simple conditions using a Ni^II^ precursor, bipyridine ligand, and hindered amine base. Optimization, substrate scope studies, and direct comparisons reveal superior yields and selectivity relative to Zn‐based methods, particularly for heterocyclic and electron‐rich partners, while calorimetry‐guided safety assessments and decagram‐scale demonstrations highlight enhanced thermal control, reproducibility, and practicality. Mechanistic investigations via UV–vis spectroscopy, ^19^F NMR, and reaction calorimetry support a pathway involving hydrazine‐mediated Ni^II^ reduction to initiate a Ni^I^/Ni^III^ cycle, with benign byproducts (N_2_ and arene), positioning arylhydrazines as versatile reagents for executing reductive coupling on scale.

## Graphical abstract

Reductive cross‐couplings enable efficient C–C bond formation but are limited by the heterogeneity of Zn or costly reagents like TDAE. We report arylhydrazines as inexpensive, convenient reductants for Ni‐catalyzed sp^2^–sp^3^ coupling of aryl halides and secondary alkyl iodides. Mechanistic studies support hydrazine‐mediated Ni^II^ reduction for a Ni^I^/Ni^III^ cycle, delivering improved yields, reproducibility, and scalability over Zn‐based methods.

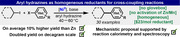

## 1 Introduction

**FIGURE 1:**
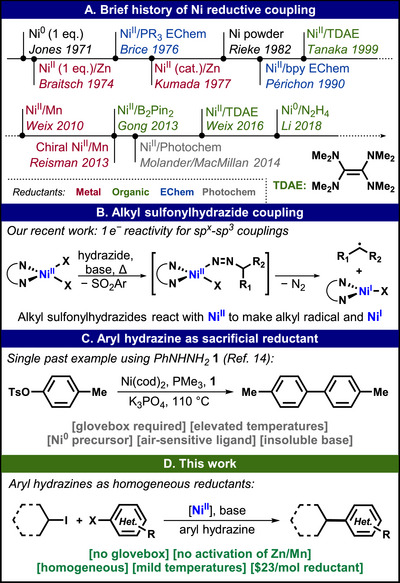
(A) History of Ni reductive coupling. (B) General scheme of the alkyl sulfonylhydrazide coupling. (C) Sole literature precedent for **1** as a sacrificial reductant. (D) This work: aryl hydrazines as sacrificial reductants for benign sp^2^–sp^3^ cross‐coupling.

Reductive cross‐couplings (cross‐electrophile or XEC) have grown in popularity over the past several decades as a way to forge C–C bonds in a more direct fashion without traversing through organometallic intermediates based on Zn, Mg, B, or Sn, and a plethora of methods based on transition‐metal catalysis have been investigated. In particular, Ni‐catalysis, including thermal and electro‐/photochemical redox‐manipulation, has emerged as a promising alternative (Figure 1A). Ni^0^ was first employed to dimerize aryl halides in 1971 [1], immediately followed by studies to form Ni° from Ni^II^ with Zn dust [2] and for in situ use toward sp^2^–sp^2^ coupling [3]. These precedents demonstrated that oxidizable materials such as Zn and Mn can be used to provide the necessary electrons to initiate catalysis of bond formation. Since then, the use of such sacrificial reductants or alternative electron sources has been explored in detail. Electrochemical Ni‐catalysis for C–C bond formation entered the arena in 1976 [4], even before Kumada discovered the first method using Zn to make such reactions catalytic in Ni [5]. In 1982, Rieke presented the coupling of aryl and benzyl halides as part of a series of highly activated materials for organometallic chemistry [6]. The electrochemical toolbox for Ni‐catalyzed coupling was drastically expanded by Périchon starting in 1990 [7].

Ten years later, the first study using a homogenous, molecular reducing agent, tetrakis(dimethylamino)ethylene (TDAE), was published by Tanaka [8]. TDAE became one of the most investigated and derivatized molecules in this field, largely due to the efforts of Weix [9, 10]. Still, TDAE possesses restrictions with respect to availability and handling that pose challenges for its use on scale. An approach to overcome these limitations by derivatizing the amines leads to more stable reagents but increases the price to several hundred dollars per gram [11]. Other homogenous reducing agents for Ni‐catalysis include B_2_Pin_2_ [12], SiMe_4_‐DHP [13], and N_2_H_4_ [14], but literature examples are scarce. Weix's in‐depth studies on TDAE also explored Mn^0^ as a complementary agent to Zn [9]. Reisman employed Mn^0^ together with a Ni^II^/(bis)oxazoline catalyst to achieve stereoselective cross‐coupling [15]. In 2014, photochemical methods using dual‐metal‐catalysis with Ir and Ni were introduced [16, 17]. Most recently, our lab enabled redox‐neutral Ni‐catalyzed cross‐coupling using alkyl sulfonylhydrazides (Figure 1B) [18, 19, 20]. Those studies demonstrated how Ni^I^ can be formed from a Ni^II^ precursor via an electron from diazene decomposition, thereby eliminating exogenous reducing agents. In parallel to this study, an electrochemical method was presented for Ni‐catalyzed reductive cross‐coupling that uses water as a sacrificial reductant in a PEM‐electrolyzer type cell [21]. While being a valuable addition to the toolbox of organic synthesis, [22] electrochemical methods remain challenging to implement, especially using divided cells.

Inspired by the above findings with alkyl sulfonylhydrazides, it was hypothesized that aryl hydrazines themselves might function as sacrificial reductants. Li and coworkers reported the first examples of C–C coupling reactions using N_2_H_4_ and a catalyst generated in situ from Ni^0^(COD)_2_ and PMe_3_ (Figure 1C) [14]. In that study, the scope of aryl triflate and aryl bromide homocoupling was studied, as well as some examples of cross‐coupling with alkyl halides. The authors suggest that using N_2_H_4_ as a reductant together with an insoluble base has practical advantages over other commonly utilized electron sources because the byproducts of N_2_H_4_ oxidation are gaseous N_2_ and H_2_. However, the requirements of a glovebox, unstable low valent Ni, and air‐sensitive ligand (PMe_3_) detract from the practicality of the technique. A lone example in that report of using phenylhydrazine (**1**) as a reducing agent supported our interest in examining it as a clean sacrificial reductant, potentially releasing N_2_ and benzene.

A reductive cross‐coupling method based on a Ni^II^ precursor and **1** would have several advantages over existing methods. From a cost perspective, the price for one mol of **1** ($23 based on 500 g highest purity 97%) and Zn ($24 based on 500 g ACS reagent grade, ≥99.8%, granular, 20–30 mesh) are similar, whereas B_2_Pin_2_ ($670 based on 500 g highest purity 99%) and TDAE ($6170 based on 50 g largest quantity and highest purity 85%) are orders of magnitude more expensive. Since homogeneous reactions are generally more scalable, a benign method based on a Ni^II^ precursor and **1** was pursued.

Disclosed herein are optimized conditions for achieving the reductive cross‐coupling of alkyl and aryl halides using **1** (Figure 1D), its direct comparison with heterogeneous Zn, its scalability/safety profile, and an initial mechanistic exploration using calorimetry and orthogonal spectroscopic methods.

## 2 Results and Discussion

### 2.1 Optimization

**FIGURE 2:**
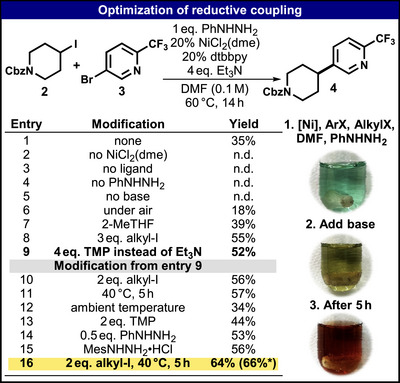
Optimization of reductive coupling using **1** as a sacrificial reductant. *Isolated yield, all others determined by calibrated HPLC using acetanilide as internal UV‐standard. Additional control experiments are given in Table S9, Supporting Information.

Optimization studies were conducted with iodopiperidine **2** and bromopyridine **3** as outlined in Figure 2. Initial conditions were based on the sulfonylhydrazide coupling [18] featuring a Ni^II^ precursor NiCl_2_(dme), 4,4′‐di‐tert‐butyl‐2,2′‐dipyridyl (dtbbpy), phenylhydrazine **1**, and Et_3_N. The initial reaction (entry 1), which was conducted over a period of 16 h at 60°C, resulted in the formation of 35% of the coupling product **4**. Control experiments (entries 2 – 5) demonstrated that each of the reactants was necessary to achieve the desired coupling reaction. Conducting the reaction under air (entry 6) reduced the yield dramatically. Other sacrificial hydrazines and hydrazides initially tested were not advantageous over **1** (see Table S1, Supporting Information). Similarly, extensive screening of ligands, catalyst precursors, temperatures, and base loading showed no improvement (see Tables S2–S5, Supporting Information). Ultimately, a catalyst loading of 20% struck the best balance between yield and turnover (see Table S6, Supporting Information). Solvent screening (see Table S7, Supporting Information) revealed that the reaction could be conducted successfully in polar aprotic, process‐friendly solvents such as 2‐methyl tetrahydrofuran (entry 7) as well as in DMF. As competitive coupling of **2** with a phenyl group (derived from **1**, *vide infra*) and elimination of iodide to the alkene were the most prominent side products, increasing the equivalents of alkyl iodide (entry 8) led to a significant improvement in yield to 55% (see Table S8, Supporting Information). Alternatively, a similar yield enhancement was also achieved by replacing Et_3_N with tetramethyl piperidine (TMP, entry 9), which formed the basis for further optimization (see Tables S10–S18, Supporting Information). Increasing the equivalents of **2** leads to a small increase in yield (entry 10). Decreasing the temperature to 40 °C yielded 57% of **4** after 5 h (entry 11), whereas leaving the reaction at ambient temperature led to reduced yield (entry 12). A similar effect was seen when lowering the base loading to 2 equivalents (entry 13). Interestingly, the equivalents of **1** could be lowered to 0.5 eq. yielding 50% product (entry 14). Sterically hindered hydrazines such as mesityl hydrazine (entry 15) were tested, yielding similar results. This reducing agent proved useful in the coupling of more electron‐rich aryl halides as described in the reaction scope (Table 1). Ultimately, the highest isolated yield of **4** (66%) could be achieved using two equivalents of **2** and lowering the reaction temperature to 40 °C, which led to full conversion after 5 h (entry 16).

**TABLE 1:** Scope of the reductive cross‐coupling comparing reactions with **1** and Zn. Additional examples using mesityl hydrazine are highlighted in red. *Isolated yield, all others determined by ^1^H NMR analysis using dibromomethane as internal standard. ^A^alkyl: Aryl  =  1:2. ^B^alkyl: Aryl  =  1:1. ^C^alkyl: Aryl  =  1:1.5. n.d.  =  not detected.

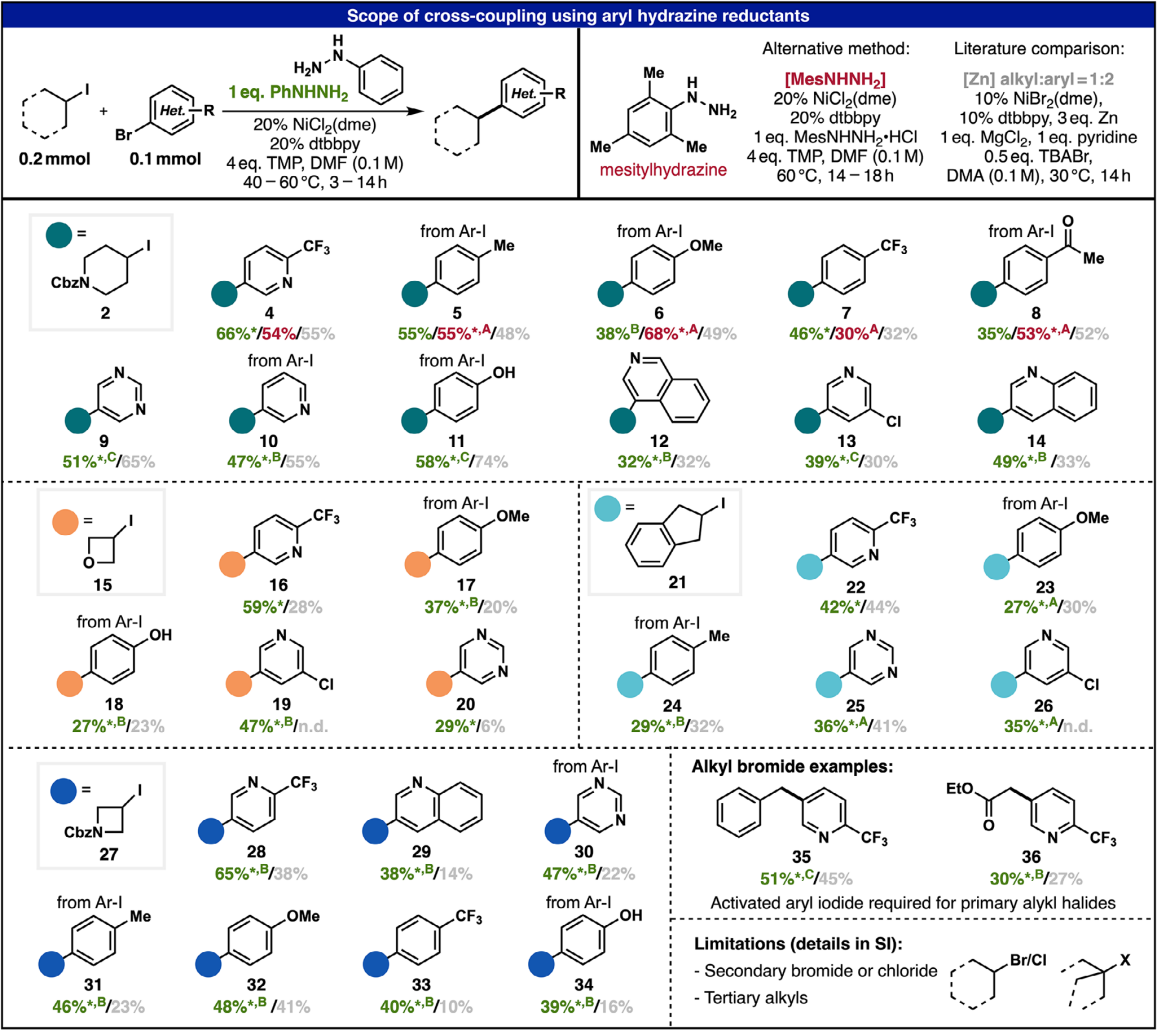

### 2.2 Safety Assessment and Scale‐Up With Comparison to Zinc

**FIGURE 3:**
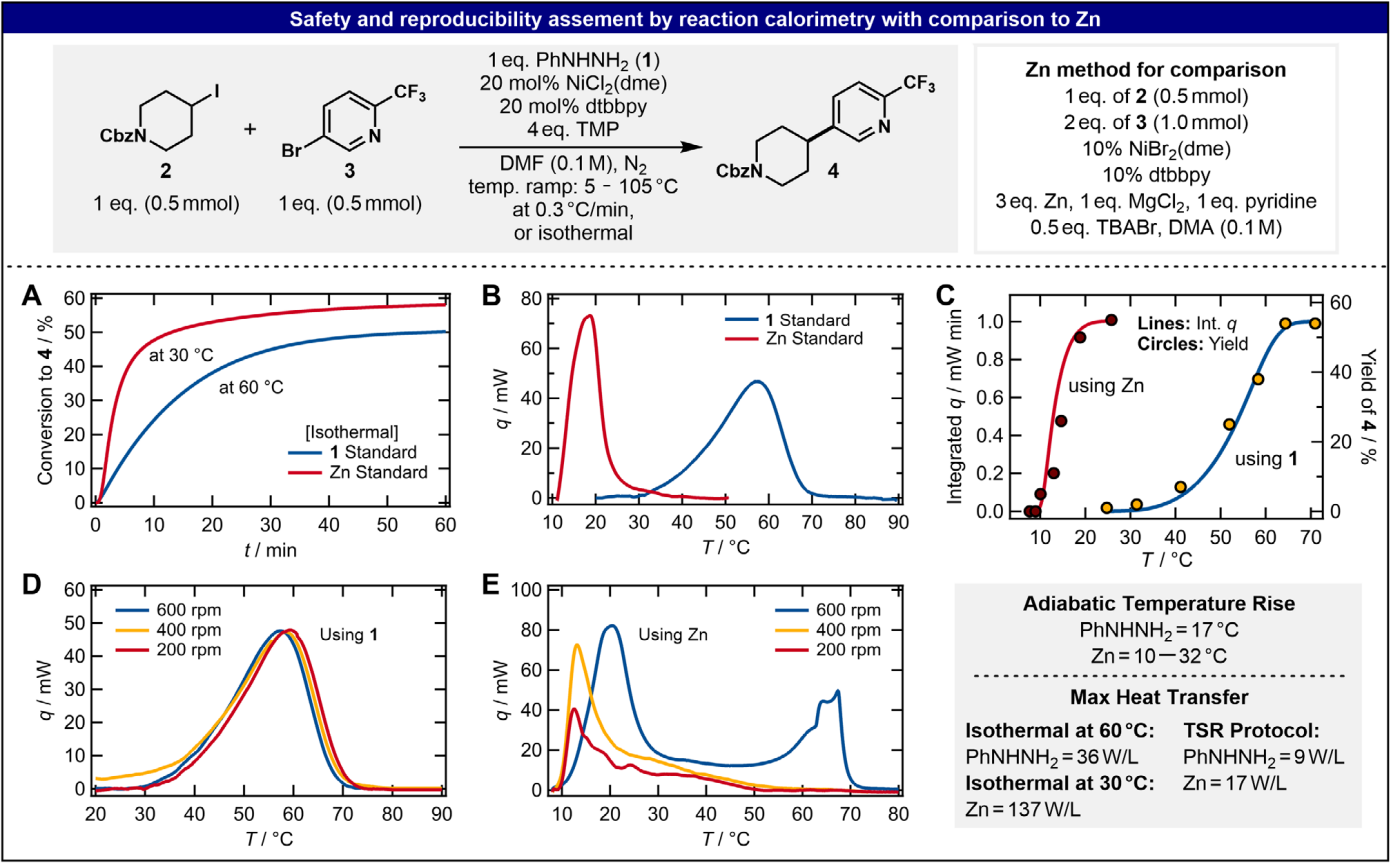
Calorimetry conditions using **1** or Zn as sacrificial reductant (top). Calorimetry data for isothermal (A) and TSR (B) comparison of reactions using **1** or Zn. Sampling experiment confirming product formation is represented by the TSR data (C). Zn and **1** stir rate comparison (D and E). Comparison of key values from the safety assessment is listed. Additional control experiments are given in Figure S11, Supporting Information.

Following our recent investigation, [19] reaction calorimetry (under both isothermal and temperature scanning protocols) was performed on the model system described in Figure 3 (top) to gain further insight into the comparison of the developed hydrazine‐based method with the canonical Zn system (see Tables S19, S20, Supporting Information). Reaction calorimetry monitors the chemical transformation by measuring the system's heat flow (*q*), an observable that is proportional to the rate of the reaction [23, 24]. Temperature scanning reaction (TSR) calorimetry further allows for the simultaneous identification of kinetic and thermodynamic information, [19], [25, 26] providing insight into the reaction mechanism (*vide infra*). Isothermal calorimetric measurements indicated that the coupling of **2** and **3** is nearly completed in 1 h at 60 °C when using **1** as a reductant (55% final yield of product **4**). Under literature conditions at 30 °C, [27] Zn gives similar coupling reactivity with conversion to product reaching a plateau after ca. 20 min and giving a final yield of 58% (Figure 3A). Consistent with this finding, the onset temperature of the Zn system is < 10 °C, while reactions using **1** as a reductant initialize around 35 °C. Importantly, the measured heat flow under the TSR calorimetric protocol was experimentally validated to track the formation of **4** (Figure 3C), making it well‐suited for analysis of the coupling reaction.

In case of the cross‐coupling reaction studied herein, the homogeneous nature and excellent reproducibility of the phenylhydrazine‐based system were apparent from the influence of stir rate on the TSR calorimetry profiles. Using reaction conditions of 1 eq. of **1**, **2**, and **3**, 20 mol% [Ni], and 4 eq. of TMP in 0.1 M DMF, three consecutive reactions in the calorimeter using 200, 400, and 600 rpm stir rate, respectively, led to almost identical heat profiles and yields of 56 ± 2% (Figure 3D). However, in the Zn‐based reaction (1 eq. of **2**, MgCl_2_, and pyridine, 2 eq. of **3**, 10 mol% [Ni], 0.5 eq of TBABr, and 3 eq. of Zn in 0.1 M DMA), the yield dropped by 10% for 200 rpm stir rates vs. 400 and 600 rpm. Moreover, the observed heat profiles with 600 rpm stirring are strikingly different from those with slower stir rates, featuring a second reaction at elevated temperatures (Figure 3E). Further study with reactions containing only one coupling partner under Zn conditions revealed that aryl bromide **3** reacts at a higher temperature than alkyl iodide **2** (see Figure S11B, Supporting Information). Thus, the second onset heat at 50–70 °C is likely due to its stoichiometric excess and depicts a side reaction. However, this second reaction was not observed in an otherwise identical TSR run; poor reproducibility is not uncommon for reductive cross‐coupling using Zn due to inconsistent procedures of Zn‐activation, particle size, and reaction heterogeneity (often requiring 2–3 equivalents of reductant for optimal product yields). Calorimetry studies using the isothermal method indicate that Zn can activate alkyl iodide **2** without a catalyst, marking yet another possible side reaction that must be considered when using the reductant in excess (see Figure S11B, Supporting Information).

Finally, these calorimetric data enabled a safety assessment and comparison between reactions with **1** or Zn (Figure 3), a relevant consideration as certain hydrazines can be considered energetic compounds. Here, an adiabatic temperature rise (ATR, maximum temperature rise if the reaction heat cannot be dissipated from the reactor [28]) of 17 °C with **1** was found, indicative of an exothermic reaction and consistent with the expected nitrogen extrusion process. Given that the exothermic onset temperature of **1** is 239 °C [29], performing the reaction under standard conditions gives an integrated reaction heat per gram of reactant of 274 J/g and an isotherm well below common safety margins [28, 29, 30]. Using the TSR protocol, the maximum heat‐transfer amounts to 9 W/L, even lower than the modest 35 W/L seen when running the reaction under isothermal conditions at the same temperature and thereby presenting temperature ramping as an effective way to minimize heat release without sacrificing product yield. Additionally, TSR measurements could be initiated by the addition of a base, which gives the user an excellent way to control the reaction in an on/off fashion. No heat of reaction is observed in the absence of Ni catalyst, providing a second means of initiating the reaction (see Figure S11A, Supporting Information). Altogether, these thermodynamic metrics are considered safe by accepted process chemistry standards [31]. Comparing these results to the Zn‐based conditions, a decreased ATR of 12 °C was calculated for the case where only one reaction is observed in calorimetry. However, due to the aforementioned reproducibility issues, the ATR rose to 32°C in cases where the side reaction took place at a higher temperature. The maximum heat‐transfer with Zn under the TSR protocol was 17 W/L, or about one‐eighth of that seen under isothermal conditions at 30 °C (137 W/L). Therefore, phenylhydrazine presents a more reproducible alternative to Zn, with similar or improved thermal safety considerations.

**SCHEME 1:**
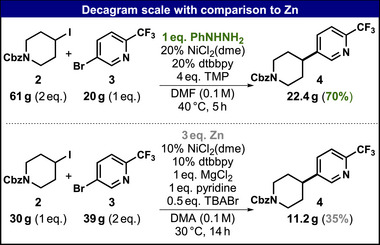
Comparison of **1** and Zn as sacrificial reductants in decagram scale reaction.

Encouraged by these results, large‐scale (decagram) reactions between **2** and **3** were performed with either **1** or Zn as reductant. To mimic the energy/volume ratio of typical large‐scale reactors, scale‐up experiments were conducted in a 1 L Optimax reactor using a 200 rpm stir rate (Scheme 1).

In the phenylhydrazine case, approximately 90 mmol of limiting reagent **3** was converted at 40 °C to yield 22.45 g (70%) of **4**. The Zn comparison experiment was conducted at 30 °C to minimize side reactions and used approximately 90 mmol of **2** as the limiting reagent. Only 11.20 g (35%) of **4** was obtained with Zn, illustrating an advantage of using **1** as a reductant and demonstrating its competency toward large‐scale reactivity. Expectedly, the differences between the hydrazine‐ and Zn‐based systems were magnified on a scale because of the homogeneous reaction conditions.

### 2.3 Scope

With optimized conditions in hand, a substrate scope of secondary alkyl iodides and a wide range of aryl halides was explored (Table 1). Conditions using Zn as a sacrificial reducing agent for comparison studies were taken from the literature [27]. For the majority of substrates studied, hydrazine‐based reducing conditions provided higher yields of product. Indeed, in select cases, the current method stands out, enabling couplings (**19**, **26**) that could not be achieved under Zn‐based conditions. The use of mesityl hydrazine was tested on a small range of aryl halides **4**–**8,** demonstrating that yields could be further improved with alternative hydrazines. Due to the higher cost of mesitylhydrazine (still less expensive than TDAE), the full scope of this reducing agent was not explored.

In the case of **9**, **11**, **13**, **17**–**19**, and **24,** competitive performance was achieved even when using 1:1 stoichiometry of alkyl and aryl donors. The lever of stoichiometry was not pulled for each example, as this is usually decided based on starting material availability. Reactions using oxetane **15** generally worked better with the hydrazine method, especially for heterocyclic examples **16**, **19,** and **20**. Coupling of 2‐iodo‐2,3‐dihydro‐1*H*‐indene **21** generally proved more challenging, giving moderate yields comparable to those of the Zn‐based method due to proto dehalogenation and cross‐coupling with the phenyl motif from **1**. Azetidine donor **27** also performed well with 1:1 stoichiometry and uniformly exceeded the yield observed using Zn as a reducing agent. This hydrazine‐based method is primarily effective for secondary alkyl iodides; primary alkyl and benzylic halides are generally low‐yielding due to competing S_N_ reactions with the hydrazine, and successful couplings require highly activated aryl iodides (5‐iodo‐2‐(trifluoromethyl)pyridine could still give product yields competitive to those with Zn). Secondary alkyl bromides or chlorides and tertiary alkyl halides were not suitable under the present conditions (see Supporting Information).

### 2.4 Reaction Mechanism

**FIGURE 4:**
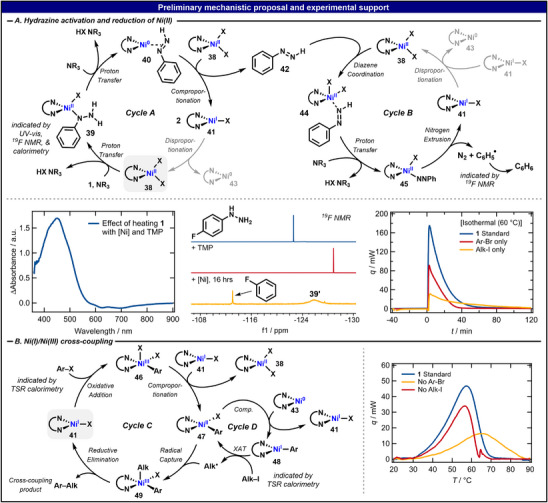
(A) Proposed mechanism for the activation of **1** and reduction of Ni^II^ with supportive difference UV–vis, ^19^F NMR, and isothermal calorimetry data. (B) Ni^I^/Ni^III^ catalytic cycle for the cross‐coupling reaction and TSR calorimetry data to indicate the roles of aryl and alkyl. For the isothermal calorimetry data, the reaction is initiated by adding the halide coupling partner(s) to a pre‐mixed solution of Ni, hydrazine, and base. For the TSR calorimetry, the reaction is initiated by the addition of base to a pre‐mixed solution of Ni, hydrazine, and the halide coupling partner(s).

Whereas a definitive mechanistic picture for this nickel catalysis is outside the scope of this work, a plausible mechanistic proposal was developed by considering the calorimetry results, ^19^F NMR (see Figures S1–S4, Supporting Information), UV–vis spectroscopy (see Figures S5–S10, Supporting Information), and literature precedent (Figure 4). First, the activation of phenylhydrazine by Ni was considered (Cycles A and B). In the presence of base and heat, **1** is deprotonated and replaces a halogen on **38** in a ligand exchange, leading to intermediate **39**. This change in Ni speciation was observed by UV–vis spectroscopy, where the difference spectrum upon heating exhibits absorption features indicative of dtbbpyNi^II^(R)X complexes (Figure 4A, left and Figure S8, Supporting Information), where R is a strongly σ‐donating ligand. [32, 33] Further evidence for this step was given by ^19^F NMR using 4‐fluorophenylhydrazine (*δ*  =  −122.6 ppm) as reductant (Figure 4A, center). Here, deprotonation of the hydrazine is indicated by an upfield shift of the signal by −6.3 ppm, in accordance with previous observations [19]. Addition of Ni to this solution results in the formation of a new species with a peak at −125.9 ppm, the putative Ni‐bound 4‐fluorophenylhydrazine, **39′**.

Deprotonation of **39** is proposed to induce the formation of Ni^0^‐diazene adduct **40** under halogenide elimination and two‐electron reduction of Ni^II^ to Ni^0^. A competing mechanism *via* β‐hydride elimination and formal deprotonative reductive elimination was not ruled out but was considered less likely to occur under the present conditions due to its typically high barrier with Ni [34, 35]. In line with previous reports, Ni^0^ readily reacts with **38** to give two equivalents of Ni^I^ complex **41** via comproportionation [36, 37], releasing diazene **42**. The Ni^I^ complexes may enter downstream cycles or be returned to **38** and **43** by disproportionation [37]. The conversion of **39** to **40** in the absence of aryl or alkyl halide coupling partners is slow. Little to no heat is observed by calorimetry upon mixing **1** with Ni and base at 60 °C (see pre‐time zero data in Figure 4A, right). However, the addition of aryl or alkyl halide immediately initiates a heat of reaction, likely by driving the conversion of **39** forward through consumption of **40** or **41**. Accordingly, the diagnostic UV–vis signal of **39** is quenched upon addition of aryl halide (see Figure S10, Supporting Information).

**FIGURE 5:**
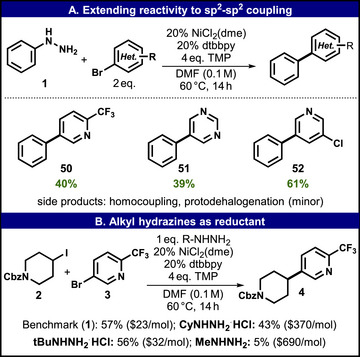
(A) Reactions under a sp^2^‐sp^2^ coupling protocol using **1**. (B) Additional examples using different alkyl hydrazines to replace **1**. Yields determined by ^1^H NMR analysis using dibromomethane as internal standard.

Diazene **42** may decompose on its own [38, 39] or by Ni, the latter of which can be described by Cycle B. Here, **42** engages **38** to form Ni‐adduct **44**. A third equivalent of base converts **44** to Ni^II^‐diazene **45**. Reminiscent of previous reports from our group on Ni^II^ alkyl diazene species [18], radical‐mediated nitrogen extrusion results in Ni^I^‐species **41** and a phenyl radical, which is quenched by hydrogen atom abstraction with solvent or sacrificial starting material to afford benzene. ^19^F NMR supports this process, as 4‐fluorobenzene is observed upon mixing Ni, hydrazine, and base (Figure 4A, center) concomitant with gas evolution. Moreover, we find sp^2^–sp^2^ heterocoupling is possible between the hydrazine and the aryl halide when the alkyl halide coupling partner is removed (*vide infra*, Figure 5A). Finally, a decomposition pathway *via* a phenyl radical intermediate was probed with a TEMPO trap (see Supporting Information) and aligns with the previous results for diazene decomposition. [18], [38, 39] As for Cycle A, **41** may enter downstream cycles or be returned to **38** by comproportionation.

Ni^I^/Ni^III^ cycles for reductive cross‐coupling (Cycles C and D) are well precedented [40]. Here, Ni^I^ **41** undergoes oxidative addition to transient Ni^III^ species **46**. Rapid comproportionation with another equivalent of **41** returns **38** and yields Ni^II^ aryl halide **47** [41, 42]. Conversion of **47** to Ni^I^‐aryl **48** and Ni^I^‐halide **41** in the presence of Ni^0^ has been commonly reported [43, 44, 45]. While either Ni^I^ species may engage the alkyl halide via halogen atom transfer (XAT) pathways [40], TSR calorimetry indicates that **48** is responsible for alkyl radical generation. Reactions performed in the absence of one coupling partner were compared to the standard cross‐coupling conditions (Figure 4B, right). In the absence of an aryl halide, **48** cannot be formed, thereby requiring **41** to activate the alkyl halide. Indeed, a heat of reaction is observed for this process, resulting in predominantly protodehalogenated alkyl side products. This reaction (orange curve in Figure 4B, right) is more sluggish than that performed with aryl halide (red curve), in good agreement with the expected reactivity of **41** toward alkyl and aryl halide coupling partners [46, 47, 48] and would mark a limit to the rate or temperature required for the full reaction. However, the full reaction (blue curve) proceeds more rapidly, at lower temperatures, and appears to be in line with aryl halide activation by **41**. Thus, the more active **48** is invoked to turn over the alkyl halide species by producing an alkyl radical and returning Ni^II^ complex **47**. In turn, **47** captures the alkyl radical to give formal Ni^III^ species **49** [49]. Reductive elimination affords the desired cross‐coupled product and **41**, closing the cycle. Overall, **1** acts as a two‐ or three‐electron reductant (depending on if Cycles A, C‐D, or Cycles A–D are included) and provides entry into Ni^I^/Ni^III^ catalytic cross‐coupling cycles.

Notably, elementary step analysis of the proposed reaction mechanism reveals that the system is a valid turnover catalytic network when Cycles A, C, and D are included (see Supporting Information, Mechanistic Studies section). The inclusion of Cycle B results in the overall conversion of Ni^I^ species **41** into Ni° complex **43** and a decrease in active catalyst concentration over the course of the reaction. Additionally, if the disproportionation of **41** to **43** is slow, the accumulation of **41** likely instead leads to its dimerization and ultimate precipitation from the reaction solution [50, 51, 52]. Therefore, Cycle B competes with the productive cycles. Future work to encourage the self‐decomposition of diazene **42** and reduce the likelihood of entering Cycle B may result in higher catalyst productivity and improved reaction yields.

### 2.5 Additional Reactivity Pathways

Finally, a small set of reactions was studied to provide an outline of potential hydrazine chemistry that is currently underexplored in reductive cross‐coupling. Based on the observation of arylation side product **50** in the cross‐coupling of **2** and **3** using **1** as sacrificial reductant, the sp^2^–sp^2^ coupling of **1** with additional heteroaryl bromides was explored (Figure 5A). The literature features examples of Suzuki‐type reactivity employing Pd to denitrogenatively couple hydrazines with aryl iodides or aryl boronic acids [53, 54, 55]. Additionally, a sole example of using Ni under photochemical conditions to couple hydrazines to aryls has been reported [56]. Despite aryl hydrazines not commonly being used in sp^2^–sp^2^ coupling, robust yields of 40%–60 % for **50**–**52** were achieved using the method developed herein.

While the present study focused on aryl hydrazines, alkyl hydrazines also emerged as a potentially valuable class of sacrificial reductants in this context. Initial reactions using methyl hydrazine were low‐yielding; cyclohexyl hydrazine afforded a moderate yield of **4** (43%), but it is cost‐restrictive. The best result of 56% (comparable to 57% when using **1**) was achieved using tert‐butyl hydrazine, which is still 50% more expensive compared to **1** but promising with respect to avoiding side reaction pathways, as no undesired coupling of tert‐butyl to **2** or **3** was observed.

## 3 Conclusion

In conclusion, this study establishes aryl and alkylhydrazines as a practical and economical class of homogeneous reductants for Ni‐catalyzed reductive cross‐coupling, offering a benign alternative to traditional heterogeneous metals like Zn with advantages in scalability, safety, and reproducibility as evidenced by calorimetry and large‐scale experiments. By enabling efficient sp^2^–sp^3^ bond formation under mild conditions with broad substrate compatibility—including challenging heterocycles and oxetanes—this method streamlines access to medicinally relevant scaffolds while minimizing side reactions and operational complexity. The proposed mechanism, supported by spectroscopic and qualitative kinetic data, underscores the dual role of arylhydrazines as homogenous electron donors and radical precursors, opening avenues for further innovations in catalysis.

## Conflicts of Interest

The authors declare no conflicts of interest.

## Supporting information




**Supporting File 1**: anie71405‐sup‐0001‐SuppMat.pdf.

## Data Availability

The data that support the findings of this study are available from the corresponding author upon reasonable request.
