# Supplementary material for: Arylhydrazines: Convenient Homogeneous Reductants for Scalable Cross‐Coupling
Source: Angew Chem Int Ed Engl. 2026 Feb 6;65(12):e9252206. doi: 10.1002/anie.9252206 (PMC12991040; doi:10.1002/anie.9252206)

Arylhydrazines: Convenient Homogeneous Reductants for Scalable Cross-Coupling

Nils Kurig†1, David A. Cagan†1, Kaid C. Harper[2], Yu Kawamata[1], Donna G. Blackmond[1*], Phil S. Baran[1*]

[1]Department of Chemistry, Scripps Research, 10550 North Torrey Pines Road, La Jolla, CA 92037 (USA)

[2]Abbvie Process Research and Development, North Chicago, IL 60064, USA.

†These authors contributed equally to this work

*Correspondence to: blackmon@scripps.edu, pbaran@scripps.edu

SUPPORTING INFORMATION

Experimental Procedures and Characterization Data

Table of Contents

GENERAL EXPERIMENTAL **5**

EXPERIMENTAL PROCEDURES **5**

STARTING MATERIAL SYNTHESIS A: BENZYL 4-IODOPIPERIDINE-1-CARBOXYLATE 5
STARTING MATERIAL SYNTHESIS B: 2-IODO-2,3-DIHYDRO-1*H*-INDENE 6
GENERAL PROCEDURE A: PHENYLHYDRAZINE 6
GENERAL PROCEDURE B: MESITYLHYDRAZINE 7
GENERAL PROCEDURE C: ZINC 7

INITIAL OPTIMIZATION **8**

TABLE S1: SACRIFICIAL REDUCTANTS I 8
TABLE S2: LIGANDS 8
TABLE S3: PRECATALYST 9
TABLE S4: REACTION TEMPERATURE I 9
TABLE S5: BASE EQUIVALENTS I 9
TABLE S6: CATALYST LOADING I 9
TABLE S7: SOLVENTS I 10
TABLE S8: STARTING MATERIALS 10
TABLE S9: CONTROL EXPERIMENTS 10

TABLE S10: BASE 11
FURTHER OPTIMIZATION 11
TABLE S11: SACRIFICIAL REDUCTANTS II 11
TABLE S12: SOLVENTS II 11
TABLE S13: BASE EQUIVALENTS II 11
TABLE S14: REDUCTANT EQUIVALENTS 12
TABLE S15: REACTION TEMPERATURE II 12
TABLE S16: CATALYST LOADING II 12
TABLE S17: NI TO LIGAND RATIO 12
TABLE S18: SUBSTITUTED PHENYLHYDRAZINES 12
CHARACTERIZATION DATA 13
Compound 4 13
Compound 5 13
Compound 6 14
Compound 7 14
Compound 8 15
Compound 9 15
Compound 10 15
Compound 11 16
Compound 12 16
Compound 13 17
Compound 14 17
Compound 16 18
Compound 17 18
Compound 18 18
Compound 19 19
Compound 20 19
Compound 22 20
Compound 23 20
Compound 24 20
Compound 25 21
Compound 26 21
Compound 28 22
Compound 29 22
Compound 30 22
Compound 31 23
Compound 32 23
Compound 33 24
Compound 34 24
Compound 35 24
Compound 36 25
ENCOUNTERED LIMITATIONS 25
SCALE-UP 26
Phenylhydrazine conditions 26
Zinc conditions 26

MECHANISTIC STUDIES 28
^{19}F NMR studies 28
UV-vis studies 31
Reaction calorimetry 34
Elementary Step Analysis of Proposed Mechanism 39
FURTHER EXPERIMENTS 41
Alkyl iodide activation with Zn 41
TEMPO trap experiments 41
Ni^0 trapping 42
Slow addition experiments 42
FREQUENTLY ASKED QUESTIONS 43
ABBREVIATIONS 45
REFERENCES 46
SPECTRAL DATA 47
^{1}H NMR of Compound 4 (600 MHz, $CDCl_3$): 47
^{13}C NMR of Compound 4 (151 MHz, $CDCl_3$): 47
^{19}F NMR of Compound 4 (376 MHz, $CDCl_3$): 48
^{1}H NMR of Compound 5 (600 MHz, $CDCl_3$): 48
^{13}C NMR of Compound 5 (151 MHz, $CDCl_3$): 49
^{1}H NMR of Compound 6 (600 MHz, $CDCl_3$): 49
^{13}C NMR of Compound 6 (151 MHz, $CDCl_3$): 50
^{1}H NMR of Compound 7 (600 MHz, $CDCl_3$): 50
^{13}C NMR of Compound 7 (151 MHz, $CDCl_3$): 51
^{19}F NMR of Compound 7 (376 MHz, $CDCl_3$): 51
^{1}H NMR of Compound 8 (600 MHz, $CDCl_3$): 52
^{13}C NMR of Compound 8 (151 MHz, $CDCl_3$): 52
^{1}H NMR of Compound 9 (600 MHz, $CDCl_3$): 53
^{13}C NMR of Compound 9 (151 MHz, $CDCl_3$): 53
^{1}H NMR of Compound 10 (600 MHz, $CDCl_3$): 54
^{13}C NMR of Compound 10 (151 MHz, $CDCl_3$): 54
H NMR of Compound 11 (600 MHz, $CDCl_3$): 55
^{13}C NMR of Compound 11 (151 MHz, $CDCl_3$): 55
^{1}H NMR of Compound 12 (600 MHz, $CDCl_3$): 56
^{13}C NMR of Compound 12 (151 MHz, $CDCl_3$): 56
^{1}H NMR of Compound 13 (600 MHz, $CDCl_3$): 57
^{13}C NMR of Compound 13 (151 MHz, $CDCl_3$): 57
^{1}H NMR of Compound 14 (600 MHz, $CDCl_3$): 58
^{13}C NMR of Compound 14 (151 MHz, $CDCl_3$): 58
^{1}H NMR of Compound 16 (600 MHz, $CDCl_3$): 59
^{13}C NMR of Compound 16 (151 MHz, $CDCl_3$): 59
^{19}F NMR of Compound 16 (376 MHz, $CDCl_3$): 60
^{1}H NMR of Compound 17 (600 MHz, $CDCl_3$): 60
^{13}C NMR of Compound 17 (151 MHz, $CDCl_3$): 61
^{1}H NMR of Compound 18 (600 MHz, $CDCl_3$): 61
^{13}C NMR of Compound 18 (151 MHz, $CDCl_3$): 62

^1H NMR of Compound 19 (600 MHz, $CDCl_3$):........ 62
^{13}C NMR of Compound 19 (151 MHz, $CDCl_3$):........ 63
^1H NMR of Compound 20 (600 MHz, $CDCl_3$):........ 63
^{13}C NMR of Compound 20 (151 MHz, $CDCl_3$):........ 64
^1H NMR of Compound 22 (600 MHz, $CDCl_3$):........ 64
^{13}C NMR of Compound 22 (151 MHz, $CDCl_3$):........ 65
^{19}F NMR of Compound 22 (376 MHz, $CDCl_3$):........ 65
^1H NMR of Compound 23 (600 MHz, $CDCl_3$):........ 66
^{13}C NMR of Compound 23 (151 MHz, $CDCl_3$):........ 66
^1H NMR of Compound 24 (600 MHz, $CDCl_3$):........ 67
^{13}C NMR of Compound 24 (151 MHz, $CDCl_3$):........ 67
^1H NMR of Compound 25 (600 MHz, $CDCl_3$):........ 68
^{13}C NMR of Compound 25 (151 MHz, $CDCl_3$):........ 68
^1H NMR of Compound 26 (600 MHz, $CDCl_3$):........ 69
^{13}C NMR of Compound 26 (151 MHz, $CDCl_3$):........ 69
^1H NMR of Compound 28 (600 MHz, $CDCl_3$):........ 70
^{13}C NMR of Compound 28 (151 MHz, $CDCl_3$):........ 70
^{19}F NMR of Compound 28 (376 MHz, $CDCl_3$):........ 71
^1H NMR of Compound 29 (600 MHz, $CDCl_3$):........ 71
^{13}C NMR of Compound 29 (151 MHz, $CDCl_3$):........ 72
^1H NMR of Compound 30 (600 MHz, $CDCl_3$):........ 72
^{13}C NMR of Compound 30 (151 MHz, $CDCl_3$........ 73
^1H NMR of Compound 31 (600 MHz, $CDCl_3$):........ 73
^{13}C NMR of Compound 31 (151 MHz, $CDCl_3$):........ 74
^1H NMR of Compound 32 (600 MHz, $CDCl_3$):........ 74
^{13}C NMR of Compound 32 (151 MHz, $CDCl_3$):........ 75
^1H NMR of Compound 33 (600 MHz, $CDCl_3$):........ 75
^{13}C NMR of Compound 33 (151 MHz, $CDCl_3$):........ 76
^{19}F NMR of Compound 33 (376 MHz, $CDCl_3$):........ 76
^1H NMR of Compound 34 (600 MHz, $CDCl_3$):........ 77
^{13}C NMR of Compound 34 (151 MHz, $CDCl_3$):........ 77
^1H NMR of Compound 35 (600 MHz, $CDCl_3$):........ 78
^{13}C NMR of Compound 35 (151 MHz, $CDCl_3$):........ 78
^{19}F NMR of Compound 35 (376 MHz, $CDCl_3$):........ 79
^1H NMR of Compound 36 (600 MHz, $CDCl_3$):........ 79
^{13}C NMR of Compound 36 (151 MHz, $CDCl_3$):........ 80
^{19}F NMR of Compound 36 (376 MHz, $CDCl_3$):........ 80

General Experimental

Solvents used were ACS grade (99.8%) or higher and reagents were purchased at the highest commercial quality, both were used without further purification, unless otherwise stated. Yields refer to chromatographically and spectroscopically (^{1}H NMR) homogeneous material, unless otherwise stated. Reactions were monitored by thin layer chromatography (TLC) carried out on 0.25 mm E. Merck silica plates (60F-254), using UV light as the visualizing agent and $KMnO_4$ stain and heat as developing agent, unless otherwise stated. Flash silica gel chromatography was performed using E. Merck silica gel (60, particle size 0.043 – 0.063 mm). NMR spectra were recorded on Bruker DRX-600 instruments and were calibrated using residual undeuterated solvent as an internal reference ($CHCl_3$ 7.26 ppm ^{1}H NMR, 77.16 ppm ^{13}C NMR). The following abbreviations were used to explain NMR peak multiplicities: s = singlet, d = doublet, t = triplet, q = quartet, m = multiplet, br = broad. High-resolution mass spectra (HRMS) were recorded on an Agilent Technologies LC/MSD TOF mass spectrometer by electrospray ionization time-of-flight (ESI-TOF) reflection experiments.

Experimental Procedures

Starting Material Synthesis A: Benzyl 4-iodopiperidine-1-carboxylate

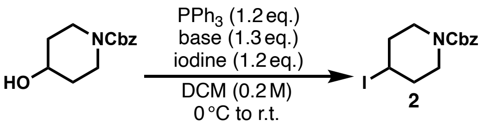

A round bottom flask (500 mL) equipped with a stir bar was charged with the benzyl 4-hydroxypiperidine-1-carboxylate (7.05 g, 30 mmol, 1 eq.), triphenylphosphine (9.44 g, 36 mmol, 1.2 eq.) and imidazole (2.66 g, 39 mmol, 1.3 eq.). After adding DCM (150 mL), the mixture was cooled down to 0 °C. Iodine (9.14 g, 36 mmol, 1.2 eq.) was added in portions, not allowing the temperature to rise above 8 °C. Then, the cooling was removed, and the reaction mixture was stirred overnight to completion. Water (240 mL) was added, and the two phases were separated. The aqueous phase was washed with DCM (3x80 mL). The combined organic phases were washed with $Na_2S_2O_3$ (240 mL) and brine (240 mL), dried over $MgSO_4$ and the solvent was removed and room temperature. Flash column chromatography (hexanes:EtOAc = 3:1) yielded 10.03 g (95%) of the desired commercial product **2** consistent with reported NMR data.[1]

^{1}H NMR (600 MHz, $CDCl_3$): δ 7.39 – 7.30 (m, 5H), 5.13 (s, 2H), 4.46 (ddd, *J* = 11.6, 6.6, 5.0 Hz, 1H), 3.65 (dt, *J* = 13.7, 5.3 Hz, 2H), 3.46 – 3.33 (m, 2H), 2.03 (s, 4H).

^{13}C NMR (151 MHz, $CDCl_3$): δ 155.3, 136.7, 128.7, 128.2, 128.1, 67.4, 44.0, 37.2, 27.3.

Starting Material Synthesis B: 2-iodo-2,3-dihydro-1*H*-indene

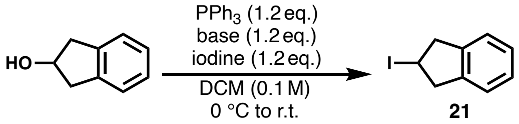

A round bottom flask (100 mL) equipped with a stir bar was charged with the 2,3-dihydro-1*H*-inden-2-ol (0.67 g, 5 mmol, 1 eq.), triphenylphosphine (1.57g, 6 mmol, 1.2 eq.) and imidazole (0.41 g, 6 mmol, 1.2 eq.). After adding DCM (50 mL), the mixture was cooled down to 0 °C. Iodine (1.52 g, 6 mmol, 1.2 eq.) was added in portions, not allowing the temperature to rise above 8 °C. Then, the cooling was removed, and the reaction mixture was stirred overnight to completion. Water (30 mL) was added, and the two phases were separated. The aqueous phase was washed with DCM (3x30 mL). The combined organic phases were washed with $Na_2S_2O_3$ (30 mL) and brine (30 mL), dried over $MgSO_4$ and the solvent was removed and room temperature. Flash column chromatography (0 – 2% EtOAc in hexanes) yielded 1.04 g (85%) of the desired commercial product consistent with reported ^{1}H NMR data.[2,3]

^{1}H NMR (600 MHz, $CDCl_3$): δ 7.23 (ddd, J = 31.3, 5.3, 2.8 Hz, 5H), 4.71 (tt, J = 6.5, 5.0 Hz, 1H), 3.52 – 3.34 (m, 4H).

^{13}C NMR (151 MHz, $CDCl_3$): δ 141.6, 127.1, 124.5, 46.7, 24.0.

General Procedure A: Phenylhydrazine

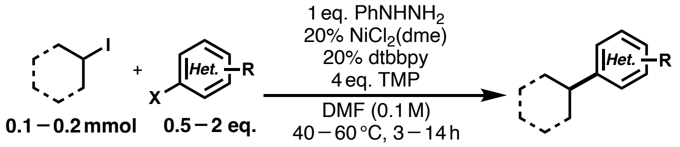

To a long tube, a stirrer bar, the alkyl iodide (0.1 – 0.2 mmol, 1 eq.), the (hetero)aryl halide (0.5 – 2 eq.), $NiCl_2$(dme) (4 mg, 0.02 mmol, 20 mol%), and dtbbpy (5 mg, 0.02 mmol, 20 mol%) were added. The vial was capped with a septa cap, then evacuated and backfilled with Ar three times. Dry DMF (1 mL) was added, followed by $PhNHNH_2$ (11 mg, 10 µL, 0.1 mmol, 1 eq.), then the mixture was stirred at room temperature for a minute to dissolve the solids. Next, tetramethylpiperidine (14 mg, 66 µL, 0.4 mmol, 4.0 eq.) was added. The cap was secured with a layer of labfilm and the tube was placed in a pre-heated oil bath and stirred overnight.

After completion, the reaction solution was diluted with EtOAc (7 mL) and sat. $NaHCO_3$ (14 mL) and the phases were separated. The aqueous phase was extracted with EtOAc (7 mL). Then, combined organic phases were dried over $MgSO_4$, filtered, and the solvent was removed under reduced pressure.

General Procedure B: Mesitylhydrazine

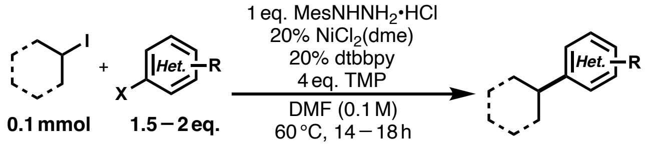

To a long tube, a stirrer bar, the alkyl iodide (0.1 mmol, 1 eq.), the (hetero)aryl halide (1.5 – 2 eq.), $NiCl_2$(dme) (4 mg, 0.02 mmol, 20 mol%), and dtbbpy (5 mg, 0.02 mmol, 20 mol%) were added. The vial was capped with a septa cap, then evacuated and backfilled with Ar three times. Dry DMF (1 mL) was added, followed by $MesNHNH_2$•HCl (19 mg, 0.1 mmol, 1 eq.), then the mixture was stirred at room temperature for a minute to dissolve the solids. Next, tetramethylpiperidine (14 mg, 66 µL, 0.4 mmol, 4.0 eq.) was added. The cap was secured with a layer of labfilm and the tube was placed in a pre-heated oil bath and stirred overnight.

After completion, the reaction solution was diluted with EtOAc (7 mL) and sat. $NaHCO_3$ (14 mL) and the phases were separated. The aqueous phase was extracted with EtOAc (7 mL). Then, combined organic phases were dried over $MgSO_4$, filtered, and the solvent was removed under reduced pressure.

General Procedure C: Zinc

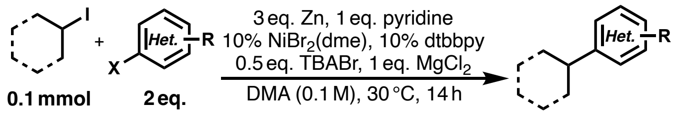

A representative method for Zn-comparison was taken from the literature.[4] To a long tube, a stirrer bar, the alkyl iodide (0.1 mmol, 1.0 eq.), the (hetero)aryl halide (2 eq., 0.2 mmol), $NiBr_2$(dme) (3 mg, 0.01 mmol, 10 mol%), dtbbpy (3 mg, 0.01 mmol, 10 mol%), $MgCl_2$ (10 mg, 0.1 mmol, 1.0 eq.), TBABr (16 mg, 0.05 mmol, 50 mol%), and Zn (20 mg, 0.3 mmol, 3.0 eq.) were added. The vial was capped with a septa cap, then evacuated and backfilled with Ar three times. Note: In case of the optimization substrates, the alkyl iodide is a highly viscous liquid but not volatile on this timescale. Dry DMA (1 mL) was added, followed by dry pyridine (8 mg, 8 µL, 0.1 mmol, 1 eq.). The cap was secured with a layer of labfilm and the tube was placed in a pre-heated oil bath at 30 °C and stirred overnight.

After completion, the reaction solution was diluted with EtOAc (7 mL) and $NaHCO_3$ (14 mL) and the phases were separated. The aqueous phase was extracted with EtOAc (7 mL). Then, combined organic phases were dried over $MgSO_4$, filtered, and the solvent was removed under reduced pressure.

Initial optimization

Table S1: Sacrificial reductants I

entry	reaction conditions	yield
1	$PhNHNH_2$	30%
2	$TsNHNH_2$	8%
3	$PhNHNH(dFArSO_2)$	3%
4	$BocNHNH_2$	n.d.
5	$NH_2NH_2{*}H_2O$	n.d.
6	NH_2NH_2 1M THF	traces
7	$PhNHNH_2{*}HCl$	15%
8	$Ph_2NNH_2{*}HCl$	n.d.
9	TsNHOH	n.d.
10	Me_2NNH_2	n.d.

Table S2: Ligands

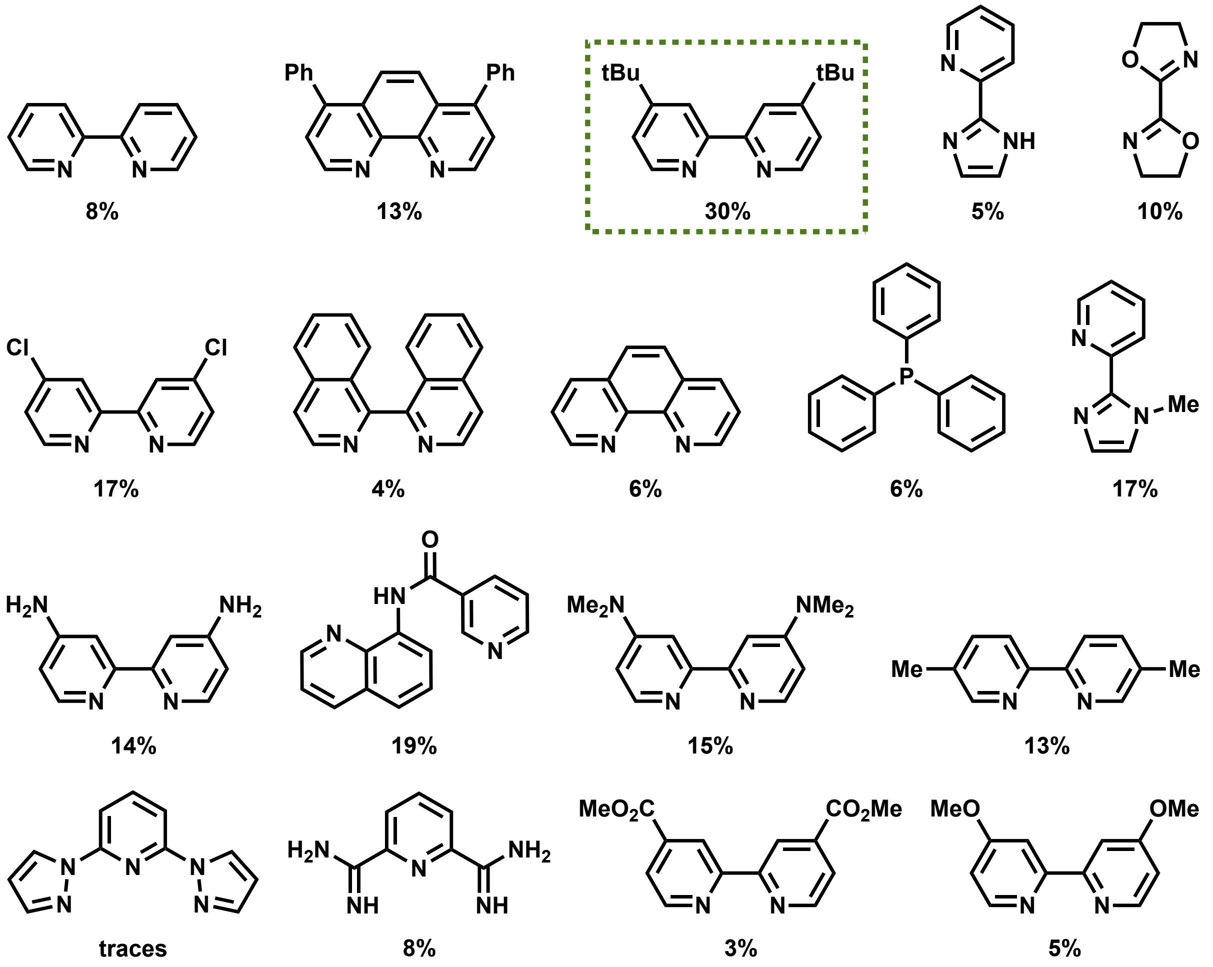

Table S3: Precatalyst

entry	reaction conditions	yield
1	$NiCl_2$(dme)	33%
2	$NiBr_2$(dme)	32%
3	$Ni(NO_3)_2$*$6H_2O$	30%
4	Ni(cod)DQ	30%
5	$NiBARF_2$ (dtbbpy adduct)	30%

Table S4: Reaction temperature I

entry	reaction conditions	yield
1	r.t.	34%
2	30 °C	32%
3	40 °C	35%
4	50 °C	34%
5	60 °C	33%
6	70 °C	33%

Table S5: Base equivalents I

entry	reaction conditions	yield
1	1 eq.	24%
2	2 eq.	25%
3	3 eq.	33%
4	4 eq.	35%
5	5 eq.	27%
6	6 eq.	32%

Table S6: Catalyst loading I

entry	reaction conditions	yield
1	10 mol%	24%
2	20 mol%	33%
3	30 mol%	39%
4	40 mol%	41%
5	50 mol%	47%

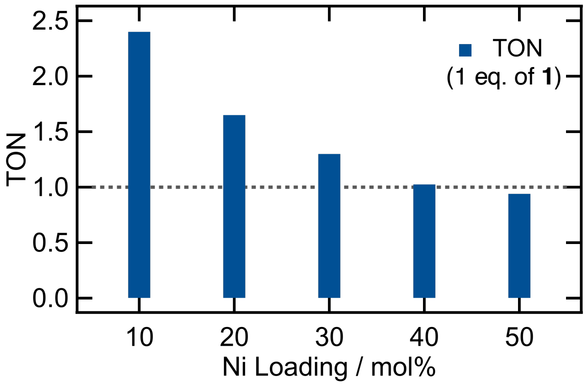

Table S7: Solvents I

entry	reaction conditions	yield
1	DMF	33%
2	DMA	23%
3	NMP	27%
4	ACN	40%
5	THF	43%
6	t-amyl-OH	38%
7	2-Me-THF	39%
8	2,2,2-Trifluoroethanol	traces

Table S8: Starting materials

entry	reaction conditions	yield
1	alkyll-I + aryl-Br	33%
2	alkyl-I + aryl-I	33%
3	alkyl-Br + aryl-Br	n.d.
4	alkyl-Br + aryl-I	n.d.
5	alkyl:aryl 1:1.5	33%
6	alkyl:aryl 1:2	31%
7	alkyl:aryl 1.5:1	43%
8	alkyl:aryl 2:1	44%
9	alkyl:aryl 3:1	55%

Table S9: Control experiments

entry	reaction conditions	yield
1	no Ni	n.d.
2	no ligand	n.d.
3	no base	n.d.
4	non-dry DMF	32%
5	non-dry TEA	34%
6	no degassing	18%

Table S10: Base

entry	reaction conditions	yield
1	triethylamine, Et_3N	33%
2	2,4,6-collidine	n.d.
3	(2,6-ditertbutyl-4-methylpyridine) dtbmpy	n.d.
4	(1,2,2,6,6-pentaamethylpiperidine) PMP	35%
5	(2,2,6,6-tetramethylpiperidine) TMP	52%
6	(4-dimethylaminopyrindine) DMAP	n.d.
7	pyridine	n.d.
8	(2-tertbutyl-1,1,3,3-tetramethylguanidine) BTMG	25%
9	(1,8-Diazabicyclo[5.4.0]undec-7-ene) DBU	n.d.
10	(triazabicyclodecene) TBD	n.d.

Further optimization

Table S11: Sacrificial reductants II

entry	reaction conditions	yield
1	$PhNHNH_2$	52%
2	$MeNHNH_2*H_2SO_4$	traces
3	PhNHNHPh	traces
4	o-tolyl-$NHNH_2$	41%
5	o-tolyl-$NHNH_2$*HCl	36%
6	mesityl-$NHNH_2$*HCl	56%
7	mesityl-$NHNH_2$	51%

Table S12: Solvents II

entry	reaction conditions	yield
1	DMF	52%
2	ACN	42%
3	t-amyl-OH	45%
4	2-Me-THF	21%

Table S13: Base equivalents II

entry	reaction conditions	yield
1	2 eq.	44%
2	4 eq.	52%
3	8 eq.	56%
4	12 eq.	57%
5	16 eq.	56%

Table S14: Reductant equivalents

entry	reaction conditions	yield
1	0.25 eq.	25%
2	0.5 eq.	53%
3	0.75 eq.	58%
4	1.0 eq.	57%
5	1.25 eq.	56%
6	1.5 eq.	52%

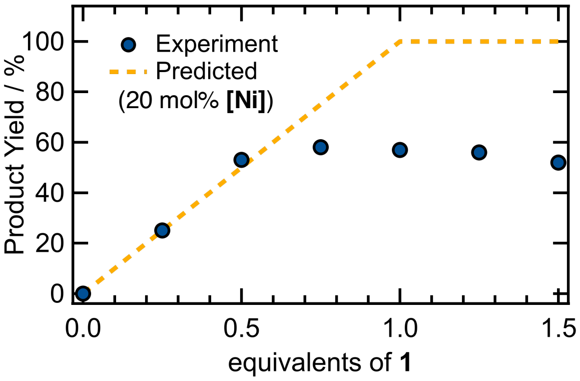

Table S15: Reaction temperature II

entry	reaction conditions	yield
1	r.t.	39%
2	40 °C	57%
3	60 °C	52%

Table S16: Catalyst loading II

entry	reaction conditions	yield
1	10 mol%	22%
2	20 mol%	57%
3	30 mol%	64%
4	40 mol%	66%
5	50 mol%	66%
6	100 mol%	69%

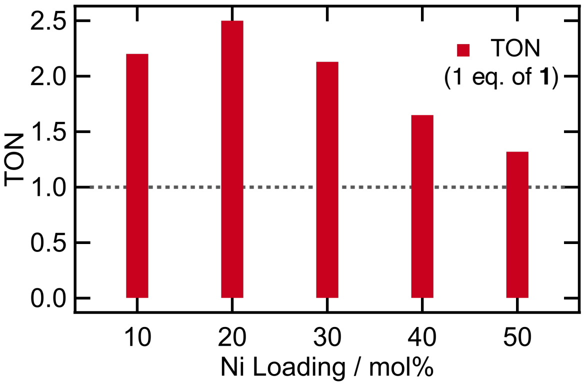

Table S17: Ni to ligand ratio

entry	reaction conditions	yield
1	1:1 ligang:Ni salt	57%
2	1:2 ligand:Ni salt	57%
3	2:1 ligand:Ni salt	35%

Table S18: Substituted phenylhydrazines

entry	reaction conditions	yield
1	para-fluoro (HCl)	30%
2	para-bromo (HCl)	38%
3	para-methoxy (HCl)	44%
4	para-trimethylfluoro (HCl)	29%
5	pentafluro (HCl)	36%
6	4-pyridiniumhydrazine	n.d.

Characterization Data

Compound 4

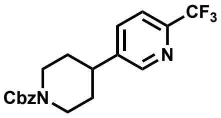

Following General Procedure A from benzyl 4-iodopiperidine-1-carboxylate (69 mg) and 5-bromo-2-(trifluoromethyl)pyridine (23 mg). Purification by flash column chromatography (10 – 50% EtOAc in hexanes) afforded 24 mg of **4** (66%) as a colorless oil.

^{1}H NMR (600 MHz, CDCl$_3$): δ 8.59 (d, *J* = 2.1 Hz, 1H), 7.73 – 7.56 (m, 2H), 7.40 – 7.30 (m, 5H), 5.16 (s, 2H), 4.38 (d, *J* = 33.4 Hz, 2H), 2.91 (s, 2H), 2.82 (tt, *J* = 12.3, 3.6 Hz, 1H), 1.87 (s, 2H), 1.66 (s, 3H).

^{13}C NMR (151 MHz, CDCl$_3$): δ 155.3, 149.3, 146.6 (q, *J* = 34.7 Hz), 144.0, 136.8, 135.4, 128.7, 128.2, 128.1, 121.6 (q, *J* = 273.9 Hz), 120.5, 119.0, 67.4, 44.5, 44.4, 40.1, 32.8.

^{19}F NMR (376 MHz, CDCl$_3$): δ -67.65.

NMR data is in agreement with literature.[5]

R$_f$ (hexanes:EtOAc = 3:1, UV) = 0.16

Compound 5

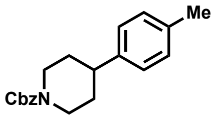

Following General Procedure B from benzyl 4-iodopiperidine-1-carboxylate (35 mg) and 4-iodotoluene (44 mg). Purification by flash column chromatography (0 – 25% EtOAc in hexanes) afforded 17 mg of **5** (55%) as a white crystalline solid.

^{1}H NMR (600 MHz, CDCl$_3$): δ 7.42 – 7.29 (m, 5H), 7.16 – 7.05 (m, 4H), 5.16 (s, 2H), 4.32 (d, *J* = 34.5 Hz, 2H), 2.88 (s, 2H), 2.64 (tt, *J* = 12.3, 3.6 Hz, 1H), 2.33 (s, 3H), 1.83 (s, 2H), 1.63 (s, 3H).

^{13}C NMR (151 MHz, CDCl$_3$): δ 155.5, 142.7, 137.0, 136.1, 129.3, 128.6, 128.1, 128.03, 126.8, 67.2, 44.8, 42.3, 33.2, 21.1.

HRMS: Calc'd for $C_{20}H_{23}NO_2$, $[M+H]^+$ 310.1807; found 310.1799

R$_f$ (hexanes:EtOAc = 6:1, UV) = 0.38

Compound 6

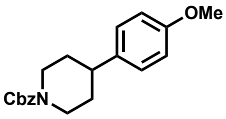

Following General Procedure B from benzyl 4-iodopiperidine-1-carboxylate (35 mg) and 4-iodoanisole (46 mg). Purification by flash column chromatography (0 – 25% EtOAc in hexanes) afforded 22 mg of **6** (68%) as a colorless oil.

^{1}H NMR (600 MHz, $CDCl_3$): δ 7.42 – 7.29 (m, 5H), 7.12 (d, *J* = 8.6 Hz, 2H), 6.86 (d, *J* = 8.6 Hz, 2H), 5.16 (s, 2H), 4.33 (d, *J* = 34.3 Hz, 2H), 3.79 (s, 3H), 2.88 (s, 2H), 2.62 (ddd, *J* = 12.2, 8.5, 3.6 Hz, 1H), 1.82 (s, 2H), 1.61 (s, 2H).

^{13}C NMR (151 MHz, $CDCl_3$): δ 158.2, 155.4, 137.9, 137.0, 128.6, 128.1, 128.0, 127.7, 114.0, 67.2, 55.4, 44.8, 41.8, 33.6, 33.4.

NMR data is in agreement with literature.[6]

R_f (hexanes:EtOAc = 2:1, UV) = 0.54

Compound 7

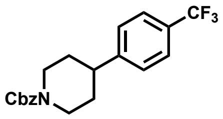

Following General Procedure A from benzyl 4-iodopiperidine-1-carboxylate (69 mg) and 1-bromo-4-(trifluoromethyl)benzene (14 µL). Purification by flash column chromatography (10 – 50% EtOAc in hexanes) afforded 17 mg of **7** (46%) as a colorless oil.

^{1}H NMR (600 MHz, $CDCl_3$): δ 7.56 (d, *J* = 8.1 Hz, 2H), 7.40 – 7.32 (m, 5H), 7.30 (d, *J* = 8.1 Hz, 2H), 5.16 (s, 2H), 4.35 (d, *J* = 34.3 Hz, 2H), 2.89 (s, 2H), 2.74 (tt, *J* = 12.3, 3.6 Hz, 1H), 1.84 (s, 2H), 1.65 (s, 2H).

^{13}C NMR (151 MHz, $CDCl_3$): δ 155.3, 149.5, 136.8, 128.5, 128.1, 128.0, 127.2, 125.6, 125.5, 125.5, 125.5, 125.1, 123.3, 67.2, 44.5, 42.5, 32.9.

^{19}F NMR (376 MHz, $CDCl_3$): δ -62.37.

NMR data is in agreement with literature.[5]

R_f (hexanes:EtOAc = 1:1, UV) = 0.40

Compound 8

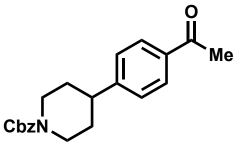

Following General Procedure B from benzyl 4-iodopiperidine-1-carboxylate (35 mg) and 4-iodoacetophenone (50 mg). Purification by flash column chromatography (0 – 25% EtOAc in hexanes) afforded 18 mg of **8** (53%) as a colorless oil.

^{1}H NMR (600 MHz, $CDCl_3$): δ 7.91 (d, *J* = 8.4 Hz, 2H), 7.43 – 7.31 (m, 5H), 7.29 (d, *J* = 8.3 Hz, 2H), 5.16 (s, 2H), 4.35 (d, *J* = 35.9 Hz, 2H), 2.90 (s, 2H), 2.74 (tt, *J* = 12.2, 3.6 Hz, 1H), 2.59 (s, 3H), 1.85 (s, 2H), 1.66 (s, 2H).

^{13}C NMR (151 MHz, $CDCl_3$): δ 197.8, 155.3, 151.1, 136.8, 135.6, 128.8, 128.5, 128.0, 127.0, 67.2, 44.5, 42.7, 32.8, 26.6.

NMR data is in agreement with literature.[5]

R_f (hexanes:EtOAc = 4:1, UV) = 0.20

Compound 9

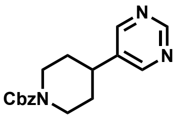

Following General Procedure A from benzyl 4-iodopiperidine-1-carboxylate (35 mg) and 5-bromopyrimidine (24 mg). Purification by pTLC (hexanes:EtOAc = 1:4) afforded 15 mg of **9** (51%) as a colorless crystalline solid.

^{1}H NMR (600 MHz, $CDCl_3$): δ 9.10 (s, 1H), 8.60 (s, 2H), 7.46 – 7.29 (m, 5H), 5.16 (s, 2H), 4.38 (d, *J* = 31.3 Hz, 2H), 3.12 – 2.80 (m, 2H), 2.72 (tt, *J* = 12.4, 3.6 Hz, 1H), 1.88 (s, 2H), 1.68 (s, 2H).

^{13}C NMR (151 MHz, $CDCl_3$): δ 157.4, 155.7, 155.3, 138.2, 136.8, 128.7, 128.2, 128.1, 67.4, 44.3, 38.2, 32.5.

HRMS: Calc'd for $C_{17}H_{19}N_3O_2$, $[M+H]^+$ 298.1556; found 298.1551

R_f (hexanes:EtOAc = 1:4, UV) = 0.39

Compound 10

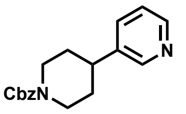

Following General Procedure General Procedure A from benzyl 4-iodopiperidine-1-carboxylate (35 mg) and 3-iodo-pyridine (20 mg). Purification by pTLC (hexanes:EtOAc = 1:1) afforded 14 mg of **10** (47%) as an off-white oil.

^{1}H NMR (600 MHz, CDCl$_3$): δ 8.52 – 8.42 (m, 1H), 7.51 – 7.47 (m, Hz, 1H), 7.43 – 7.29 (m, 3H), 7.28 – 7.19 (m, 1H), 5.16 (s, 1H), 4.35 (s, 1H), 2.90 (s, 1H), 2.71 (tt, *J* = 12.3, 3.6 Hz, 1H), 1.91 – 1.77 (m, 2H), 1.75 – 1.54 (m, 1H).

^{13}C NMR (151 MHz, CDCl$_3$): δ 155.4, 149.0, 148.1, 140.7, 136.9, 134.1, 128.6, 128.2, 128.1, 123.6, 67.3, 44.5, 40.2, 33.0.

HRMS: Calc'd for $C_{18}H_{20}N_2O_2$, $[M+H]^+$ 297.1603; found 297.1595

R_f (hexanes:EtOAc = 1:1, UV) = 0.29

Compound 11

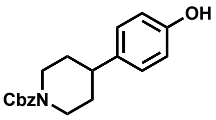

Following General Procedure A from benzyl 4-iodopiperidine-1-carboxylate (35 mg) and 4-iodophenol (44 mg). Purification by flash column chromatography (10 – 45% EtOAc in hexanes) afforded 18 mg of **11** (58%) as a colorless crystalline solid.

^{1}H NMR (600 MHz, CDCl$_3$): δ 7.45 – 7.29 (m, 5H), 7.05 (d, *J* = 8.0 Hz, 2H), 6.78 (d, *J* = 8.0 Hz, 2H), 5.16 (s, 2H), 5.11 (s, 1H), 4.31 (d, *J* = 34.7 Hz, 2H), 2.87 (s, 2H), 2.60 (tt, *J* = 12.3, 3.7 Hz, 1H), 1.80 (s, 1H), 1.59 (s, 2H).

^{13}C NMR (151 MHz, CDCl$_3$): δ 155.5, 154.3, 137.9, 137.0, 128.6, 128.1, 128.0, 127.9, 115.5, 67.3, 44.8, 41.8, 33.5.

HRMS: Calc'd for $C_{19}H_{21}NO_3$, $[M+H]^+$ 312.1600; found 312.1593

R_f (hexanes:EtOAc = 1:1, UV) = 0.56

Compound 12

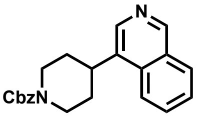

Following General Procedure A from benzyl 4-iodopiperidine-1-carboxylate (35 mg) and 4-bromoisoquinoline (21 mg). Purification by pTLC (hexanes:EtOAc = 1:1) afforded 11 mg of **12** (32%) as a colorless crystalline solid.

^{1}H NMR (600 MHz, CDCl$_3$): δ 9.16 (s, 1H), 8.41 (s, 1H), 8.05 (d, *J* = 8.3 Hz, 1H), 8.01 (d, *J* = 8.3 Hz, 1H), 7.76 (t, *J* = 8.0 Hz, 1H), 7.63 (t, *J* = 8.0 Hz, 1H), 7.44 – 7.30 (m, 5H), 5.18 (s, 2H), 4.43 (d, *J* = 31.3 Hz, 2H), 3.42 (tt, *J* = 12.1, 3.4 Hz, 1H), 3.04 (s, 2H), 2.02 (s, 2H), 1.86 (qd, *J* = 12.6, 4.3 Hz, 2H).

^{13}C NMR (151 MHz, CDCl$_3$): δ 155.4, 151.7, 140.0, 136.9, 134.2, 134.0, 130.7, 128.9, 128.7, 128.5, 128.2, 128.1, 127.1, 122.0, 67.4, 44.9, 36.4, 32.6.

HRMS: Calc'd for $C_{22}H_{22}N_2O_2$, $[M+H]^+$ 347.1760; found 347.1759

R_f (hexanes:EtOAc = 1:1, UV) = 0.34

<u>Compound 13</u>

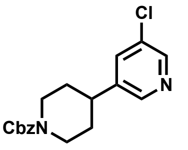

Following General Procedure A from benzyl 4-iodopiperidine-1-carboxylate (35 mg) and 3-bromo-5-chloropyridine (29 mg). Purification by pTLC (hexanes:EtOAc = 3:1) afforded 13 mg of **13** (39%) as a colorless oil.

^{1}H NMR (600 MHz, $CDCl_3$): δ 8.43 (d, *J* = 2.3 Hz, 1H), 8.35 (d, *J* = 1.9 Hz, 1H), 7.49 (t, *J* = 2.1 Hz, 1H), 7.42 – 7.30 (m, 5H), 5.15 (s, 2H), 4.36 (d, *J* = 34.5 Hz, 2H), 2.89 (s, 2H), 2.72 (tt, *J* = 12.2, 3.6 Hz, 1H), 1.84 (s, 3H), 1.63 (s, 2H).

^{13}C NMR (151 MHz, $CDCl_3$): δ 155.3, 147.0, 146.7, 142.1, 136.8, 134.2, 132.2, 128.7, 128.2, 128.1, 67.4, 44.4, 39.9, 32.8.

HRMS: Calc'd for $C_{18}H_{19}ClN_2O_2$, $[M+H]^+$ 331.1213; found 331.1210

R_f (hexanes:EtOAc = 3:1, UV) = 0.33

<u>Compound 14</u>

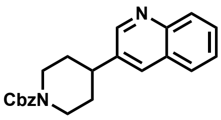

Following General Procedure A from benzyl 4-iodopiperidine-1-carboxylate (35 mg) and 3-bromoquinoline (14 µL). Purification by pTLC (hexanes:EtOAc = 1:1) afforded 17 mg of **14** (49%) as a white solid.

^{1}H NMR (600 MHz, $CDCl_3$): δ 8.81 (d, *J* = 2.3 Hz, 1H), 8.08 (d, *J* = 8.4 Hz, 1H), 7.91 (d, *J* = 2.3 Hz, 1H), 7.78 (d, *J* = 8.8 Hz, 1H), 7.68 (t, *J* = 7.6 Hz, 1H), 7.54 (t, *J* = 7.5 Hz, 1H), 7.46 – 7.30 (m, 5H), 5.18 (s, 2H), 4.40 (d, *J* = 36.4 Hz, 2H), 3.11 – 2.82 (m, 3H), 1.96 (s, 2H), 1.78 (s, 4H).

^{13}C NMR (151 MHz, $CDCl_3$): δ 155.4, 151.0, 147.3, 138.1, 136.9, 132.7, 129.3, 129.1, 128.7, 128.2, 128.2, 128.1, 127.7, 126.9, 67.3, 44.6, 40.3, 33.1, 32.8.

HRMS: Calc'd for $C_{22}H_{22}N_2O_2$, $[M+H]^+$ 347.1760; found 347.1755

R_f (hexanes:EtOAc = 1:1, UV) = 0.42

Compound 16

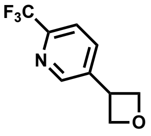 Following General Procedure A from 4-iodooxetane (18 µL) and 5-bromo-2-(trifluoromethyl)pyridine (23 mg). Purification by pTLC (hexanes:EtOAc = 2:1) afforded 8 mg of **16** (59%) as a white solid.

^{1}H NMR (600 MHz, CDCl$_3$): δ 8.69 (d, J = 2.1 Hz, 1H), 8.04 (dd, J = 8.1, 2.2 Hz, 1H), 7.73 (d, J = 8.1 Hz, 1H), 5.16 (dd, J = 8.2, 6.3 Hz, 2H), 4.73 (t, J = 6.3 Hz, 2H), 4.35 – 4.27 (m, 1H).

^{13}C NMR (151 MHz, CDCl$_3$): δ ^{13}C NMR (151 MHz, CDCl$_3$) δ 149.1, 147.5 (q, J = 36.5 Hz), 140.54, 135.53, 121.7 (q, J = 274.8 Hz), 78.07, 37.87.

^{19}F NMR (376 MHz, CDCl3): δ -67.71.

NMR data is in agreement with literature.[7]

R$_f$ (hexanes:EtOAc = 2:1, UV) = 0.35

Compound 17

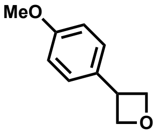 Following General Procedure A from 3-iodooxetane (18 µL) and 4-iodoanisole (23 mg). Purification by pTLC (hexanes:EtOAc = 6:1) afforded 6 mg of **17** (37%) as a yellowish oil.

^{1}H NMR (600 MHz, CDCl$_3$): δ 7.33 (d, J = 8.7 Hz, 2H), 6.91 (d, J = 8.7 Hz, 2H), 5.05 (dd, J = 8.4, 6.0 Hz, 2H), 4.80 – 4.69 (m, 2H), 4.24 – 4.13 (m, 1H), 3.81 (s, 3H).

^{13}C NMR (151 MHz, CDCl$_3$): δ 158.8, 133.8, 128.0, 114.3, 79.4, 55.5, 39.8.

NMR data is in agreement with literature.[8]

R$_f$ (hexanes:EtOAc = 6:1, UV) = 0.30

Compound 18

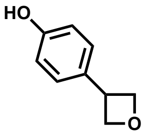 Following General Procedure A from 3-iodooxetane (9 µL) and 4-iodophenol (22 mg). Purification by pTLC (hexanes:EtOAc = 2:1) afforded 4 mg of **18** (27%) as a colorless crystalline solid.

^{1}H NMR (600 MHz, $CDCl_3$): δ 7.30 – 7.25 (m, 2H), 6.86 – 6.80 (m, 2H), 5.12 (s, 1H), 5.06 (dd, *J* = 8.4, 6.0 Hz, 2H), 4.75 (t, *J* = 6.4 Hz, 2H), 4.22 – 4.14 (m, 1H).

^{13}C NMR (151 MHz, $CDCl_3$): δ 154.8, 133.8, 128.2, 115.7, 79.5, 39.8.

R_f (hexanes:EtOAc = 2:1, UV) = 0.24

<u>Compound 19</u>

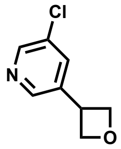

Following General Procedure from 3-iodooxetane (18 µL) and 3-bromo-5-chloropyridine (19 mg). Purification by pTLC (hexanes:EtOAc = 3:2) afforded 8 mg of **19** (47%) as a colorless oil.

^{1}H NMR (600 MHz, $CDCl_3$): δ 8.50 (d, *J* = 2.3 Hz, 1H), 8.45 (d, *J* = 2.0 Hz, 1H), 7.85 (d, *J* = 2.1 Hz, 1H), 5.12 (dd, *J* = 8.2, 6.3 Hz, 2H), 4.71 (t, *J* = 6.3 Hz, 2H), 4.27 – 4.16 (m, 1H).

^{13}C NMR (151 MHz, $CDCl_3$): δ 147.7, 146.6, 138.6, 134.1, 132.6, 78.1, 37.7.

HRMS: Calc'd for C_8H_8ClNO, $[M+H]^+$ 170.0367; found 170.0379

R_f (hexanes:EtOAc = 6:1, UV) = 0.33

<u>Compound 20</u>

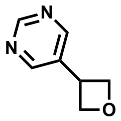

Following General Procedure A from 3-iodooxetane (18 µL) and 5-bromopyrimidine (16 mg). Purification by pTLC (hexanes:EtOAc = 1:3) afforded 4 mg of **20** (29%) as a white crystalline solid. The product was isolated in 95% purity alongside the inseparable impurity 5,5'-bipyrimidine.

^{1}H NMR (600 MHz, $CDCl_3$): δ 9.17 (s, 1H), 8.83 (s, 2H), 5.15 (dd, J = 8.3, 6.3 Hz, 2H), 4.76 (t, J = 6.4 Hz, 2H), 4.22 (tt, J = 8.3, 6.4 Hz, 1H).

^{13}C NMR (151 MHz, $CDCl_3$): δ 157.9, 155.7, 134.9, 77.9, 36.1.

HRMS: Calc'd for $C_7H_8N_2O$, $[M+H]^+$ 137.0709; found 137.0716

R_f (hexanes:EtOAc = 1:3, UV) = 0.19

Compound 22

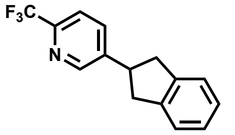

Following General Procedure A from 2-iodo-2,3-dihydro-1*H*-indene (48 mg) and 5-bromo-2-(trifluoromethyl)pyridine (23 mg). Purification by pTLC (hexanes:EtOAc = 25:1) afforded 11 mg of **22** (42%) as an off-white crystalline solid.

^{1}H NMR (600 MHz, CDCl$_3$): δ 8.67 (d, *J* = 2.2 Hz, 1H), 7.74 (dd, *J* = 8.1, 2.2 Hz, 1H), 7.61 (d, *J* = 8.1 Hz, 1H), 7.33 – 7.17 (m, 4H), 3.78 (t, *J* = 8.0 Hz, 1H), 3.45 (dd, *J* = 15.6, 8.2 Hz, 2H), 3.08 (dd, *J* = 15.6, 7.9 Hz, 2H).

^{13}C NMR (151 MHz, CDCl$_3$): δ 149.4, 146.4 (q, *J* = 34.8 Hz), 144.5, 141.9, 135.5, 127.1, 124.6, 121.8 (q, *J* = 273.7 Hz), 120.4, 119.1, 42.6, 40.6.

^{19}F NMR (376 MHz, CDCl$_3$): δ -67.72.

HRMS: Calc'd for $C_{15}H_{12}F_3N$, $[M+H]^+$ 264.1000; found 264.0997

R$_f$ (hexanes:EtOAc = 25:1, UV) = 0.37

Compound 23

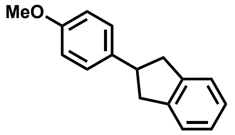

Following General Procedure A from 2-iodo-2,3-dihydro-1*H*-indene (24 mg) and 4-iodoanisole (23 mg). Purification by pTLC (100% hexanes) afforded 6 mg of **23** (27%) as a colorless oil.

^{1}H NMR (600 MHz, CDCl$_3$): δ 7.26 – 7.20 (m, 4H), 7.20 – 7.15 (m, 2H), 6.89 – 6.83 (m, 2H), 3.80 (s, 3H), 3.65 (p, *J* = 8.7 Hz, 1H), 3.32 (dd, *J* = 15.5, 8.1 Hz, 2H), 3.04 (dd, *J* = 15.5, 9.0 Hz, 2H).

^{13}C NMR (151 MHz, CDCl$_3$): δ 158.1, 143.2, 137.6, 128.1, 126.5, 124.4, 113.9, 55.4, 44.9, 41.2.

NMR data is in agreement with literature.[9]

R$_f$ (100% hexanes, UV) = 0.10

Compound 24

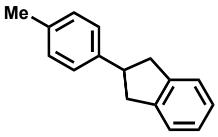

Following General Procedure A from 2-iodo-2,3-dihydro-1*H*-indene (24 mg) and 4-iodotoluene (22 mg). Purification by pTLC (100% hexanes) afforded 6 mg of **24** (29%) as a colorless oil.

¹H NMR (600 MHz, $CDCl_3$): δ 7.32 – 7.28 (m, 2H), 7.27 (d, *J* = 7.8 Hz, 2H), 7.25 – 7.22 (m, 2H), 7.19 (d, *J* = 7.9 Hz, 2H), 3.72 (p, *J* = 8.6 Hz, 1H), 3.39 (dd, *J* = 15.5, 8.2 Hz, 2H), 3.13 (dd, *J* = 15.5, 9.1 Hz, 2H), 2.40 (s, 3H).

¹³C NMR (151 MHz, $CDCl_3$): δ 143.2, 142.5, 135.8, 129.2, 127.1, 126.5, 124.4, 45.3, 41.1, 21.1.

R_f (100% hexanes, UV) = 0.62

Compound 25

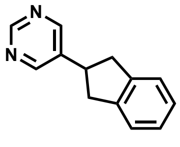

Following General Procedure A from 2-iodo-2,3-dihydro-1*H*-indene (24 mg) and 5-bromopyrimidine (32 mg). Purification by pTLC (hexanes:EtOAc = 3:1) afforded 7 mg of **25** (36%) as an off-white crystalline solid.

¹H NMR (600 MHz, $CDCl_3$): δ 9.13 (s, 1H), 8.70 (s, 2H), 7.40 – 7.17 (m, 4H), 3.72 (p, *J* = 8.0 Hz, 1H), 3.47 (dd, *J* = 15.5, 8.1 Hz, 2H), 3.12 (dd, *J* = 15.5, 7.9 Hz, 2H).

¹³C NMR (151 MHz, $CDCl_3$): δ 157.2, 155.8, 141.8, 138.6, 127.1, 124.6, 40.7, 40.31.

HRMS: Calc'd for $C_{13}H_{12}N_2$, $[M+H]^+$ 197.1079; found 197.1080

R_f (hexanes:EtOAc = 3:1, UV) = 0.28

Compound 26

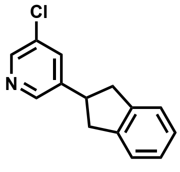

Following General Procedure A from 2-iodo-2,3-dihydro-1*H*-indene (24 mg) and 3-bromo-5-chloropyridine (38 mg). Purification by pTLC (100% hexanes) afforded 8 mg of **26** (35%) as a yellowish oil.

¹H NMR (600 MHz, $CDCl_3$): δ 8.44 (s, 2H), 7.57 (s, 1H), 7.30 – 7.15 (m, 5H), 3.70 (p, *J* = 8.2 Hz, 1H), 3.40 (dd, *J* = 15.6, 8.2 Hz, 2H), 3.06 (dd, *J* = 15.6, 8.2 Hz, 2H).

¹³C NMR (151 MHz, $CDCl_3$): δ 146.9, 146.8, 142.5, 142.0, 134.3, 132.1, 127.0, 124.6, 42.4, 40.6.

HRMS: Calc'd for $C_{14}H_{12}ClN$, $[M+H]^+$ 230.0737; found 230.0737

R_f (100% hexanes, UV) = 0.22

Compound 28

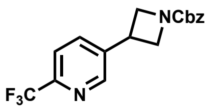 Following General Procedure A from 4-iodoazetidine-1-carboxylate (32 mg) and 5-bromo-2-(trifluoromethyl)pyridine (23 mg). Purification by pTLC (hexanes:EtOAc = 3:1) afforded 21 mg of **28** (64%) as a colorless oil.

^{1}H NMR (600 MHz, $CDCl_3$): δ 8.64 (d, *J* = 2.1 Hz, 1H), 7.89 (d, *J* = 10.5 Hz, 1H), 7.70 (d, *J* = 8.1 Hz, 1H), 7.42 – 7.29 (m, 5H), 5.14 (s, 2H), 4.50 (t, *J* = 8.8 Hz, 2H), 4.05 (dd, *J* = 8.9, 5.8 Hz, 2H), 3.89 (tt, *J* = 8.7, 5.8 Hz, 1H).

^{13}C NMR (151 MHz, $CDCl_3$): δ 156.5, 149.1, 147.4 (q, *J* = 34.8 Hz), 140.8, 136.46, 135.5, 128.7, 128.4, 128.3, 124.3, 120.7 (q, *J* = 273.8 Hz), 67.1, 56.4, 31.5.

^{19}F NMR (376 MHz, CDCl3): δ -67.72.

HRMS: Calc'd for $C_{17}H_{15}F_3N_2O_2$, $[M+H]^+$ 337.1164; found 337.1157

R_f (hexanes:EtOAc = 3:1, UV) = 0.32

Compound 29

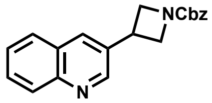 Following General Procedure A from 4-iodoazetidine-1-carboxylate (32 mg) and 3-bromoquinoline (14 µL). Purification by pTLC (hexanes:EtOAc = 1:1) afforded 12 mg of **29** (38%) as a white crystalline solid.

^{1}H NMR (600 MHz, $CDCl_3$): δ 8.89 (s, 1H), 8.14 (d, *J* = 11.1 Hz, 2H), 7.83 (dd, *J* = 8.2, 1.4 Hz, 1H), 7.73 (ddd, *J* = 8.4, 6.8, 1.4 Hz, 1H), 7.59 (ddd, *J* = 8.1, 6.8, 1.1 Hz, 1H), 7.43 – 7.29 (m, 5H), 5.16 (s, 2H), 4.55 (t, *J* = 8.8 Hz, 2H), 4.18 (dd, *J* = 8.8, 5.9 Hz, 2H), 4.01 (tt, *J* = 8.7, 5.9 Hz, 1H).

^{13}C NMR (151 MHz, $CDCl_3$): δ 156.6, 150.1, 147.2, 136.6, 134.7, 133.6, 129.8, 129.1, 128.7, 128.3, 128.2, 128.1, 127.8, 127.4, 67.1, 56.3, 31.8.

HRMS: Calc'd for $C_{20}H_{18}N_2O_2$, $[M+H]^+$ 319.1441; found 319.1455

R_f (hexanes:EtOAc = 1:1, UV) = 0.32

Compound 30

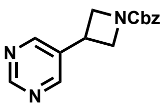 Following General Procedure A from 4-iodoazetidine-1-carboxylate (32 mg) and 5-bromopyrimidine (16 mg). Purification by pTLC (hexanes:EtOAc = 1:2) afforded 13 mg of **30** (48%) as a colorless oil.

¹H NMR (600 MHz, CDCl₃): δ 9.15 (s, 1H), 8.73 (s, 2H), 7.45 – 7.28 (m, 5H), 5.14 (s, 2H), 4.49 (t, J = 8.8 Hz, 2H), 4.08 (dd, J = 8.9, 5.8 Hz, 2H), 3.80 (tt, J = 8.8, 5.8 Hz, 1H)

^{13}C NMR (151 MHz, $CDCl_3$): δ 157.9, 156.4, 155.7, 136.4, 135.2, 128.7, 128.4, 128.3, 67.1, 56.1, 29.7.

HRMS: Calc'd for $C_{15}H_{15}N_3O_2$, $[M+H]^+$ 270.1243; found 270.1236

R_f (hexanes:EtOAc = 1:2, UV) = 0.18

Compound 31

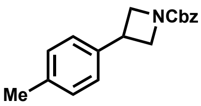

Following General Procedure A from 4-iodoazetidine-1-carboxylate (32 mg) and 4-iodotoluene (22 mg). Purification by pTLC (hexanes:EtOAc = 8:1) afforded 13 mg of **31** (46%) as a colorless oil.

^{1}H NMR (600 MHz, $CDCl_3$): δ 7.44 – 7.29 (m, 5H), 7.24 – 7.11 (m, 4H), 5.13 (s, 2H), 4.40 (t, J = 8.7 Hz, 2H), 4.04 (dd, J = 8.6, 6.1 Hz, 2H), 3.76 (tt, J = 8.7, 6.1 Hz, 1H), 2.34 (s, 3H).

^{13}C NMR (151 MHz, $CDCl_3$): δ 156.6, 139.0, 136.9, 136.8, 129.6, 128.6, 128.2, 128.1, 126.8, 66.8, 57.3, 56.7, 33.7, 21.2.

NMR data is in agreement with literature.[10]

R_f (hexanes:EtOAc = 8:1, UV) = 0.35

Compound 32

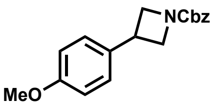

Following General Procedure A from 4-iodoazetidine-1-carboxylate (32 mg) and 4-iodoanisole (23 mg). Purification by pTLC (hexanes:EtOAc = 3:1) afforded 14 mg of **32** (47%) as a colorless oil.

^{1}H NMR (600 MHz, $CDCl_3$): δ 7.40 – 7.30 (m, 5H), 7.23 (d, J = 8.6 Hz, 2H), 6.89 (d, J = 8.7 Hz, 2H), 5.13 (s, 2H), 4.39 (t, J = 8.7 Hz, 2H), 4.02 (dd, J = 8.6, 6.2 Hz, 2H), 3.80 (s, 3H), 3.74 (tt, J = 8.8, 6.1 Hz, 1H).

^{13}C NMR (151 MHz, $CDCl_3$): δ 158.8, 156.6, 136.8, 134.1, 128.6, 128.2, 128.1, 127.9, 114.3, 66.8, 57.5, 56.9, 55.5, 33.4.

NMR data is in agreement with literature.[11]

R_f (hexanes:EtOAc = 3:1, UV) = 0.38

Compound 33

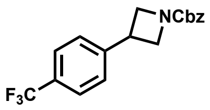

Following General Procedure A from 4-iodoazetidine-1-carboxylate (32 mg) and 1-bromo-4-(trifluoromethyl)benzene (14 µL). Purification by pTLC (hexanes:EtOAc = 4:1) afforded 10 mg of **33** (30%) as a colorless crystalline solid.

^{1}H NMR (600 MHz, $CDCl_3$): δ 7.61 (d, *J* = 8.0 Hz, 2H), 7.43 (d, *J* = 8.0 Hz, 2H), 7.40 – 7.30 (m, 5H), 5.14 (s, 2H), 4.45 (t, *J* = 8.8 Hz, 2H), 4.06 (dd, *J* = 8.8, 5.9 Hz, 2H), 3.84 (tt, *J* = 8.8, 5.9 Hz, 1H).

^{13}C NMR (151 MHz, $CDCl_3$): δ 156.6, 146.0, 136.6, 129.7, 129.5, 128.7, 128.3, 128.2, 127.3, 126.0, 125.9, 125.9, 125.9, 125.1, 123.3, 67.0, 56.4, 33.8.

^{19}F NMR (376 MHz, $CDCl_3$): δ -62.51.

HRMS: Calc'd for $C_{18}H_{16}F_3NO_2$, $[M+H]^+$ 336.1211; found 336.1202

R_f (hexanes:EtOAc = 1:1, UV) = 0.41

Compound 34

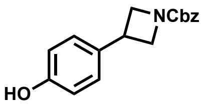

Following General Procedure A from 4-iodoazetidine-1-carboxylate (32 mg) and 4-iodophenol (22 mg). Purification by pTLC (hexanes:EtOAc = 2:1) afforded 11 mg of **34** (39%) as an off-white crystalline solid.

^{1}H NMR (600 MHz, $CDCl_3$): δ 7.42 – 7.29 (m, 5H), 7.15 (d, *J* = 8.5 Hz, 2H), 6.81 (d, *J* = 8.5 Hz, 2H), 5.80 (s, 1H), 5.14 (s, 2H), 4.39 (t, *J* = 8.8 Hz, 2H), 4.01 (dd, *J* = 8.7, 6.1 Hz, 2H), 3.71 (tt, *J* = 8.8, 6.0 Hz, 1H).

^{13}C NMR (151 MHz, $CDCl_3$): δ 156.8, 155.1, 136.7, 133.8, 128.7, 128.2, 128.2, 128.1, 115.7, 67.0, 57.4, 56.9, 33.4.

HRMS: Calc'd for $C_{17}H_{17}NO_3$, $[M+H]^+$ 284.1287; found 284.1284

R_f (hexanes:EtOAc = 2:1, UV) = 0.23

Compound 35

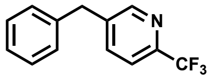

Following General Procedure A from benzyl bromide (12 µL) and 5-iodo-2-(trifluoromethyl)pyridine (41 mg). Purification by pTLC (hexanes:EtOAc = 4:1) afforded 12 mg of **35** (51%) as a colorless oil.

^{1}H NMR (600 MHz, $CDCl_3$): δ 8.69 – 8.54 (m, 1H), 7.69 – 7.55 (m, 2H), 7.33 (t, *J* = 7.6 Hz, 2H), 7.29 – 7.22 (m, 1H), 7.22 – 7.11 (m, 2H), 4.06 (s, 2H).

^{13}C NMR (151 MHz, $CDCl_3$): δ 150.5, 146.4 (q, *J* = 35.2 Hz), 140.1, 138.8, 137.6, 129.1, 127.0, 121.8 (q, *J* = 274.0 Hz), 120.4, 39.0.

^{19}F NMR (376 MHz, $CDCl_3$): δ -67.73.

NMR data is in agreement with literature.[12]

R_f (hexanes:EtOAc = 4:1, UV) = 0.75

Compound 36

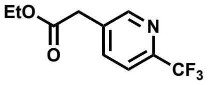

Following General Procedure A from ethyl 2-bromoacetate (11 µL) and 5-iodo-2-(trifluoromethyl)pyridine (23 mg). Purification by pTLC (hexanes:EtOAc = 5:1) afforded 7 mg of **36** (30%) as a colorless oil.

^{1}H NMR (600 MHz, $CDCl_3$): δ 8.64 (d, *J* = 2.1 Hz, 1H), 7.84 (dd, *J* = 8.2, 2.1 Hz, 1H), 7.67 (dd, *J* = 8.0, 0.8 Hz, 1H), 4.19 (q, *J* = 7.1 Hz, 2H), 3.71 (s, 2H), 1.27 (t, *J* = 7.1 Hz, 3H).

^{13}C NMR (151 MHz, $CDCl_3$): δ 170.0, 150.8, 147.3, 138.4, 133.2, 120.4, 61.7, 38.4, 14.3.

^{19}F NMR (376 MHz, $CDCl_3$): δ -67.87.

NMR data is in agreement with literature.[13]

R_f (hexanes:EtOAc = 5:1, UV) = 0.43

Encountered limitations

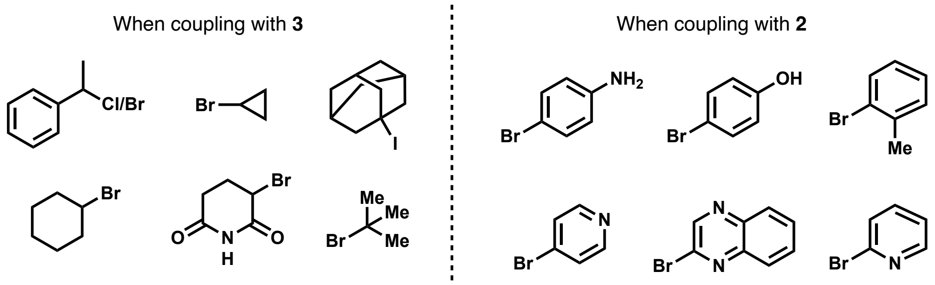

Scale-up

Phenylhydrazine conditions

To a 1L Optimax reactor, was added the benzyl 4-iodopiperidine-1-carboxylate (61.09 g, 177 mmol, 2.0 eq.) and 5-bromo-2-(trifluoromethyl)pyridine (20.00 g, 88.5 mmol, 1.0 eq.) and $NiCl_2$(dme) (3.89 g, 17.7 mmol, 0.2 eq.) and dtbbpy (4.75 g, 17.7 mmol, 0.2 eq.). DMF (870 mL) was then added from a graduated cylinder. Next, PTFE tubing was placed below the surface of the liquid and the reactor sparged with N_2 for 10 min at approximately 2 L/min. Then phenylhydrazine (8.8 mL, 88.5 mmol, 1.0 eq.) via addition funnel over 4 min and resulted in a light blue solution. TMP (60 mL, 354 mmol, 4.0 eq.) was added via the same addition funnel over 5 min and subsequently the solution change color form light blue to brown. The internal temperature was adjusted to 40 °C. The reaction was monitored for conversion by HPLC. After 5 h, all the aryl bromide starting material had disappeared but some alkyl iodide remained. The contents of the reactor were transferred to a 3 L separatory funnel, and 1 L of MTBE was added along with 1 L of 5% H_3PO_4. The layers were separated and this wash was repeated two additional times. The first wash resulted in some emulsion which was kept with the organic and resolved by the third wash. The organic layer was then washed with 500 mL of sat. $NaHCO_3$. Following this wash, the organic layer was transferred to a 2 L flask and 250 g of 60 mesh Silica was added. The contents of the flask were concentrated down to a tan powder and then added to the top of a 600 g silica plug constructed over a 2 L disposable filter frit. The height of the silica plug prior was 10 cm prior to the product containing solid being added. The silica plug was eluted with 500 mL portions of 20% EtOAc in heptane. Three portions were required to completely remove the remaining iodide starting material. Elution using 40% EtOAc in heptane resulted in the fractions containing pure product. Overall, 1 L was used to elute the product. The combined product-containing fractions were concentrated down to a yellow oil which was held at 50 °C for 24 h and then analyzed by NMR and HPLC. 22.45 g of product were obtained using this method for 69.6% isolated yield.

Zinc conditions

To the 1 L Optimax reactor was added dtbbpy (2.33 g, 8.69 mmol, 0.10 eq.), 5-bromo-2-(trifluoromethyl)pyridine (39.28 g, 174 mmol, 2.0 eq.), $MgCl_2$ (8.27 g, 86.9 mmol, 1.0 eq.), $NiCl_2$(dme) (1.91 g, 8.69 mmol, 0.1 eq.), Zn (17.05 g, 261 mmol, 3.0 eq.), and benzyl-4-iodopiperidine-1-carboxylate (30.00 g, 86.9 mmol, 1.0 eq.). Then, DMA (870 mL) was added from a graduated cylinder. Finally, pyridine (7.0 mL, 86.9 mmol, 1.0 eq.) was added, and the solution was sparged for 10 min via a subsurface PTFE tube at 2 L/min. The internal temperature on the reactor was controlled at 30 °C, and the reactor stirred at 200 rpm for a comparable energy/volume ratio to large-scale reactors. After 14 h, HPLC analysis showed complete conversion of the starting

material iodide. The reactor was cooled to 20 °C and the crude solution was transferred and filtered to remove zinc salts and other precipitates. This filtration was done with a 10 Micron disposable frit. To the filtered material was added 1 L of 5% H_3PO_4 and after it had cooled from the exothermic addition the solution was transferred to a 3 L separatory funnel where 1 L MTBE was added. The layers were separated and this wash was repeated two additional times. The first wash resulted in some emulsion which was kept with the organic and resolved by the third wash. The organic layer was then washed with 500 mL of sat. $NaHCO_3$. Following this wash, the organic layer was transferred to a 2 L flask and 250 g of 60 mesh Silica was added. The contents of the flask were concentrated down to a tan powder and then added to the top of a 600 g silica plug constructed over a 2 L disposable filter frit. The height of the silica plug prior was 10 cm prior to the product containing solid being added. The silica plug was eluted with 500 mL portions of 15% EtOAc in heptane to remove aryl bromide remaining starting material as well as several other non-polar byproducts. Three portions were required to remove these impurities completely. Elution using 40% EtOAc in heptane resulted in the fractions containing pure product. Overall, 1.5 L was used to elute the product. The combined product-containing fractions were concentrated down to a yellow oil which was held at 50 °C for 24 h and produced 11.20 g of product for 35.4% yield.

Mechanistic studies

^{19}F NMR studies

Reaction of XX with base and $NiCl_2$(dme)

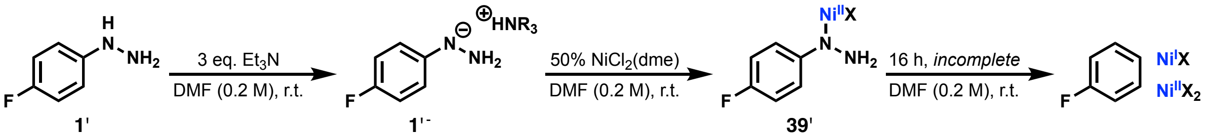

To a standard NMR tube **1'** as the HCl salt (16 mg, 0.1 mmol, 1 eq.) was added together with DMF (600 µL). Quantitative NMR was measured using 2-fluoroanisole as internal standard (-135.80 ppm). Then, TMP (50 µL, 0.3 mmol, 3 eq.) were added to the NMR tube, the solution was mixed and another spectrum was collected showing a quantitative shift of the original fluorine signal by -6.3 ppm. The addition of $NiCl_2$(dme) (11 mg, 0.05 mmol, 0.5 eq.) and dtbbpy (13.4 mg, 0.05 mmol, 0.5 eq.) introduced paramagnetism to the ^{19}F spectra indicated by signal broadening and decreased signal to noise. Nonetheless, a peak shift of +2.9 ppm can be observed along with a small shoulder. Additionally, a new signal appeared at -113 ppm that can be attributed to fluorobenzene. After 16 h this signal is more intense whereas the remaining hydrazine-catalyst adduct **39'** and additional species with a similar chemical shift are present. Even though signal broadening and signal to noise improved, quantification is not possible.

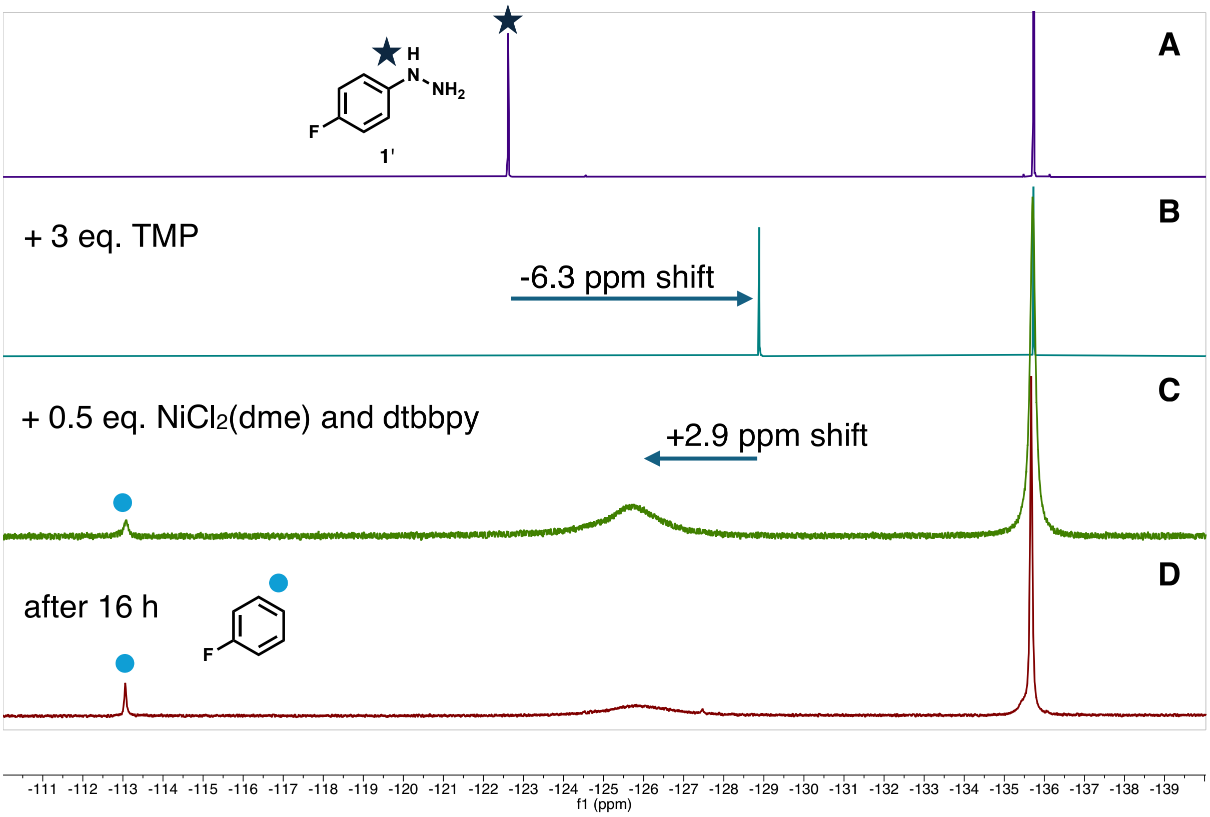

Figure S1. ^{19}F NMR of 1' in DMF (A), after addition of TMP (B), after addition of [Ni] (C), and after 16 h (D). Internal standard: 2-flouroanisole (-135.80 ppm).

In a control experiment, mixing **1'** and $NiCl_2$(dme) showed no shift of the fluorine peak but only broadening and worse signal to noise due to paramagnetism.

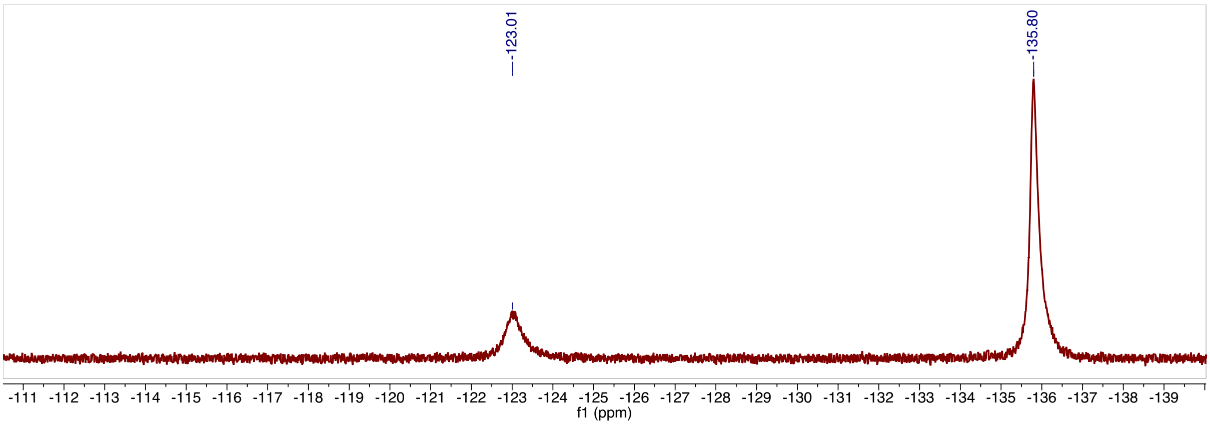

Figure S2. ^{19}F NMR of 1' and $NiCl_2$(dme) in DMF. Internal standard: 2-flouroanisole (-135.80 ppm).

Conducting the same experiments with Et_3N as base yielded similar results.

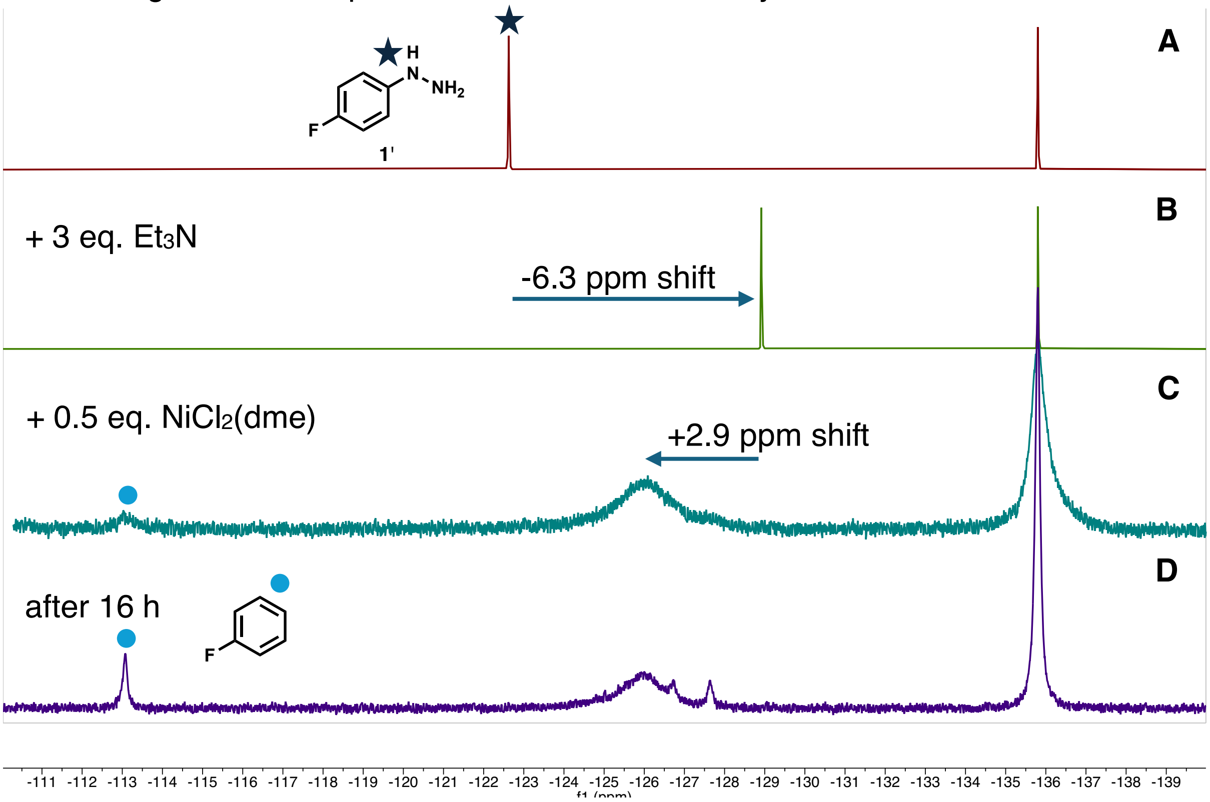

Figure S3. ^{19}F NMR of 1' in DMF (A), after addition of Et_3N (B), after addition of $NiCl_2$(dme) (C), and after 16 h (D). Internal standard: 2-flouroanisole (-135.80 ppm).

Separating activation and reactions of the single coupling partners

Under conditions of General Procedure A, phenylhydrazine **1** was replaced with 4-fluorophenylhydrazine hydrochloride **1'** (16 mg, 0.1 mmol, 1 eq.). In four separate reactions, it was converted without coupling partner, only **2**, only **3**, and both **2** and **3**.

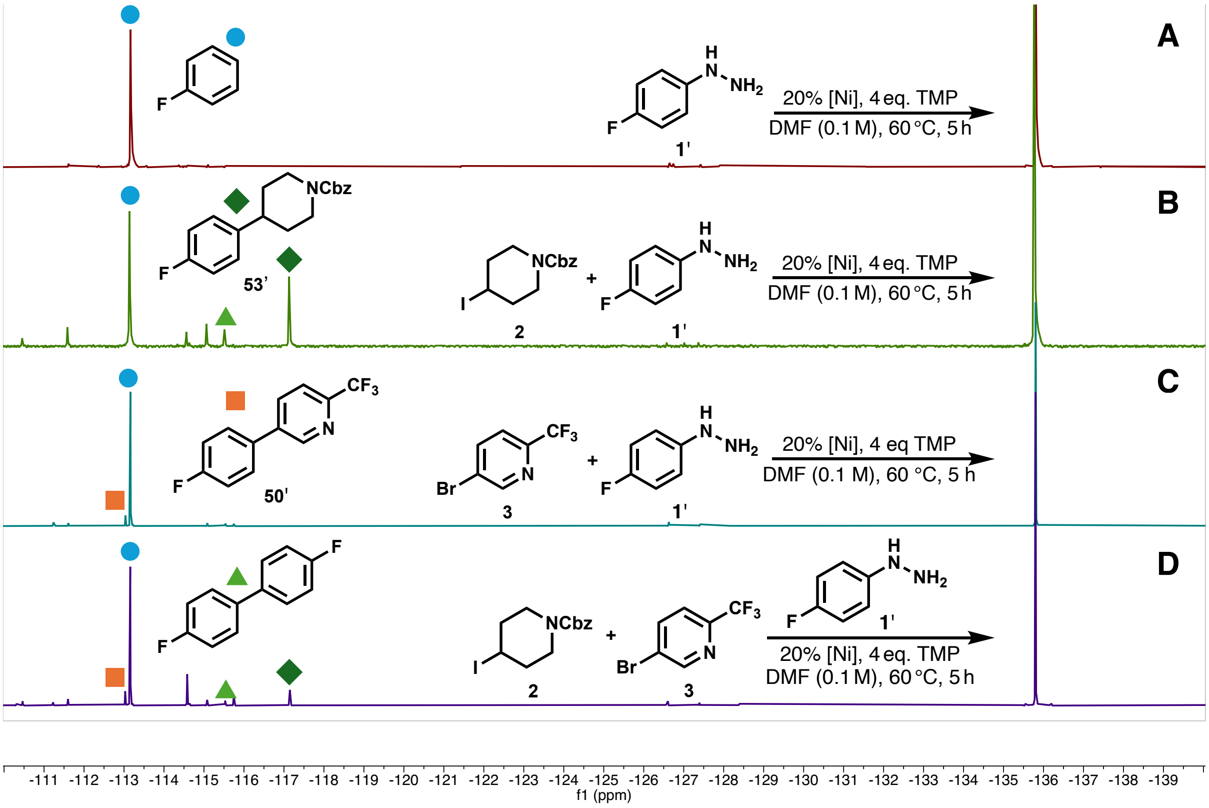

Figure S4. ^{19}F NMR of the reaction of **1'** under standard conditions (A), **1'** and **2** under standard conditions (B), **1'** and **3** under standard conditions (C), and **1'**, **2**, and **3** under standard conditions (D). Internal standard: 2-flouroanisole (-135.80 ppm).

In the reaction without both aryl bromide and alkyl iodide, only 4-fluorohydrazine could be detected after 5 h at 60 °C. According to the quantitative ^{19}F method (40 s D1 delay, 16 scans), 0.06 mmol fluorobenzene were formed corresponding to a yield of 60%. Next, alkyl iodide **2** was reacted under the given conditions. The yield of sp^2-sp^3 **53'** coupling product is 15%, while 31% fluorobenzene and 4% 4,4'-difluorobiphenyl could be detected alongside different minor side products. The reaction of **1'** with **3** gave 59% fluorobenzene, 5% of the heterocoupling product, and 65% homocoupling. In the full reaction, 44% fluorobenzene was formed alongside each 5% of **50'** and **53'**, traces of 4,4'-difluorobiphenyl as well as 13% of the desired product **4** and 60% aryl heterocoupling.

UV-vis studies

UV-vis spectroscopy was conducted using a BioMate 3S (Thermo Scientific) single beam spectrometer, air-free quartz cuvettes, and a spectral range of 350 – 900 nm. A background of pure DMF was measured prior each series of experiments. The following figures all consist of an initial solution (left), a single change to the system (middle) and the difference spectrum (right). Figures S5 to S7 show control experiments to understand background effects of [Ni] and **1** which are both UV active.

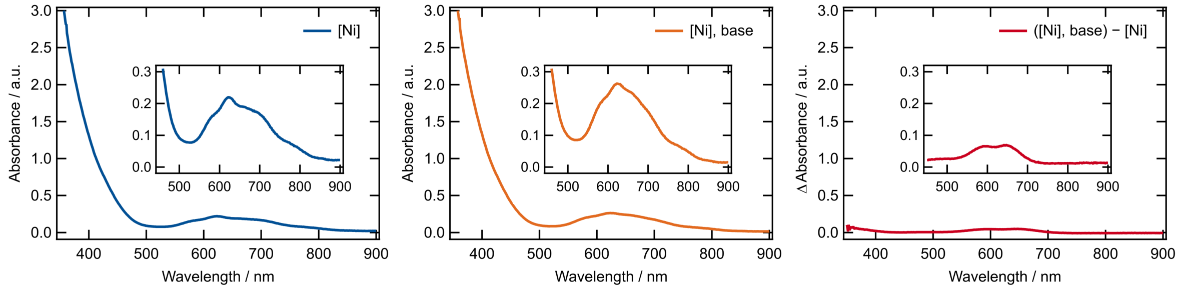

Figure S5. UV-vis spectra of [Ni] in DMF (left), after addition of base (middle) and the difference spectrum.

Adding base to [Ni] slightly changes the coordination environment of which becomes apparent through two small peaks at 580 and 650 nm respectively.

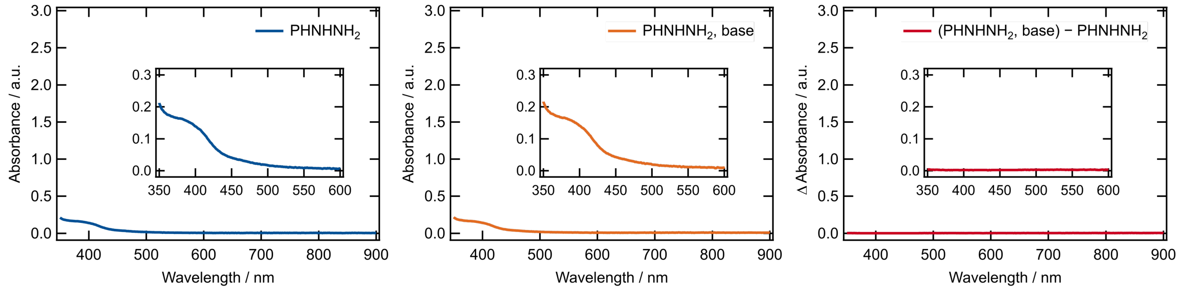

Figure S6. UV-vis spectra of **1** in DMF (left), after addition of base (middle) and the difference spectrum.

In contrast, adding base to **1** does not change its overall low absorbance.

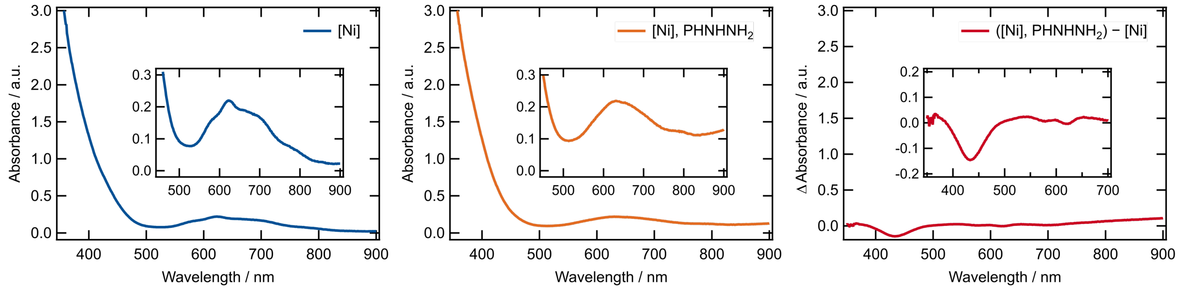

Figure S7. UV-vis spectra of [Ni] in DMF (left), after addition of **1** (middle) and the difference spectrum.

Adding **1** to [Ni] in DMF has a bigger effect than adding base and leads to the decrease of an absorption feature at 440 nm.

To study the role of **1** in the reduction of Ni, the addition of base to a solution of [Ni] and **1** was tested.

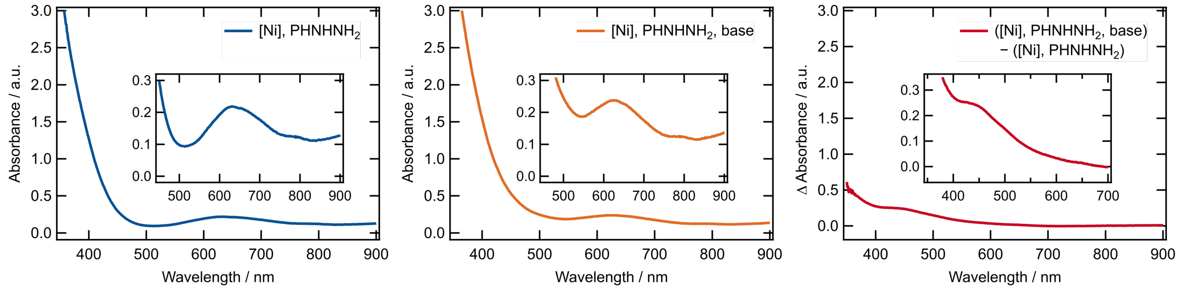

Figure SX. UV-vis spectra of [Ni] and **1** in DMF (left), after addition of base (middle) and the difference spectrum.

The difference spectrum shows the appearance of a shoulder at 450 nm which is indicative for dtbbpyNi^{II}(R)X complexes, where R is a strongly σ-donating ligand.[14,15].

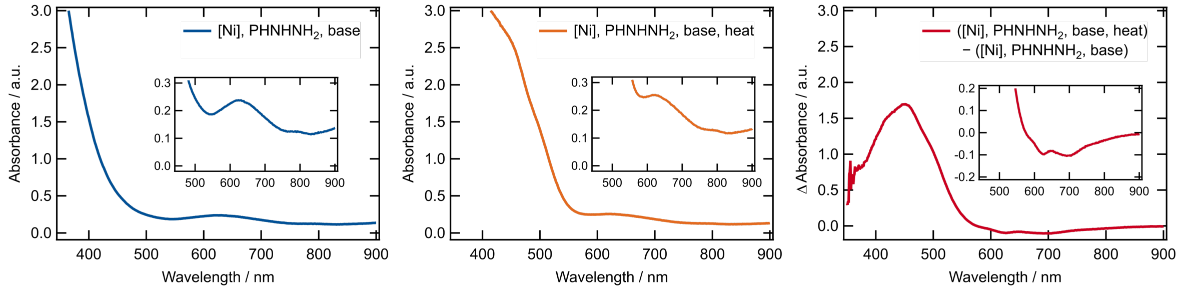

Figure S8. UV-vis spectra of [Ni], **1**, and base in DMF (left), after heating to 60 °C for 10 min (middle) and the difference spectrum.

Upon heating to 60 °C for 10 min, this shoulder grows to a distinct signal in the difference spectrum consisting of a major feature at 450 nm with shoulders at 420 and 500 nm.

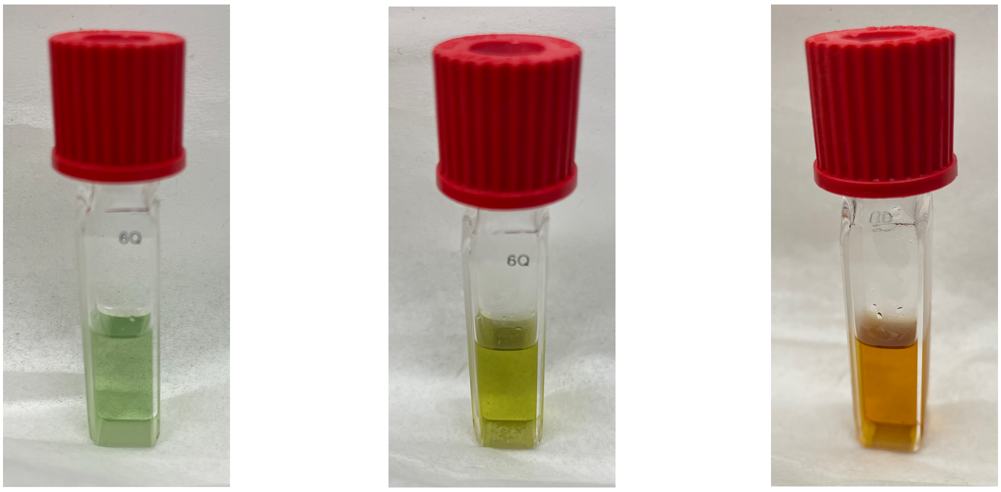

Figure S9. Reaction solution of [Ni] and **1** in DMF (left), after addition of base (middle) and heating for 10 min (right).

Photos of the air-free cuvettes document this experimental series and resemble those of the standard reaction in Figure 2 of the main manuscript.

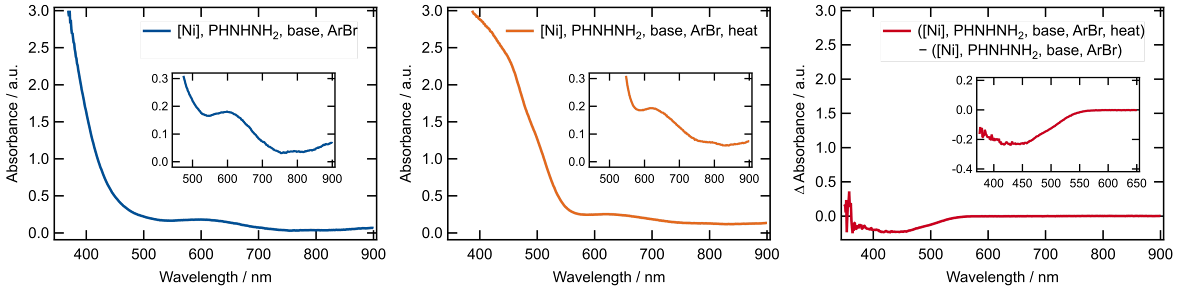

Figure S10. UV-vis spectra of ArBr-quench of a solution of [Ni], **1**, and base (left), of a solution of [Ni], 1, and base that has been heated to 60 °C for 10 min prior to ArBr-quench (middle) and the difference spectrum.

Reaction calorimetry

Reaction calorimetry was conducted in temperature scanning (TSR) and isothermal (iso) mode in an Omnical SuperCRC reaction microcalorimeter. In it, the heat flow around the reaction vessel can continuously be monitored. The calorimeter features ten channels, five on side A and five on side B. One channel on each side was dedicated to a reference vial. When using one channel for a solvent blank and another one for thermal equilibration of the reactant that is used to initiate the reaction, six reaction vials could be prepared and monitored simultaneously. Reaction vessels were 16 mL spectrum-cap vials, which were each stirred using 1 cm cross stir bars at 600 rpm. The temperature was controlled using the internal heating device and an external temperature control unit (TCU, Polyscience AP15R-30-A11B) with thermal bath of 4:1 ethylene glycol to water. Temperature ramping experiments were conducted with a 0.3 °C/min ramp rate. A heat flow data point was collected every 0.05 min (3 s).

Example TSR Calorimetry Procedure

The following sample procedure is given for the model reaction between **2** and **3** shown below. This procedure was adapted for reactions between single coupling partners and phenylhydrazine as well as by changing the mass or volume added of each reaction component, accordingly.

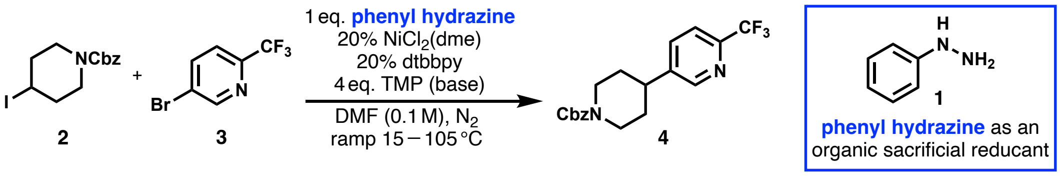

1. Calorimeter preparation

The calorimeter temperature was set to isothermally stabilize at 15 °C. The heat flow was monitored continuously until it equilibrated with no heat flow for all channels. The temperature was chosen because it is below the onset temperature of the reaction, allowing the samples to equilibrate without undesired background reactivity. Nonetheless, base was stored in a separate vial and used to initiate the reaction as elimination of iodide in **2** can occur.

2. Preparation for hydrazine system

To a 16 mL oven dried vial charged with a 1 cm teflon-coated cross stir bar, **3** (113.0 mg, 0.5 mmol, 1 eq.) was added together with $NiCl_2$(dme) (22.0 mg, 0.1 mmol, 0.2 eq.) and dtbbpy (26.8 mg, 0.1 mmol, 0.2 eq.). The vial was sealed with a PTFE septum-cap, followed by three cycles of evacuation/backfill on a Schlenk line with dry N_2. Then, different liquid components were added under N_2, after purging them with dry N_2 for at least 30 min. First, 4.5 mL of anhydrous DMF were added, followed by **2** (112 µL, 172.5 mg, 0.5 mmol, 1 eq.) and **1** (49 µL, 54.0 mg, 0.5 mmol, 1 eq.). The mixture was homogenized using a vortex stirrer, affording a green solution.

The reaction vials were quickly added to the calorimeter channels A2 – A4 and B1 – B3. Channels A1 and B4 were dedicated to a solvent-only blank and base (tetramethylpyridine, TMP) respectively. The heat flow was monitored until it stabilized for all channels. Then, TMP (340 µL, 282.5 mg, 2.0 mmol, 4 eq.) tempered the in B4 was added to each reaction. Using the program setting of the TCU, the temperature was then ramped from 15 °C to 105 °C over a period of 300 minutes (0.3 °C/min) and the data recorded (datapoint collected every 3 s).

Once the ramp was completed, the TCU was set to isothermal mode. After the heat flow returned to zero, the tau-correction was applied. The samples were removed from the calorimeter and analyzed using UPLC with acetanilide as internal standard. Heat flow data from TSR was processed using python scripts developed in our lab.[16]

3. Changes for measuring the Zn system

To measure TSR of the cross-coupling using Zn as sacrificial reductant shown below, a modified protocol to initiate the reaction was necessary. Preliminary experiments showed that a solution containing all reaction components was unstable even at 0 °C.

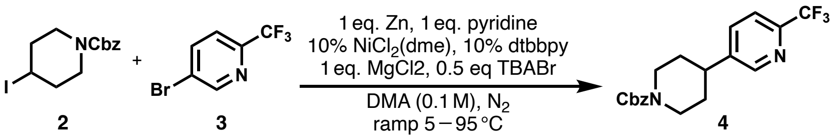

Initially, the idea was to initiate the reaction by addition of the Ni catalyst in a stock solution but, still, the solution containing Zn and the starting materials was unstable. Finally, a protocol was developed in which solid Zn dust was added to initiate the reaction shortly after the temperature ramp was started and the initial baseline drift of the calorimeter stopped. To add the Zn, a 1 mL syringe equipped with a 16G needle was used. After weighing the Zn (+10% extra to compensate for losses of the injection method) directly into the syringe equipped with a 21G needle, it was evacuated/backfilled several times and kept under dry N_2. The 16G needle was capped with rubber and evacuated/backfilled in the same manner. Immediately before injection, the needles were switched quickly to minimize O_2 getting in. The injection needle was placed on top of the septa cap of the reaction vial before it was used to pierce both its rubber cap and the septum.

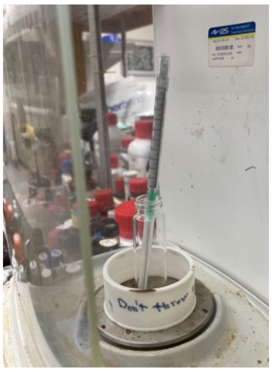

1) Weighing Zn dust into 1 mL syringe

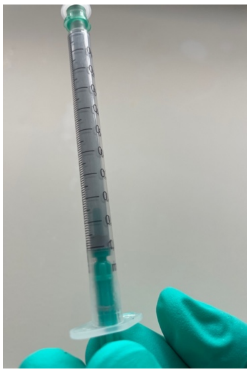

2) Zn at the bottom of the syringe

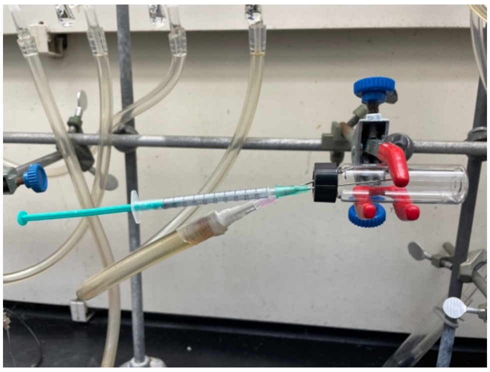

3) Syringe connected to Schlenk line through vial

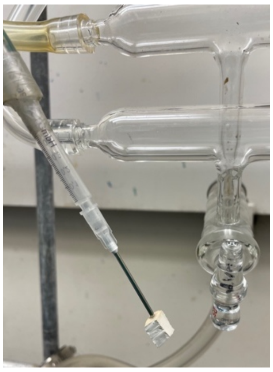

4) Capped 16G needle connected to Schlenk line

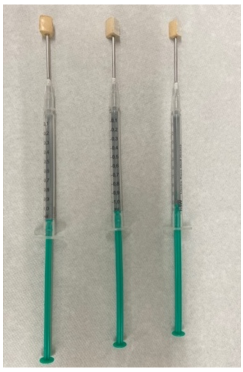

5) Syringes ready for injection

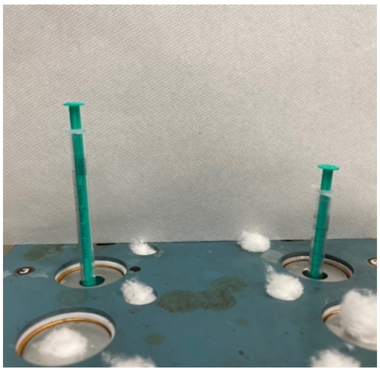

6) Syringe on top of septum (left) and after injection (right).

To a 16 mL oven dried vial charged with a 1 cm teflon-coated cross stir bar, **3** (226.0 mg, 1.0 mmol, 2 eq.) was added together with $NiBr_2$(dme) (15.4 mg, 0.05 mmol, 0.1 eq.), dtbbpy (13.4 mg, 0.05 mmol, 0.1 eq.), TBABr (80.6 mg, 0.25 mmol, 0.5 eq.), and $MgCl_2$ (47.5 mg, 0.5 mmol, 1 eq.). The vial was sealed with a PTFE septum-cap, followed by three cycles of evacuation/backfill on a Schlenk line with dry N_2. Then, different liquid components were added under N_2, after purging them with dry N_2 for at least 30 min. First, 4.8 mL of anhydrous DMA were added, followed by **2** (112 µL, 172.5 mg, 0.5 mmol, 1 eq) and pyridine (40 µL, 39.5 mg, 0.5 mmol, 1 eq.). The mixture was homogenized using a vortex stirrer, affording a blue/green solution.

The reaction vials were quickly added to the calorimeter channels A2 – A4 and B1 – B3. The heat flow was monitored until it stabilized for all channels. Using the program setting of the TCU, the temperature was then ramped from 5 °C to 95 °C over a period of 300 mins (0.3 °C/min) and the data recorded (datapoint collected every 3 s). The Zn dust was added to initiate the reaction shortly after the temperature ramp was started and the initial baseline drift of the calorimeter stopped at 8 – 9 °C.

Once the ramp was completed, the TCU was set to isothermal mode. After the heat flow returned to zero, the tau-correction was applied. The samples were removed from the calorimeter and analyzed using UPLC with acetanilide as internal standard.

4. Measurements in isothermal mode

The procedures for reaction preparation and sampling in isothermal mode were the same described above. However, the calorimeter was equilibrated at 60 °C instead of 15 °C (or 5 °C in case of Zn). Then, the samples were stabilized at this temperature in the calorimeter, before either base or Zn were used to initiate reactions in the hydrazine- or Zn-based system respectively.

Additionally, in the case of Figure 4A of the main manuscript, **1**, [Ni], and base were equilibrated in the calorimeter without adding **2** or **3**. No apparent heat production (over 40 min) was detected prior to time zero. Then, either **2**, **3**, or both were added in minimal DMF.

5. Reaction overview and additional data

Table S19: All hydrazine reactions (green) use **1** (0.5 mmol), [Ni] (0.1 mmol, except first entry), and TMP (2.0 mmol). All Zn reactions (grey) use [Ni]' (0.05 mmol), $MgCl_2$ (0.5 mmol), TBABr (0.25 mmol), pyridine (0.5 mmol), and Zn (1.5 mmol). TSR = temperature scanning, iso = isothermal.

Alkyl-I (mmol)	Aryl-Br (mmol)	Pdt. **4** (mmol)	Energy (J)	ΔH_{all} (kcal/mol)	Where is this?	Note
0	0	0	2.1	1.0	Fig. S11A – No [Ni]	TSR
0	0	0	3.4	1.6	Fig. S11A – No Alk-I / No Ar-Br	TSR
0.5	0	0	86.4	41.3	Fig. 4B – No Alk-I	TSR
0	0.5	0	103.7	49.6	Fig. 4B – No Ar-Br	TSR
0.5	0.5	0.29	172.6	82.5	Fig. 3B and 4B – PhHy Standard<br>Fig. 3D – 600 rpm	TSR
0.5	0.5	0.29	177.5	84.8	Fig. 3D – 400 rpm	TSR
0.5	0.5	0.29	181.0	86.5	Fig. 3D – 200 rpm	TSR
0.5	0.5	0.27	-	-	Fig. 3A and 4A – PhHy Standard	iso
0.5	0	0	-	-	Fig. 4A – No Ar-Br	iso
0	0.5	0	-	-	Fig. 4A – No Alk-I	iso
0.5	1.0	0.31	130.9	62.6	Fig. 3B – Zn Standard	TSR
0.5	1.0	0.33	339.5	162.3	Fig. 3E – 600 rpm<br>Fig. S11B – Zn Standard	TSR
0.5	1.0	0.33	94.0	44.9	Fig. 3E – 400 rpm	TSR
0.5	1.0	0.28	134.7	64.4	Fig. 3E – 200 rpm	TSR
0.5	0	0	96.9	46.3	Fig. S11B – No Ar-Br	TSR
0	1.0	0	183.3	87.6	Fig. S11B – No Alk-I	TSR
0.5	1.0	0.06	-	-	Fig. 3A – Zn Standard	iso

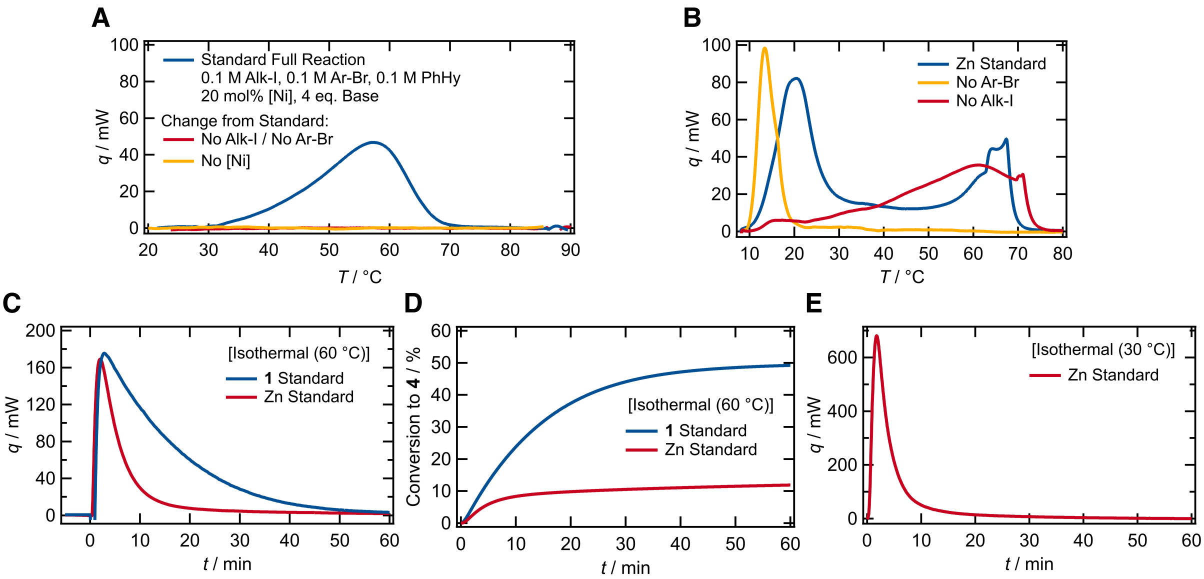

Figure S11. (A) Additional control experiments (TSR) of the system using **1**. (B) Control experiments (TSR) of the system using Zn. (C/D) Isothermal comparison of the systems using Zn (14% yield) and **1** (55% yield) at 60 °C. (E) Isothermal reaction of the systems using Zn (58% yield) at 30 °C.

6. Initial safety assessment

$$Adiabatic\ temperature\ rise = \frac{Energy\ Released\ by\ Reaction}{Heat\ Capacity\ of\ the\ System} = \frac{\int heat\ flow}{n_{all} \cdot C_{p,solvent}}$$

$$= \frac{172.6\ \mathrm{J}}{0.0037\ \mathrm{mol} \cdot 150\ \mathrm{J/(mol \cdot K)}} = 17\ \mathrm{K}\ \equiv 17\ \mathrm{°C}$$

$$Maximum\ heat\ transfer = \frac{Maximum\ Heat\ Flow}{Total\ Reaction\ Volume} = \frac{46.3\ \mathrm{mW}}{5\ \mathrm{mL}} = 9\ \mathrm{W/L}$$

$$Energy\ released\ per\ reactants = \frac{Energy\ Released\ by\ Reaction}{Mass\ of\ Reactants} = \frac{\int heat\ flow}{\sum m_{reactants}}$$

$$= \frac{172.6\ \mathrm{J}}{0.631\ \mathrm{g}} = 274\ \mathrm{J/g}$$

$$Energy\ released\ per\ reactants = \frac{\int heat\ flow}{\sum m_{reactants} + m_{solvent}} = 32\ \mathrm{J/g}$$

Table S20: Safety data calculated from TSR measurements.

Reaction under TSR conditions	ATR	max. heat transfer	Energy released per reactants	
			With solvent	Without solvent
Phenylhydrazine	17 °C	9 W/L (35 isothermal)	274 J/g	32 J/g
Zn average	12 °C	17 W/L (36 isothermal)	188 J/g	24 J/g
Zn extreme	32 °C	-	488 J/g	63 J/g

Elementary Step Analysis of Proposed Mechanism

The possibility of **42** to decompose on its own[17,18] or be captured by Ni to **44** in Cycle B led us to more closely examine the fate of Ni with and without considering Cycle B as part of the overall catalytic picture. The elementary steps for each case are denoted below, beginning with **38** (grey box) and **1** in Cycle A. Note that the base is ignored for simplicity.

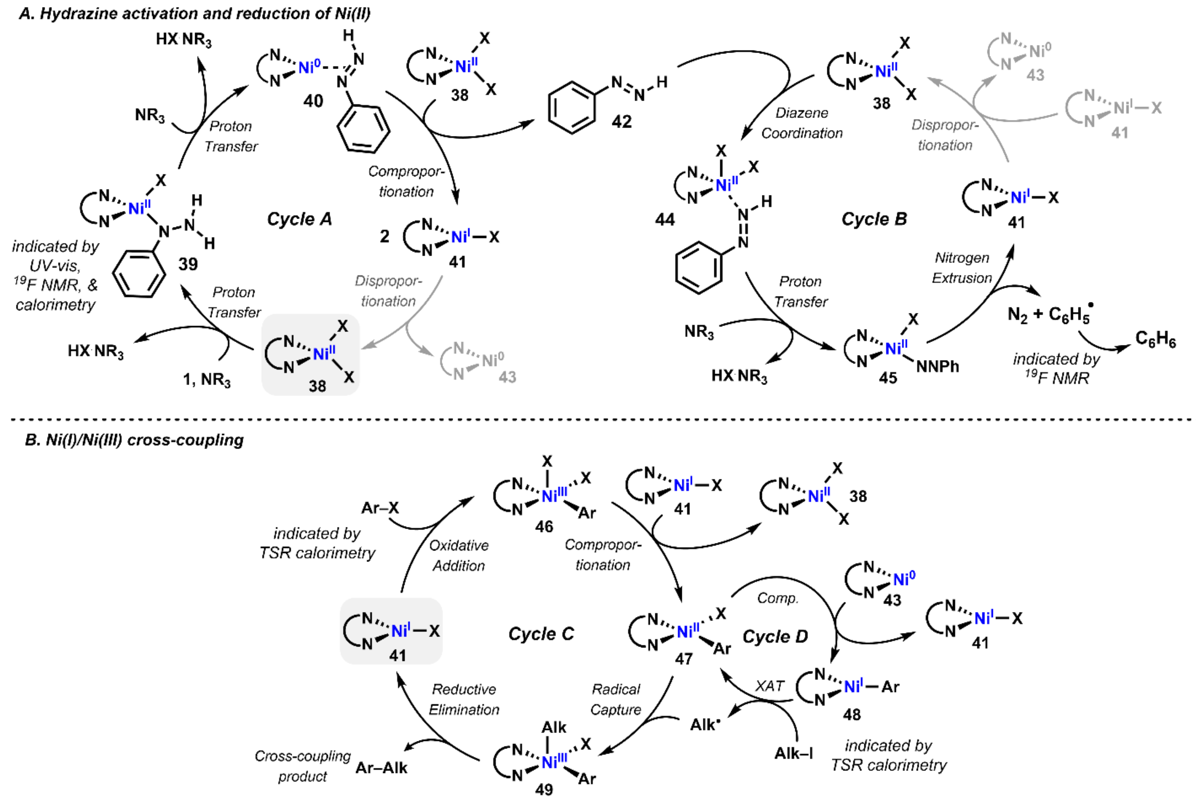

Case 1: Considering cycles A, C, and D.		
Cycle A		
**38** + **1**	→	**39**
**39**	→	**40**
**40** + **38**	→	**41** + **41** + **42**
**41** + **41**	→	**38** + **43**
Overall A: **38** + **1** → **42** + **43**		
Cycle C		
**41** + Ar–X	→	**46**
**46** + **41**	→	**38** + **47**
**47** + Alk$^{\cdot}$	→	**49**
**49**	→	**41** + Ar–Alk
Overall C: **41** + Ar–X + Alk$^{\cdot}$ → **38** + Ar–Alk		
Cycle D		
**47** + **43**	→	**41** + **48**
**48** + Alk–I	→	**47** + Alk$^{\cdot}$
Overall D: **43** + Alk–I → **41** + Alk$^{\cdot}$		
Overall Cycles A, C, and D		
**1** + Ar–X + Alk–I → **42** + Ar–Alk		

Case 2: Considering cycles A, B, C, and D.		
Cycle A		
**38** + **1**	→	**39**
**39**	→	**40**
**40** + **38**	→	**41** + **41** + **42**
**41** + **41**	→	**38** + **43**
Overall A: **38** + **1** → **42** + **43**		
Cycle B		
**38** + **42**	→	**44**
**44**	→	**45**
**45**	→	**41** + N_2 + $C_6H_5^{\cdot}$
**41** + **41**	→	**38** + **43**
Overall B: **41** + **42** → **43** + N_2 + $C_6H_5^{\cdot}$		
Cycle C		
**41** + Ar–X	→	**46**
**46** + **41**	→	**38** + **47**
**47** + Alk$^{\cdot}$	→	**49**
**49**	→	**41** + Ar–Alk
Overall C: **41** + Ar–X + Alk$^{\cdot}$ → **38** + Ar–Alk		
Cycle D		
**47** + **43**	→	**41** + **48**
**48** + Alk–I	→	**47** + Alk$^{\cdot}$
Overall D: **43** + Alk–I → **41** + Alk$^{\cdot}$		
Overall Cycles A, B, C, and D		
**41** + **1** + Ar–X + Alk–I → **43** + Ar–Alk + N_2 + $C_6H_5^{\cdot}$		

While cycles A, C, and D together exhibit clean catalytic turn-over, the inclusion of cycle B results in a net reductive process where Ni^{I} **41** must accumulate to return **43** (possibly through the disproportionation step **41** + **41** → **38** + **43**). However, the accumulation of **41** likely results in the competitive dimerization process depicted below[19–21] and marks a pathway to Ni consumption over the course of the reaction.

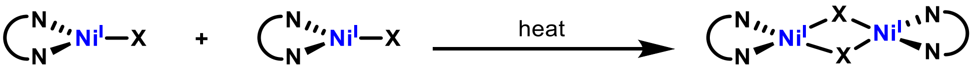

41 **41** **41-dimer** (precipitate)

Therefore, reducing the likelihood of cycle B (perhaps by increasing the self-decomposition rate of diazene **42**) may improve turnover and product yields.

Further experiments

Alkyl iodide activation with Zn

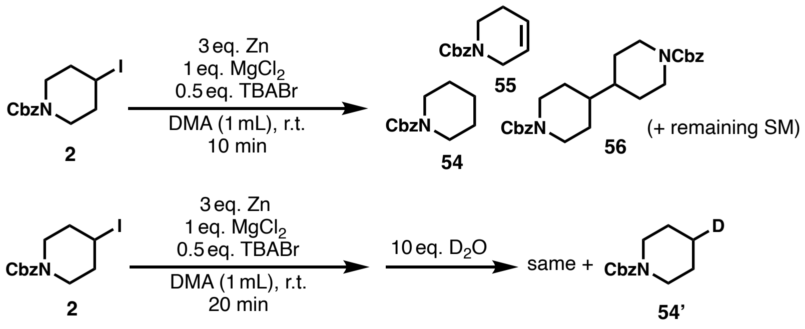

To investigate the origin of difficulties studying the Zn-based reaction with isothermal calorimetry, activation of the alkyl iodide in absence of Ni catalyst was investigated. Therefore, **2** (35 mg, 0.1 mmol, 1 eq.), Zn (20 mg, 0.3 mmol, 3 eq.), $MgCl_2$ (10 mg, 0.1 mmol, 1 eq.), and TBABr (16 mg, 0.05 mmol, 0.5 eq.) were mixed in DMF (1 mL) at room temperature. After 10 min, several products were detected that indicate Zn insertion into the alkyl iodide bond. To confirm this, D_2O (18 µL, 10 eq.) was added after another 10 min and the quench towards the deutero dehalogenation product **54'** was confirmed.

TEMPO trap experiments

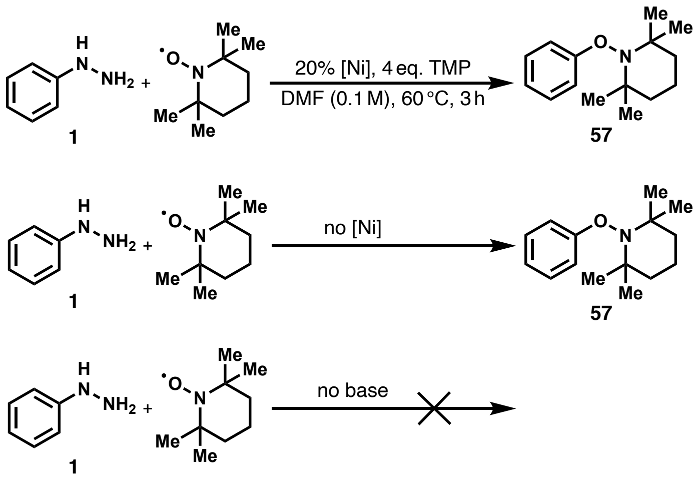

To probe if decomposition of **1** proceeds via radical or anionic pathway, TEMPO was added to the reagents involved in the catalyst activation cycle. Under these conditions, the radical recombination product **57** of TEMPO and phenyl released from **1** was observed. In a control experiment without catalyst, **57** was observed, too. In absence of base, **57** was not observed. This indicates that deprotonation of **1** is necessary to react with TEMPO rather than H* abstraction.

Ni^0 trapping

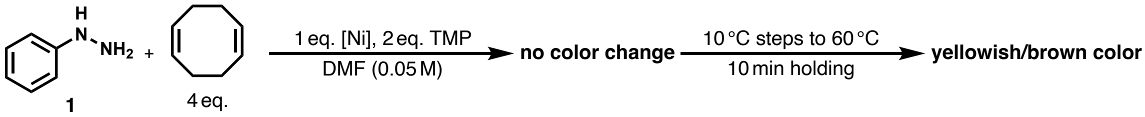

To detect if the major Ni species present in hydrazine activation is Ni(0), a typical trapping experiment was conducted. To a solution of **1** (5 µL, 0.05 mmol, 1 eq.), $NiCl_2$(dme) (11 mg, 0.05 mmol, 1 eq.), dtbbpy (13 mg, 0.05 mmol, 1 eq.), and TMP (17 µL, 0.1 mmol, 2 eq.) in DMF (1 mL), 1,5-cyclooctadiene (24 µL, 0.2 mmol, 4 eq.) was added. Since the characteristic deep purple color of dtbbpyNi(0)COD was not observed, the temperature was ramped in steps of 10 °C up to 60 °C holding each temperature for 10 min. The color change was the usual yellowish/brown observed for the hydrazine system.

Slow addition experiments

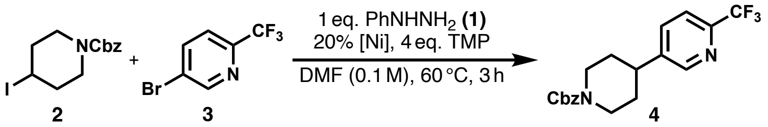

In the first experiment, **2** and **1** were pre-mixed in 70 µL DMF and added in portions of 10 µL every 15 min over a period of 150 min. After another 30 min, the yield was determined to be 44% by UPLC.

In the second experiment, TMP and **1** were pre-mixed in 20 µL DMF and added in portions of 10 µL every 15 min over a period of 150 min. After another 30 min, the yield was determined to be 50% by UPLC.

Frequently asked questions

1. What are the major by-products using hydrazine sacrificial reductant?

Generally, the expected side products of Ni-catalyzed cross coupling can be observed, i.e. protodehalogenation of the alkyl, homocoupling of the aryl. Additionally, secondary alkyl iodides undergo elimination at elevated temperatures which lead to the choice of TMP base. The use of phenylhydrazine as a sacrificial reductant leads to coupling of phenyl radical with both aryl and alkyl starting materials. Switching to the sterically hindered mesityl hydrazine dramatically reduced the undesired coupling with alkyl radicals whereas highly activated aryl like 5-bromo-2-(trifluoromethyl)pyridine will still couple with the mesityl radical.

2. Are the reactions air and/or moisture sensitive?

As demonstrated in the screening, the yield is diminished if the reaction is run under air. The sensitivity towards moisture is less pronounced but best results will be achieved with reagents of high quality.

3. Do the reaction solutions remain homogeneous at all times?

Generally, the reactions demonstrated in the scope of this paper are homogeneous. However, solids can be found after cooling the reaction solution to room temperature in some cases. Either way, the advantage of this reaction is that it avoids the use of high-density metal powders and completely insoluble inorganic additives, which make stirring/homogeneity difficult, especially for scale-up.

4. Does the order of addition of reactants matter?

The base is added last to initialize the reaction which gives a convenient on/off switch. Other than that, the order of addition did not play a role for the displayed reactions. It could be imagined that slow addition of pre-mixed hydrazine and base are advantageous in cases where the hydrazine reacts with the alkyl halide in an S_N reaction.

5. Can the reaction be scaled up and down?

As demonstrated, the reaction works reliably on scales from 0.1 mmol to 0.1 mol. Due to its homogeneous nature, it is expected to easily scale beyond these limits. For a safety assessment, see scale-up section in the main manuscript.

6. Is the reaction temperature dependent?

Optimization and scope showed temperature dependence based on the activity of aryl halide, alkyl halide, and hydrazine, respectively. This also affects the reaction time.

7. <u>Why was the scope chosen? What are some limitations of the method?</u>

The scope was chosen as a representative example of the present method which replaces Zn reductant with homogenous aryl hydrazine. Thus, in main text Table 1 we highlight a few, key alkyl iodide coupling partners for the comparison.

Limitations of the reaction conditions include substrates which are prone to SN reactions and/or elimination reactions, such as primary and linear secondary alkyl halides. Tertiary halides showed conversion, but no coupling product was obtained (likely owing to steric issues with tertiary coupling and electronic stabilization effects of tertiary radicals discouraging capture by Ni) and only protodehalogenation was seen. Secondary bromides and chlorides proved difficult to activate under the present, mild conditions, even with the addition of either NaI or TBAI. Not yet examined are asymmetric alkyl halides or mixed halide systems. Further studies might target the optimization of these presently challenging coupling partners by addressing competing processes like homocoupling, protodehalogenation, elimination, or S_N reactions.

Abbreviations

ACN	acetonitrile
COD	1,5-cyclooctadiene
DCM	dichloromethane
DMA	di-methylacetamide
dme	dimethylether (ethylene glycol dimethyl ether ligand)
DMF	di-methylformamide
dtbbpy	4,4'-ditertbutyl-2,2'-bipyridine
HPLC	high performance liquid chromatography
MTBE	methyl tert-butyl ether
n.d.	not detected
NMP	N-methylpyrrolidone
PTFE	polytetrafluoroethylene
pTLC	preparative thin-layer chromatography
TBA	tetrabutylammonium ($n\text{Bu}_4^+$)
TCU	temperature control unit
THF	tetrahydrofuran
TMP	2,2,6,6-tetramethylpiperidine
TSR	temperature scanning ramp (calorimetry)
UPLC	ultra performance liquid chromatography

References

[1] H. Zhao, V. D. Cuomo, J. A. Rossi-Ashton, D. J. Procter, *Chem* **2024**, *10*, 1240–1251.

[2] G. Z. Wang, J. Jiang, X. S. Bu, J. J. Dai, J. Xu, Y. Fu, H. J. Xu, *Org Lett* **2015**, *17*, 3682–3685.

[3] Z. Zhang, B. Górski, D. Leonori, *J Am Chem Soc* **2022**, *144*, 1986–1992.

[4] H. Liu, Z. Liang, Q. Qian, K. Lin, *Synth Commun* **2014**, *44*, 2999–3007.

[5] J. M. E. Hughes, P. S. Fier, *Org Lett* **2019**, *21*, 5650–5654.

[6] A. Herath, V. Molteni, S. Pan, J. Loren, *Org Lett* **2018**, *20*, 7429–7432.

[7] D. F. Fernández, M. González-Esguevillas, S. Keess, F. Schäfer, J. Mohr, A. Shavnya, T. Knauber, D. C. Blakemore, D. W. C. MacMillan, *Org Lett* **2024**, *26*, 2702–2707.

[8] D. M. Allwood, D. C. Blakemore, A. D. Brown, S. V. Ley, *Journal of Organic Chemistry* **2014**, *79*, 328–338.

[9] M. Hofmayer, J. Hammann, G. Cahiez, P. Knochel, *Synlett* **2018**, *29*, 65–70.

[10] D. Parmar, L. Henkel, J. Dib, M. Rueping, *Chemical Communications* **2015**, *51*, 2111–2113.

[11] M. A. J. Dubois, J. J. Rojas, A. J. Sterling, H. C. Broderick, M. A. Smith, A. J. P. White, P. W. Miller, C. Choi, J. J. Mousseau, F. Duarte, et al., *Journal of Organic Chemistry* **2023**, *88*, 6476–6488.

[12] M. Nagase, Y. Kuninobu, M. Kanai, *J Am Chem Soc* **2016**, *138*, 6103–6106.

[13] P. J. Moon, S. Yin, R. J. Lundgren, *J Am Chem Soc* **2016**, *138*, 13826–13829.

[14] D. A. Cagan, D. Bim, B. Silva, N. P. Kazmierczak, B. J. McNicholas, R. G. Hadt, *J Am Chem Soc* **2022**, *144*, 6516–6531.

[15] D. A. Cagan, D. Bím, N. P. Kazmierczak, R. G. Hadt, *ACS Catalysis* **2024**, *14*, 9055–9076.

[16] D. A. Cagan, Á. Péter, B. P. Vokits, M. Chan, P. S. Baran, D. G. Blackmond, **2025**, DOI 10.26434/CHEMRXIV-2025-8HGHL.

Spectral Data

^{1}H NMR of Compound 4 (600 MHz, $CDCl_3$):

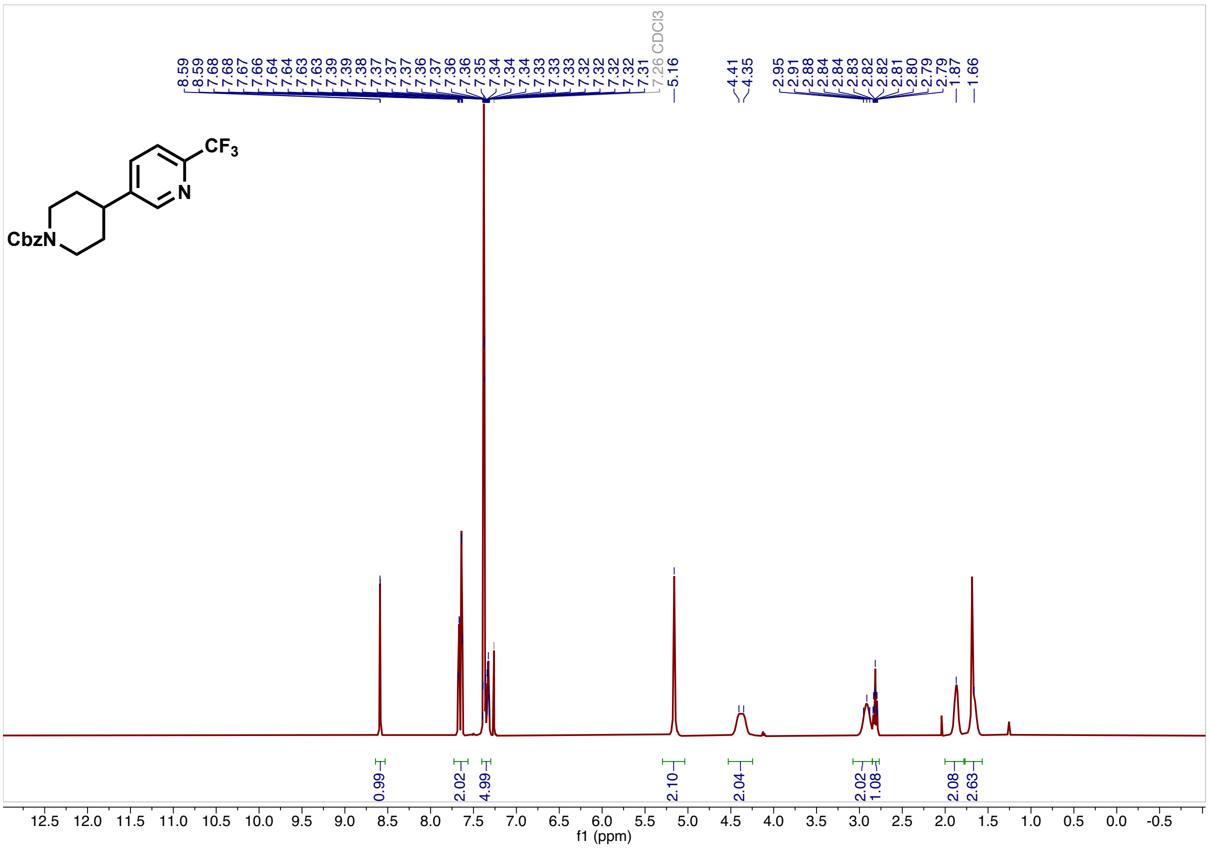

^{13}C NMR of Compound 4 (151 MHz, $CDCl_3$):

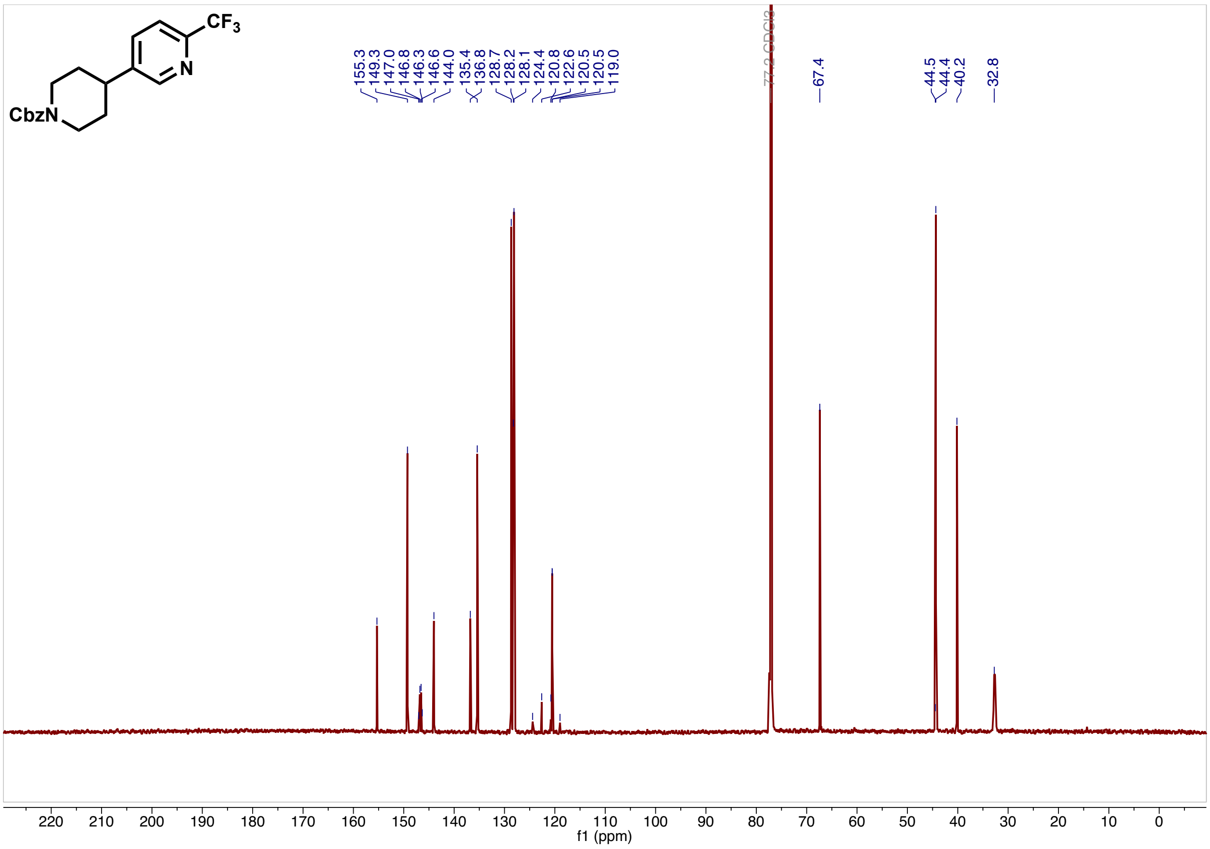

^{19}F NMR of Compound 4 (376 MHz, $CDCl_3$):

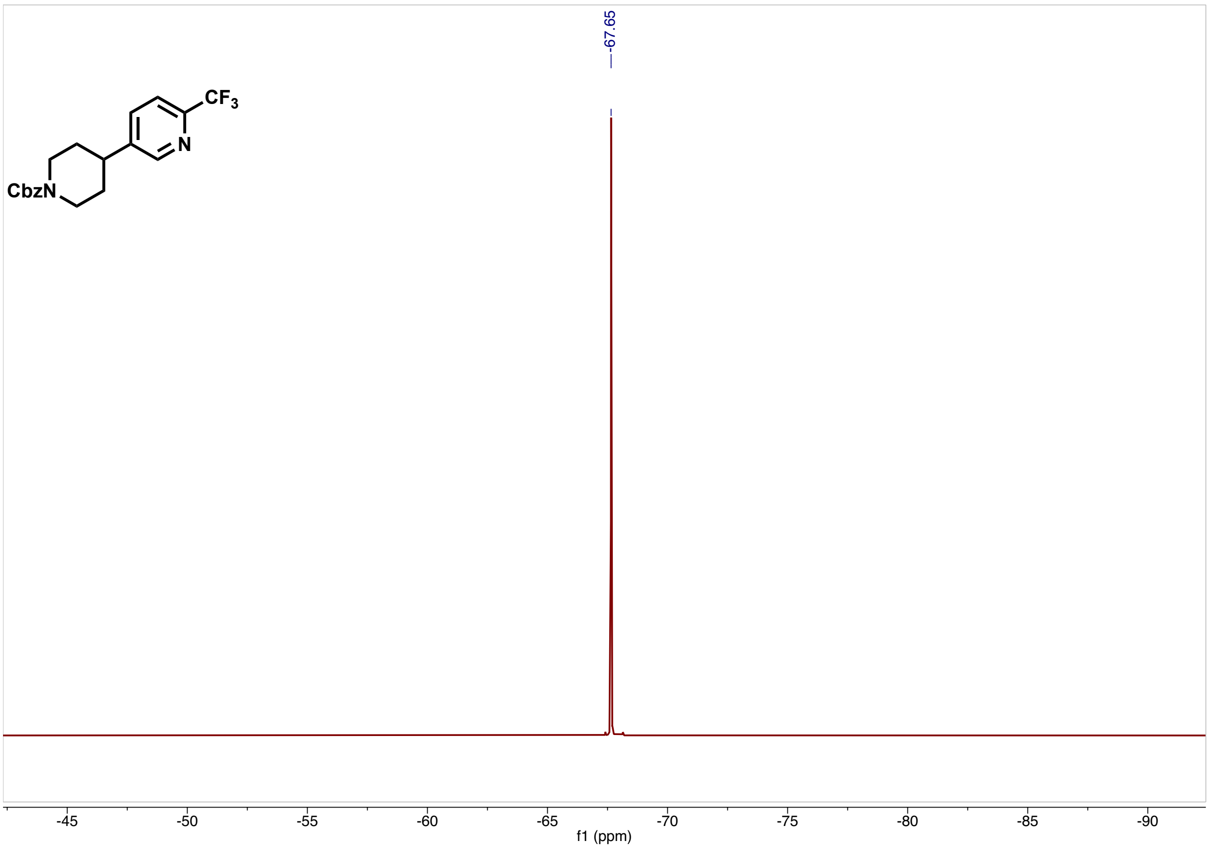

^{1}H NMR of Compound 5 (600 MHz, $CDCl_3$):

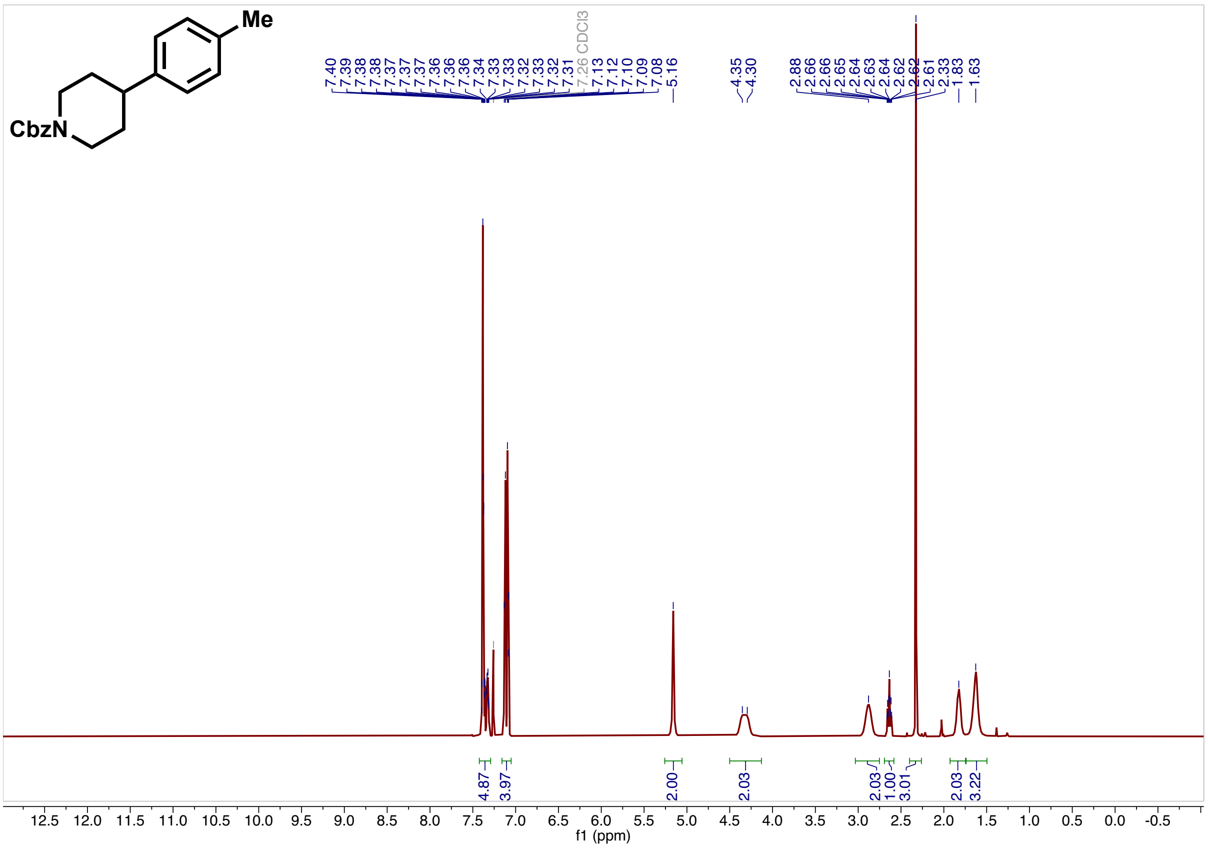

^{13}C NMR of Compound 5 (151 MHz, $CDCl_3$):

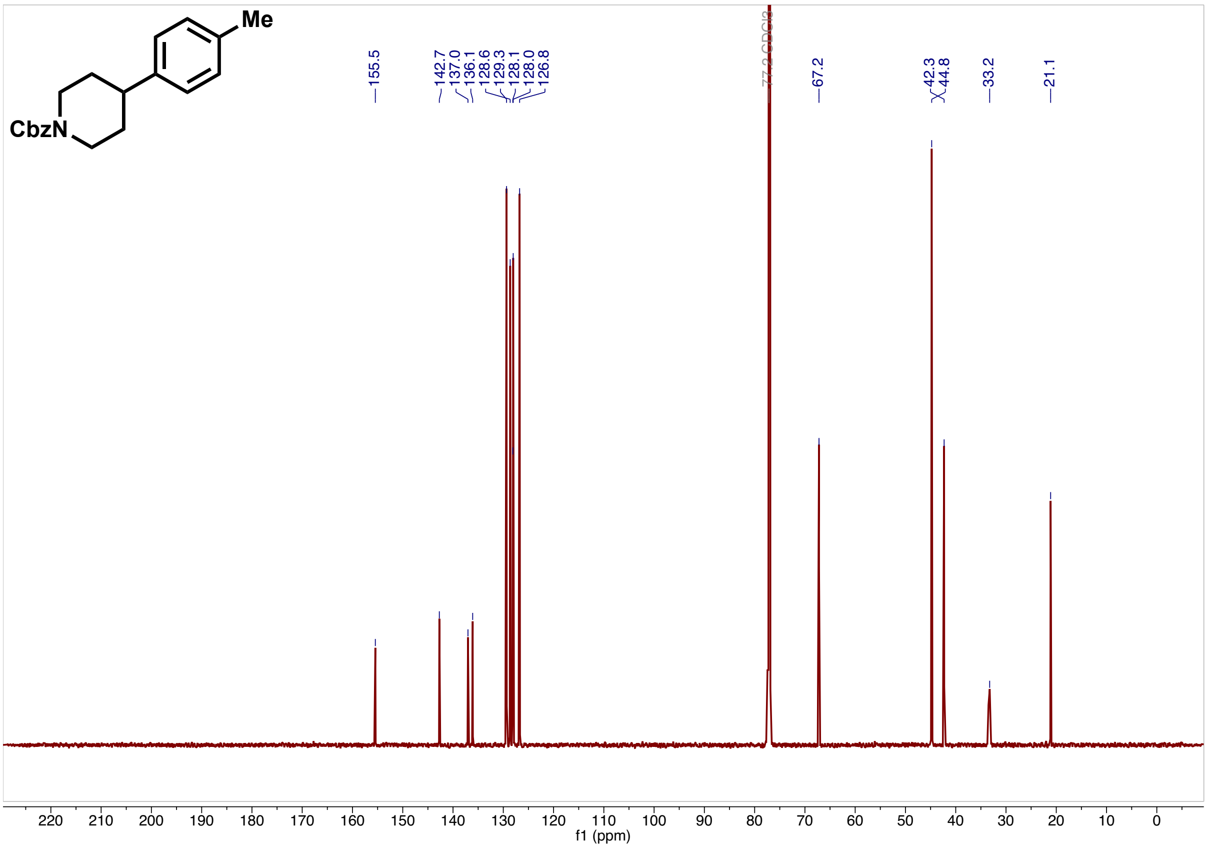

^{1}H NMR of Compound 6 (600 MHz, $CDCl_3$):

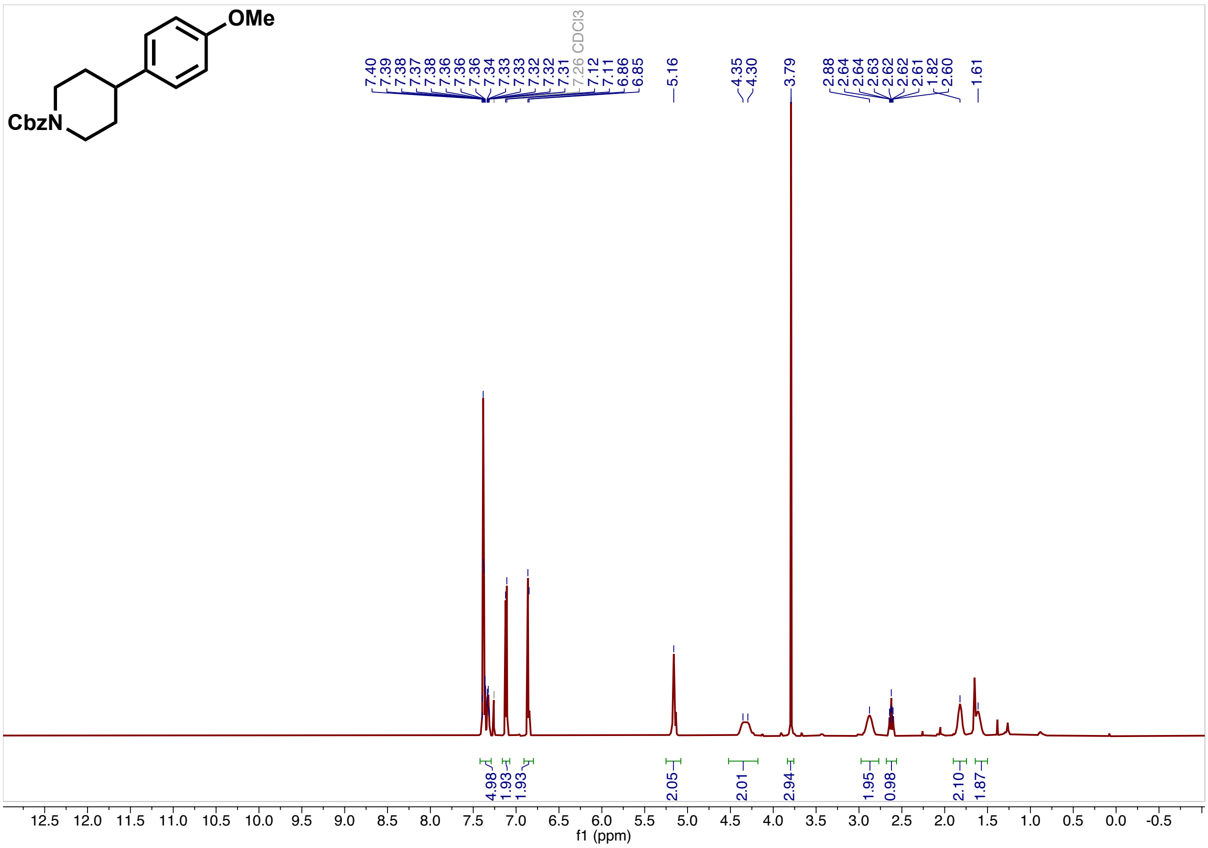

^{13}C NMR of Compound 6 (151 MHz, $CDCl_3$):

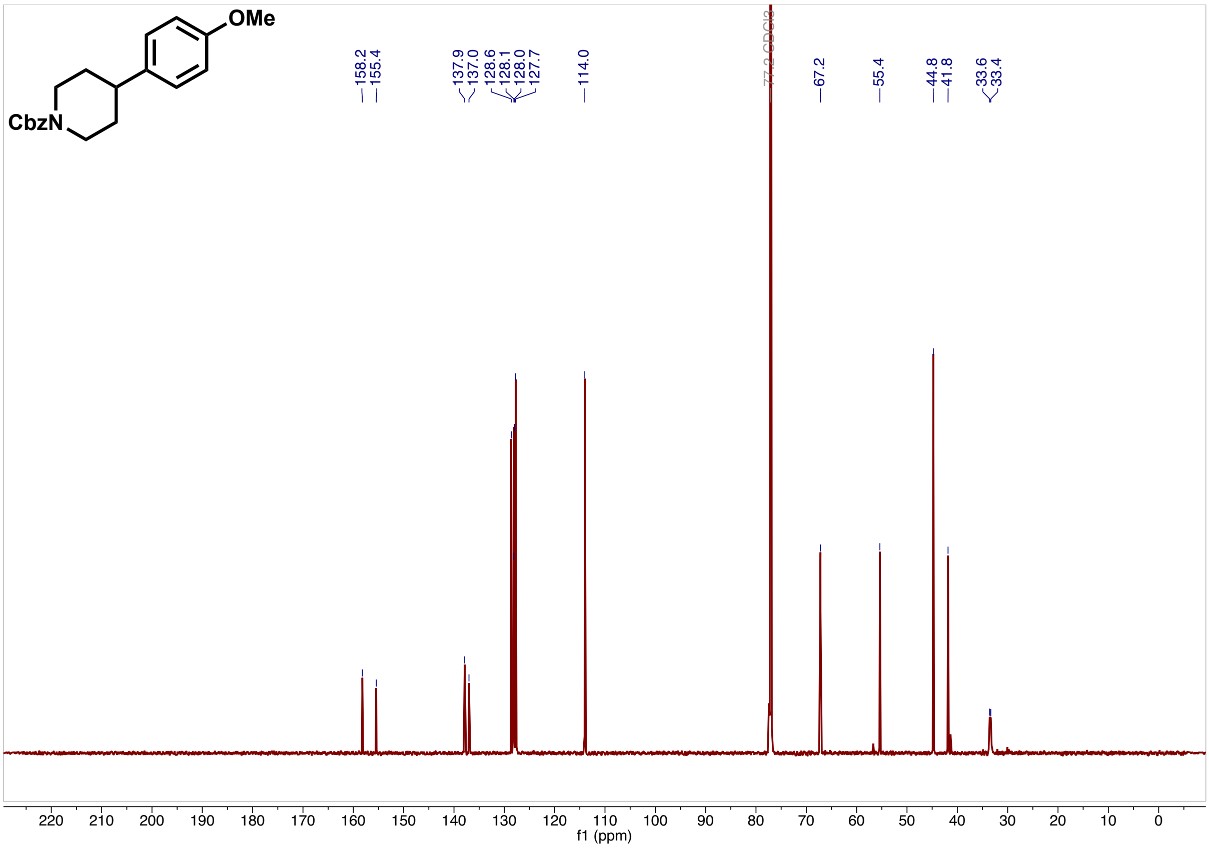

^{1}H NMR of Compound 7 (600 MHz, $CDCl_3$):

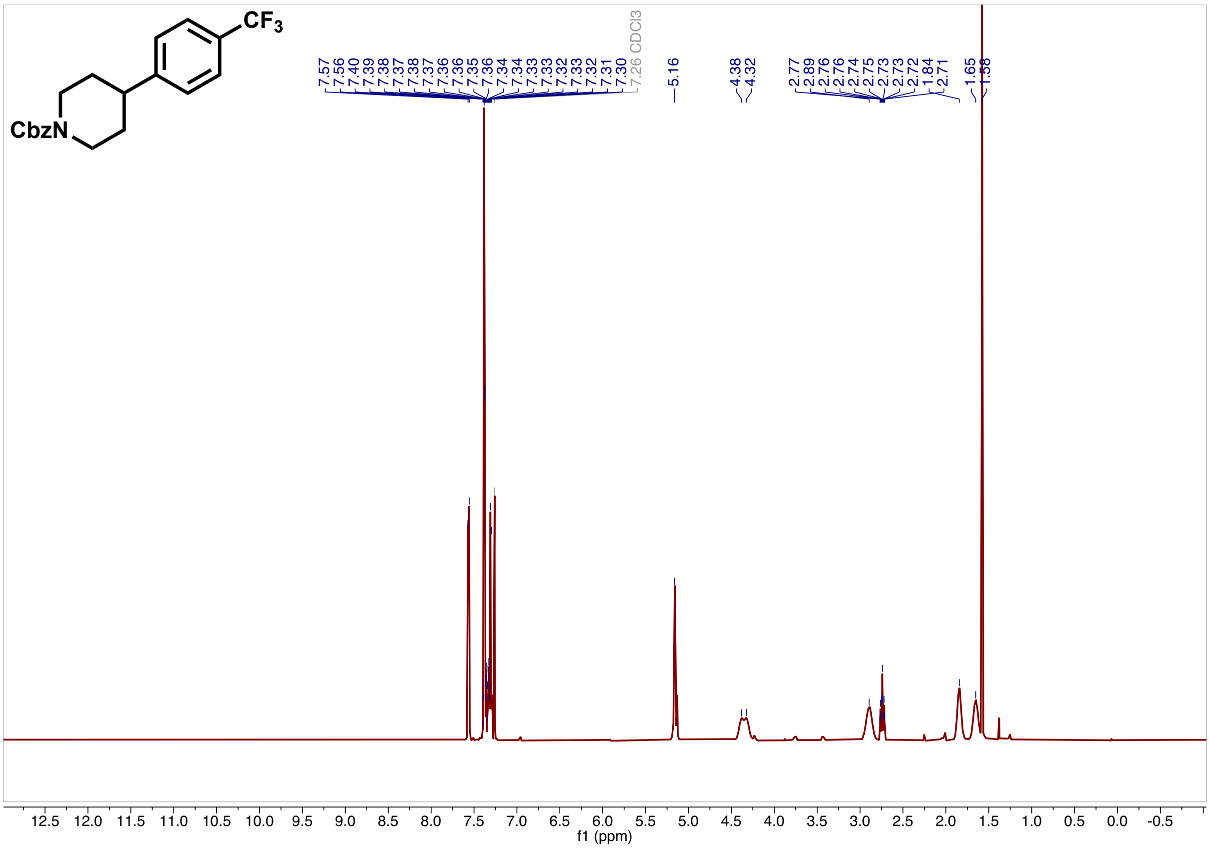

^{13}C NMR of Compound 7 (151 MHz, $CDCl_3$):

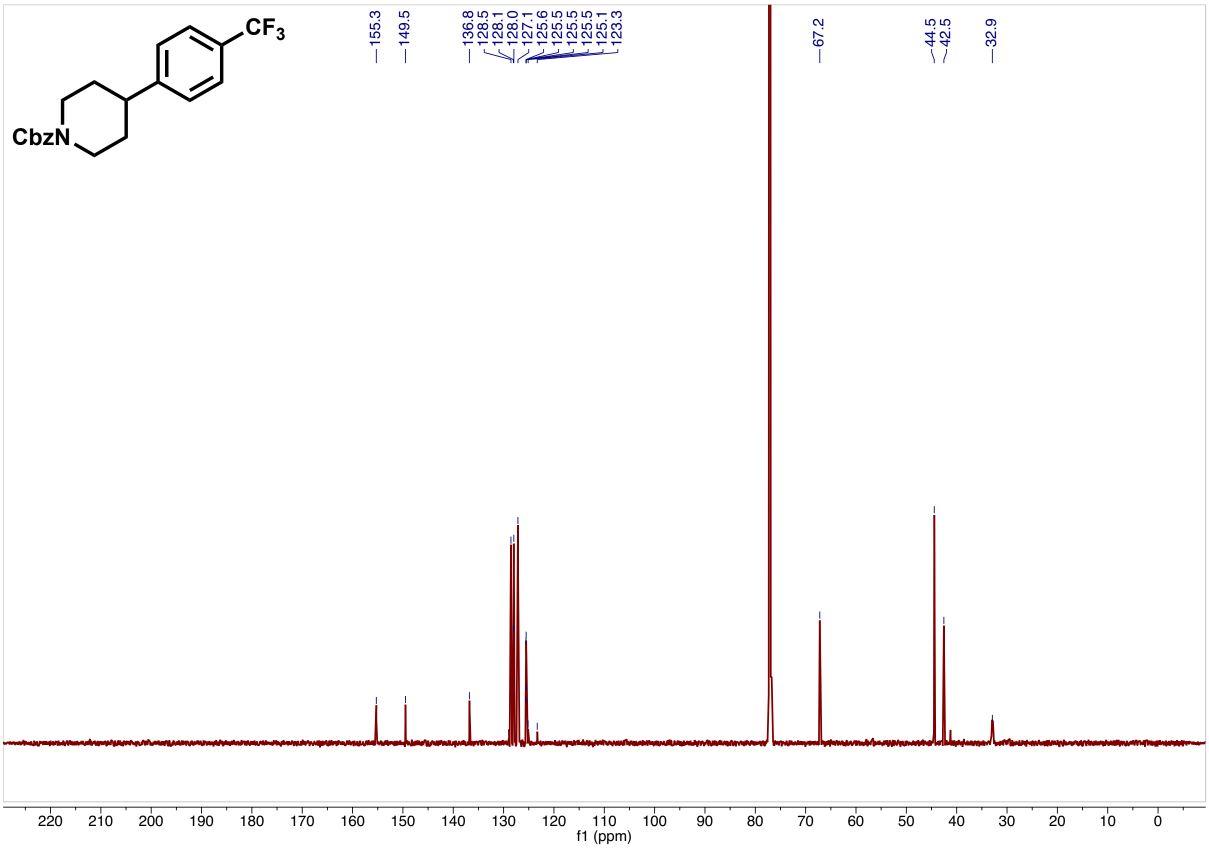

^{19}F NMR of Compound 7 (376 MHz, $CDCl_3$):

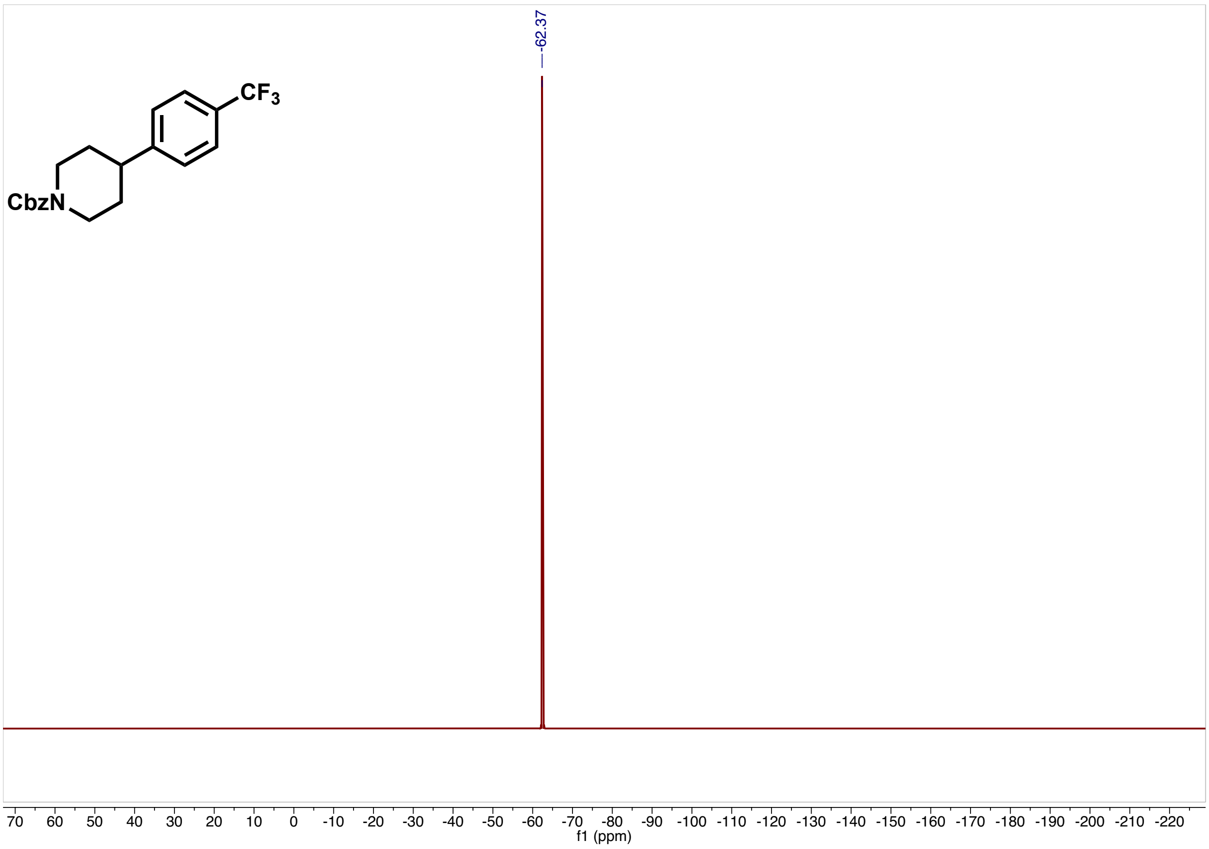

^1H NMR of Compound 8 (600 MHz, $CDCl_3$):

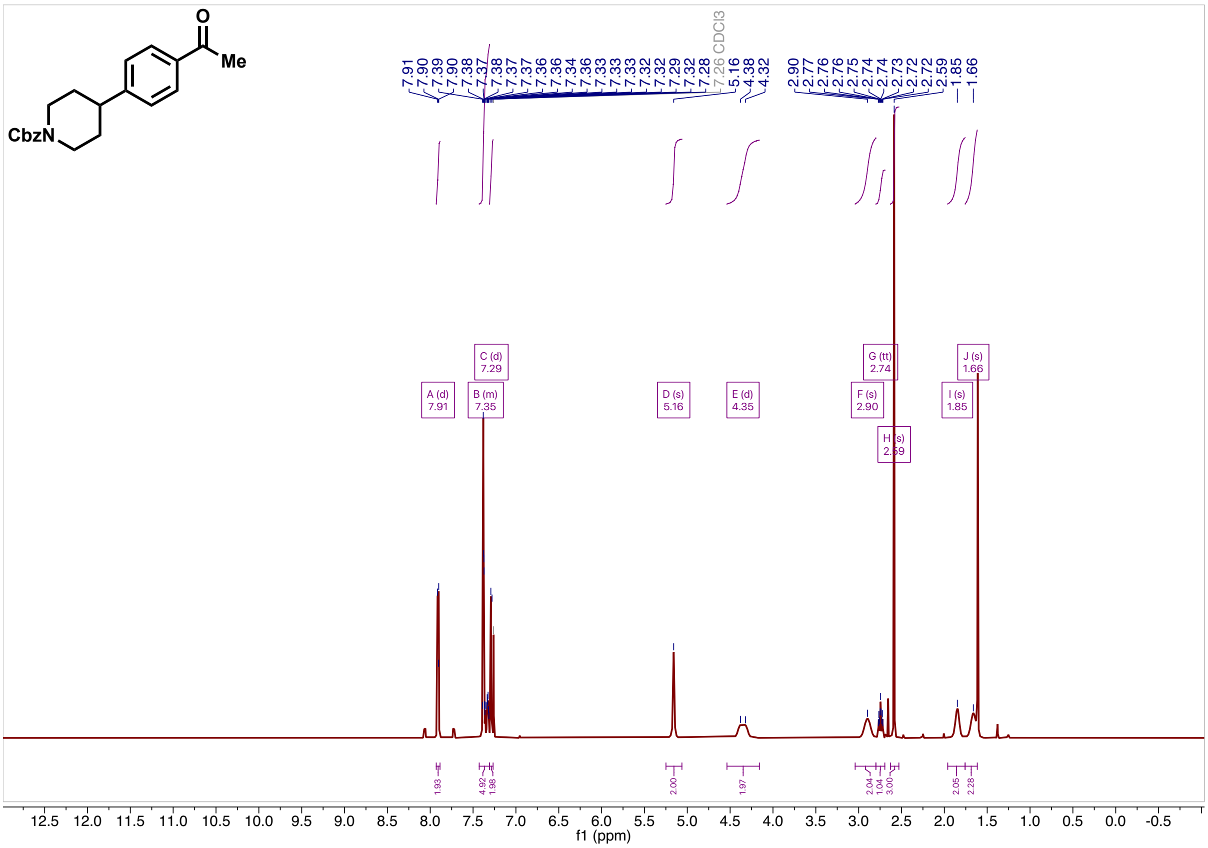

^{13}C NMR of Compound 8 (151 MHz, $CDCl_3$):

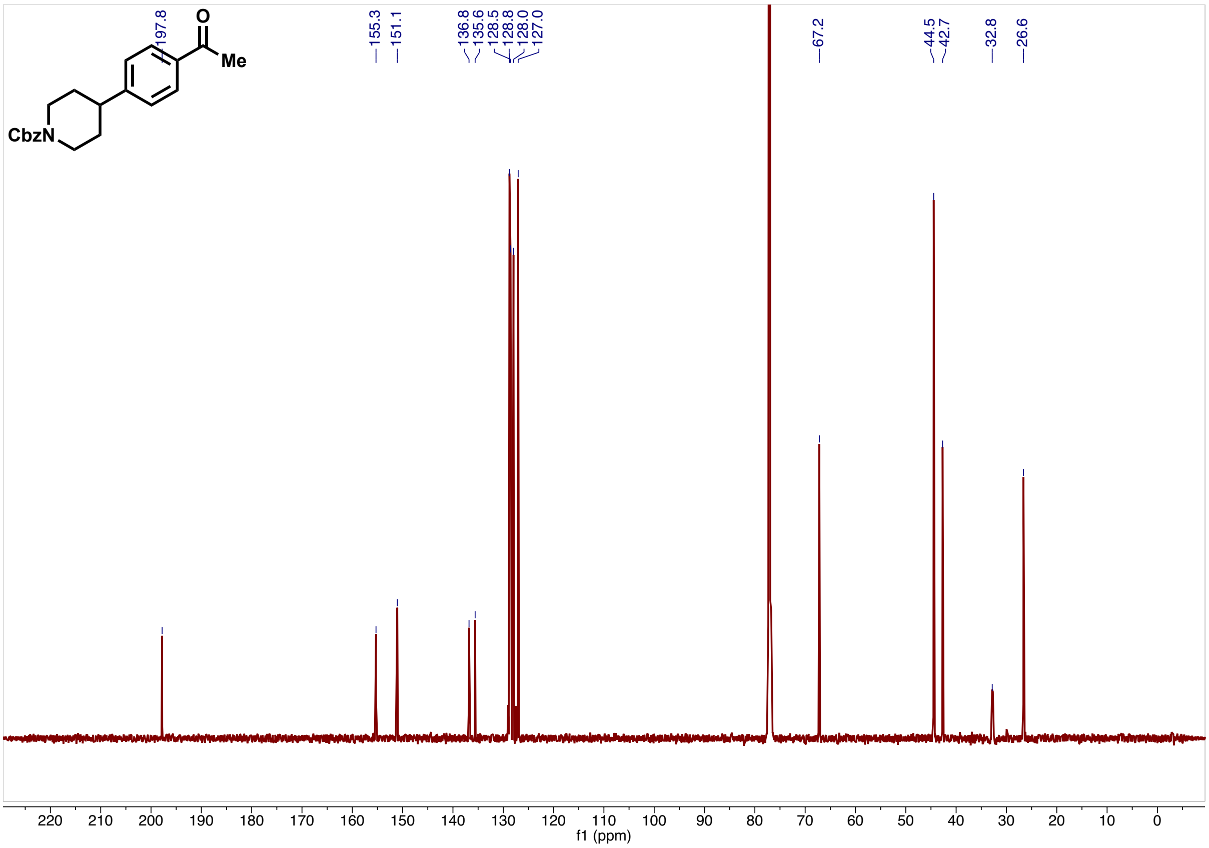

^{1}H NMR of Compound 9 (600 MHz, $CDCl_3$):

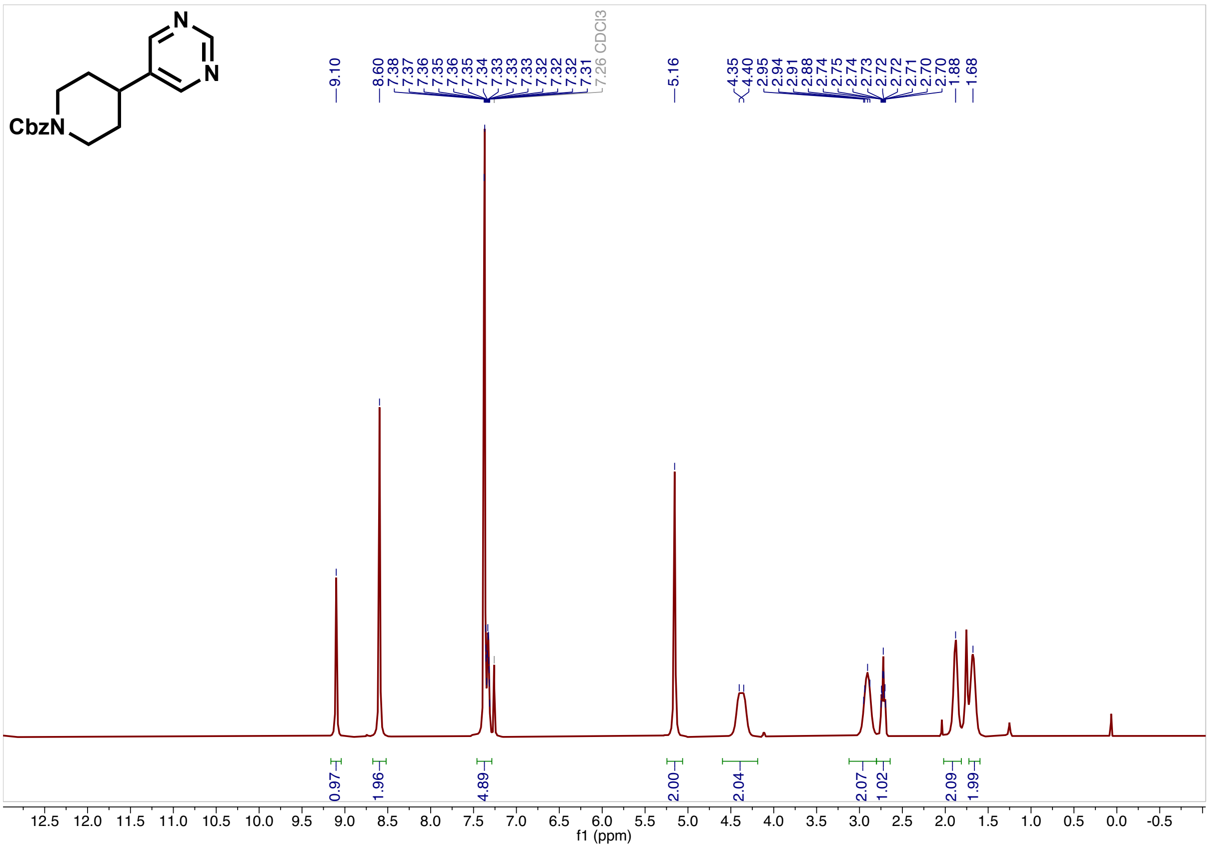

^{13}C NMR of Compound 9 (151 MHz, $CDCl_3$):

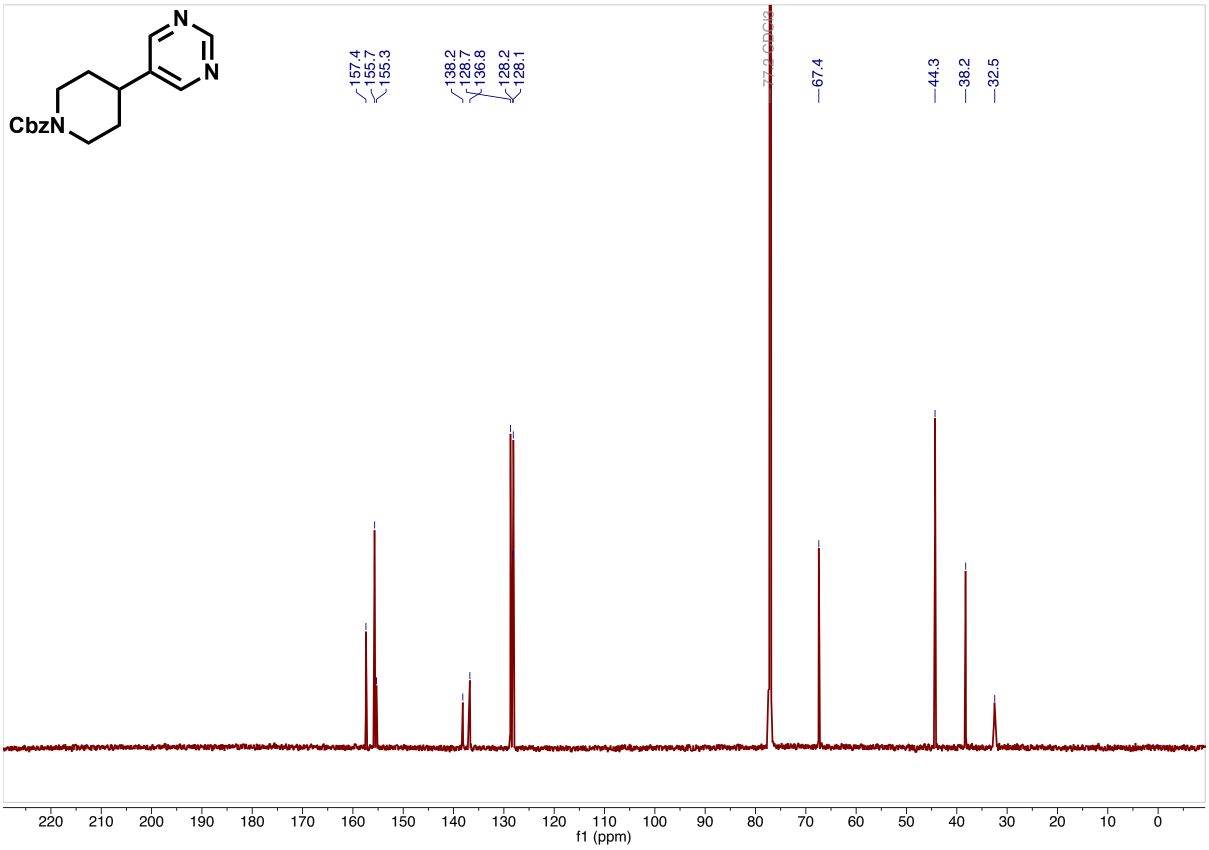

^{1}H NMR of Compound 10 (600 MHz, $CDCl_3$):

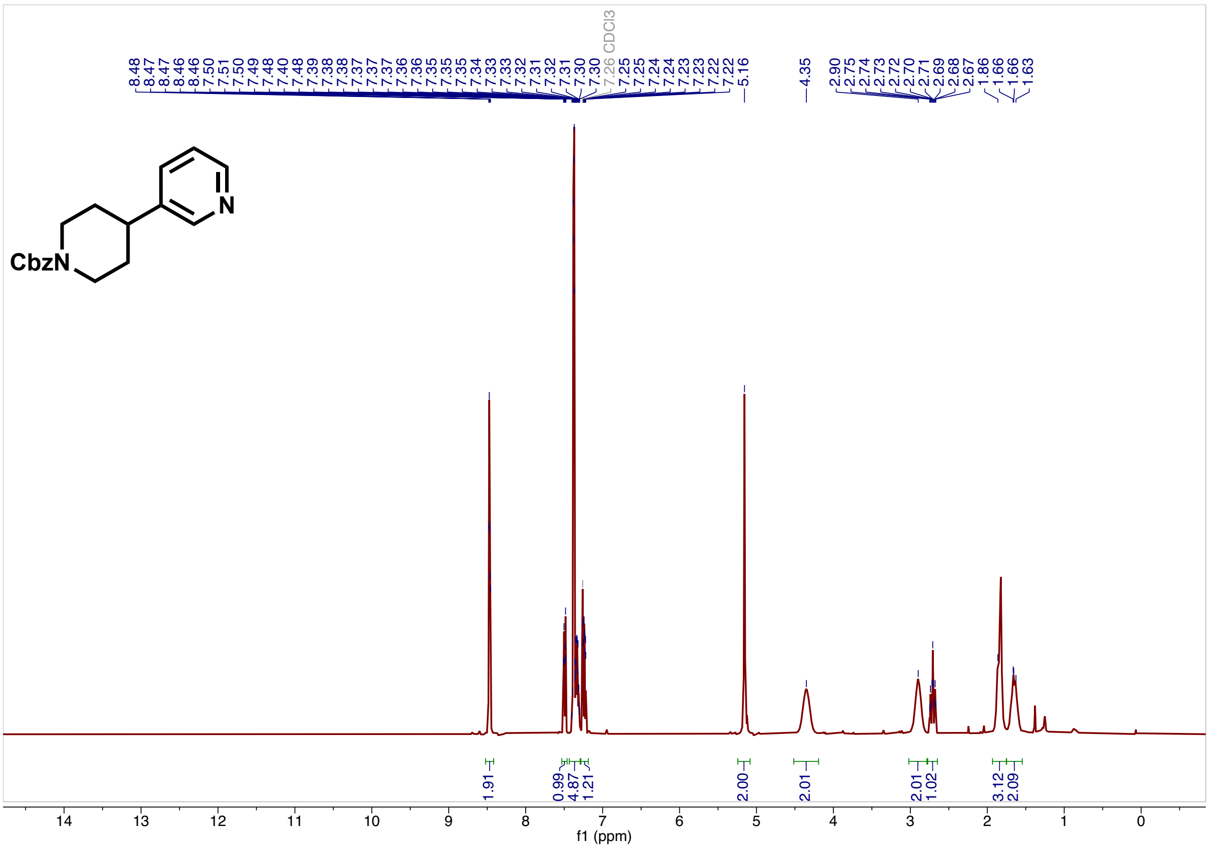

^{13}C NMR of Compound 10 (151 MHz, $CDCl_3$):

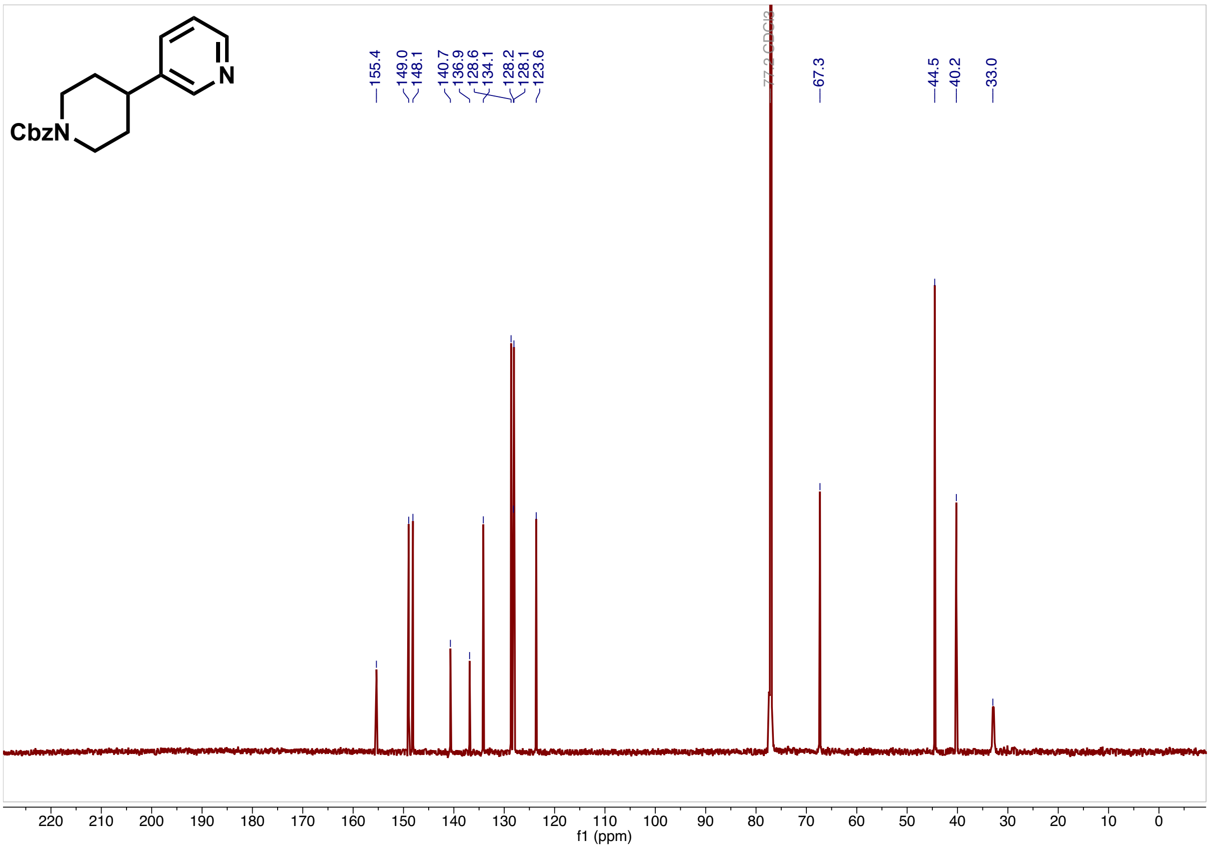

H NMR of Compound 11 (600 MHz, $CDCl_3$):

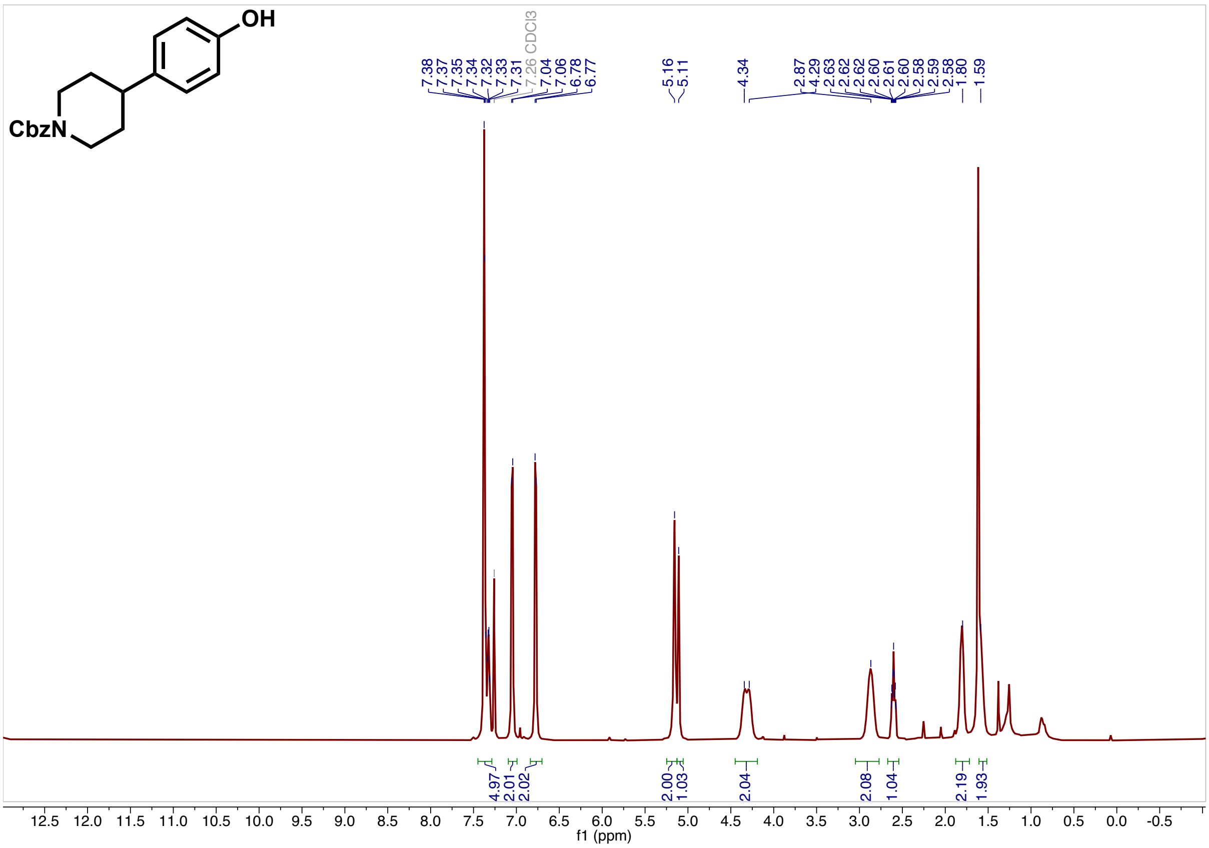

^{13}C NMR of Compound 11 (151 MHz, $CDCl_3$):

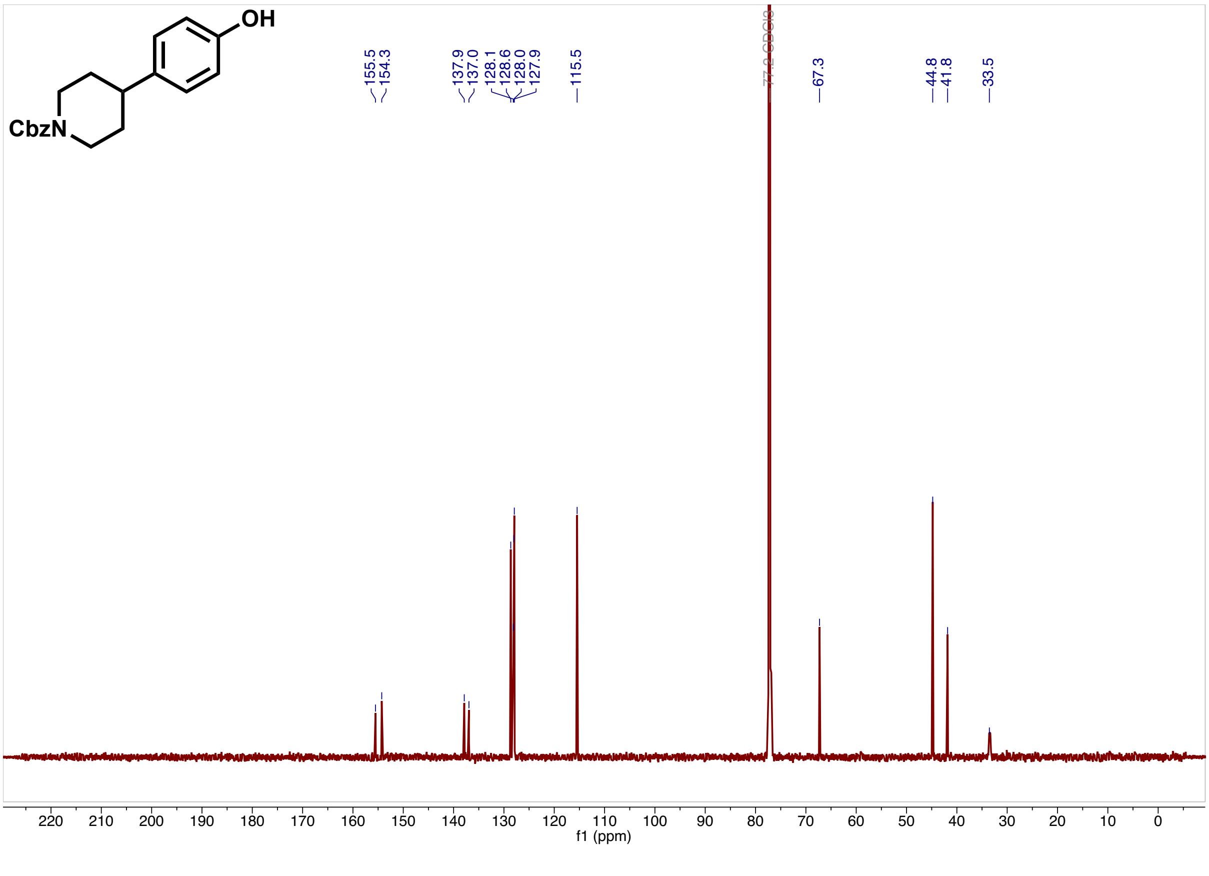

^{1}H NMR of Compound 12 (600 MHz, $CDCl_3$):

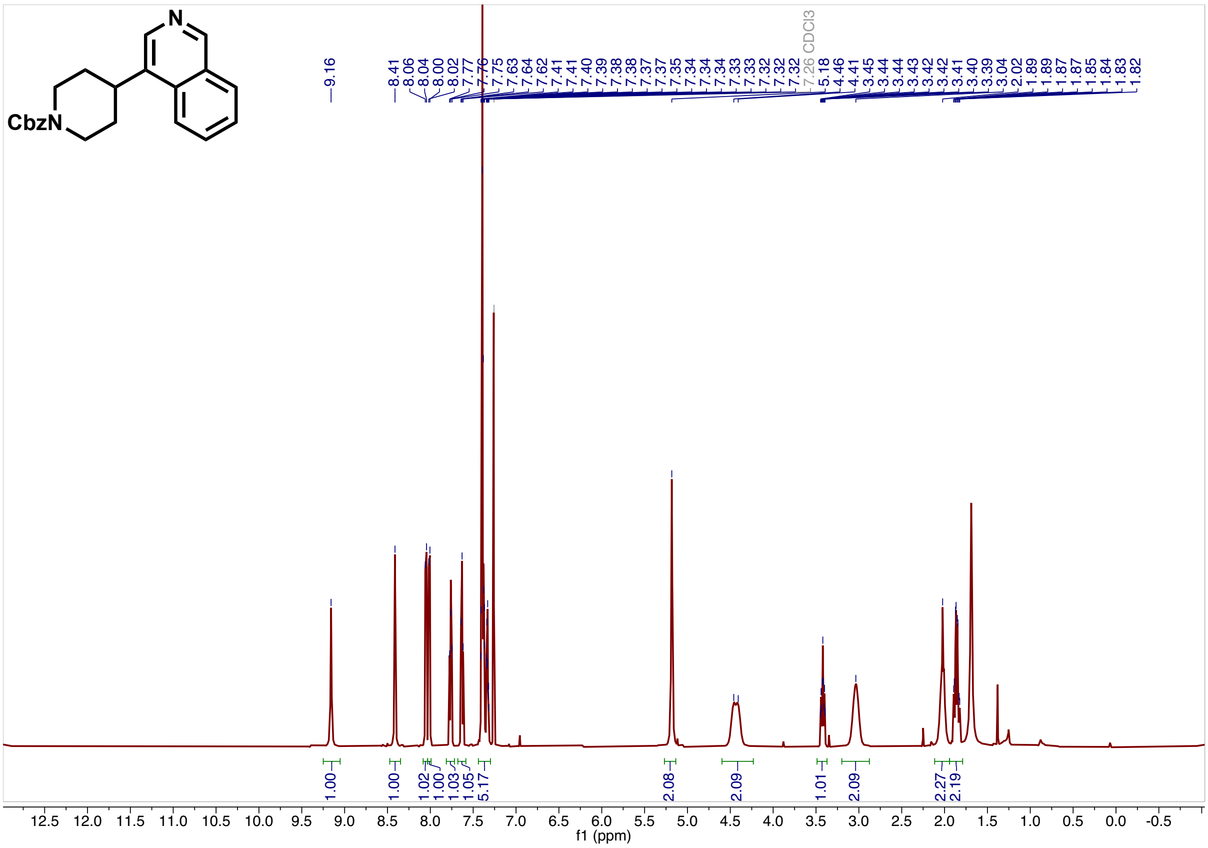

^{13}C NMR of Compound 12 (151 MHz, $CDCl_3$):

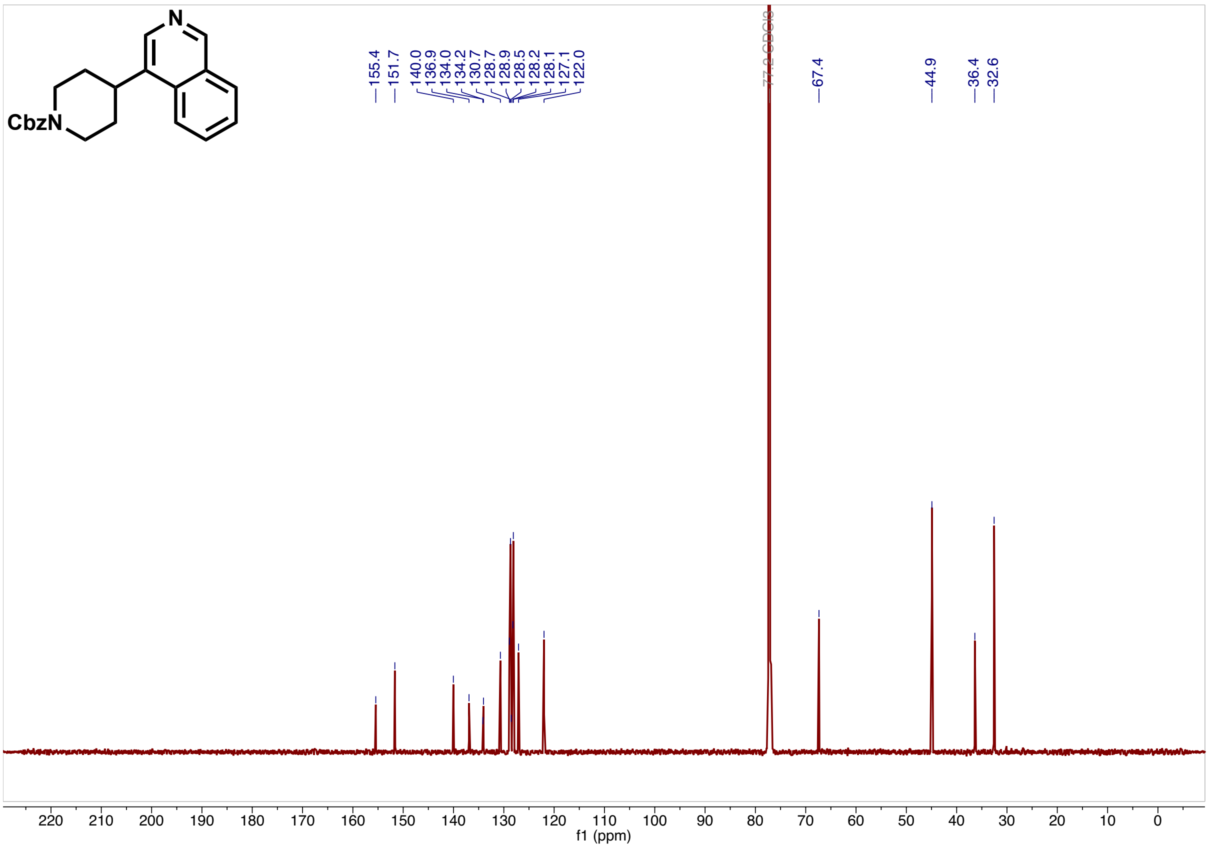

^{1}H NMR of Compound 13 (600 MHz, $CDCl_3$):

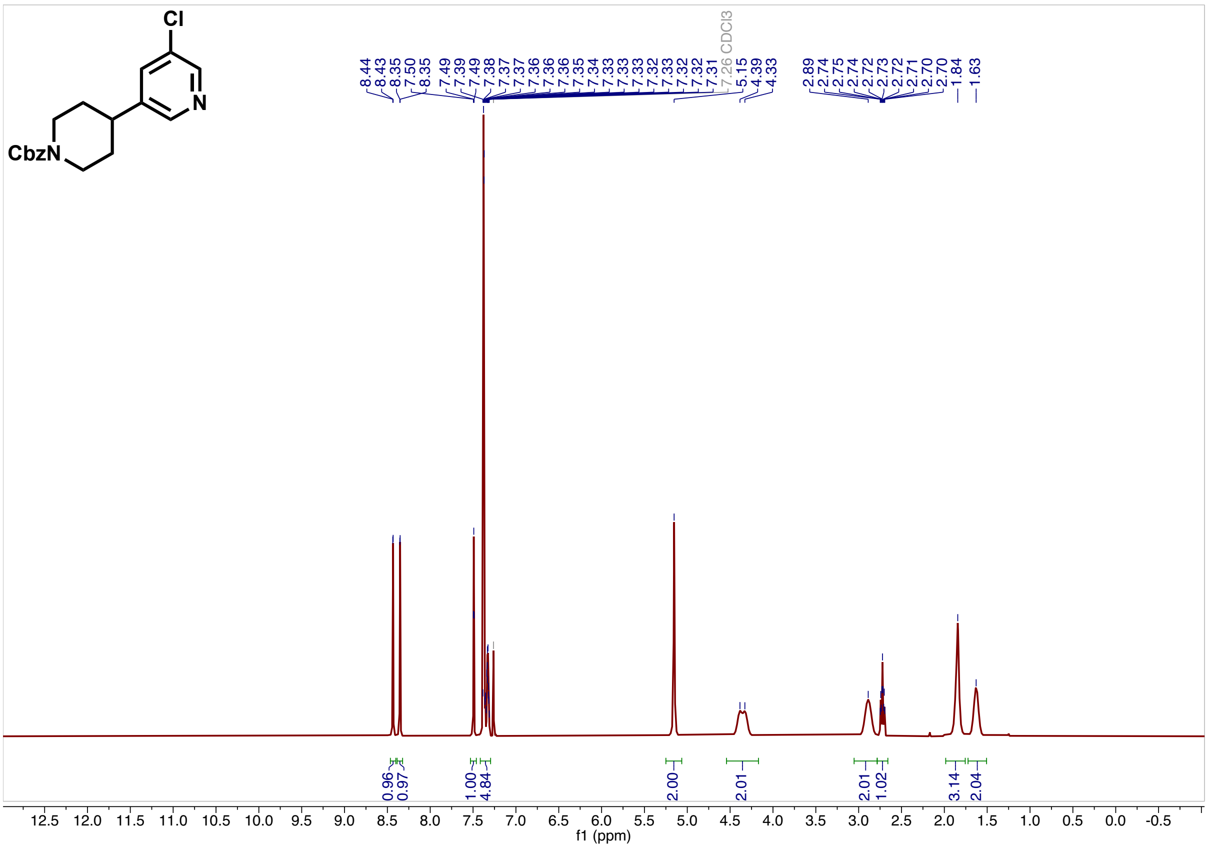

^{13}C NMR of Compound 13 (151 MHz, $CDCl_3$):

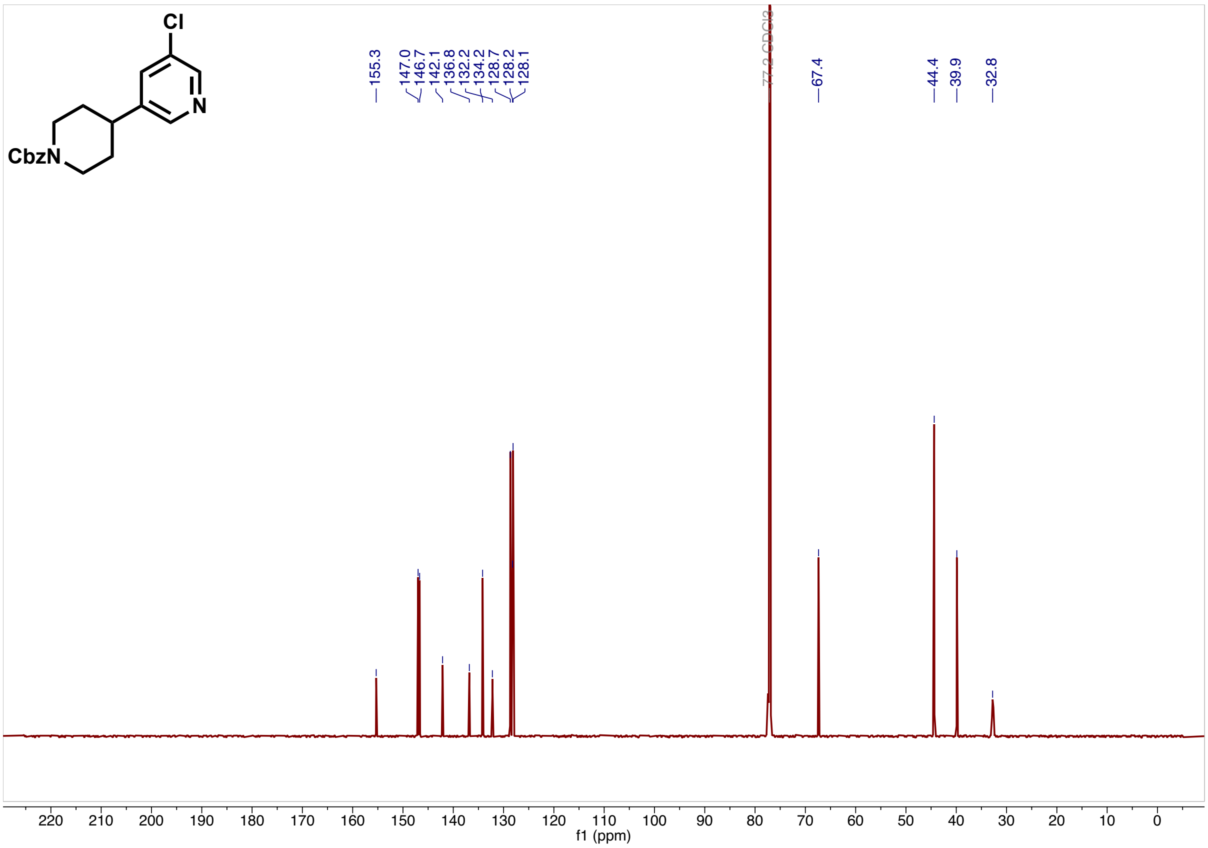

^{1}H NMR of Compound 14 (600 MHz, $CDCl_3$):

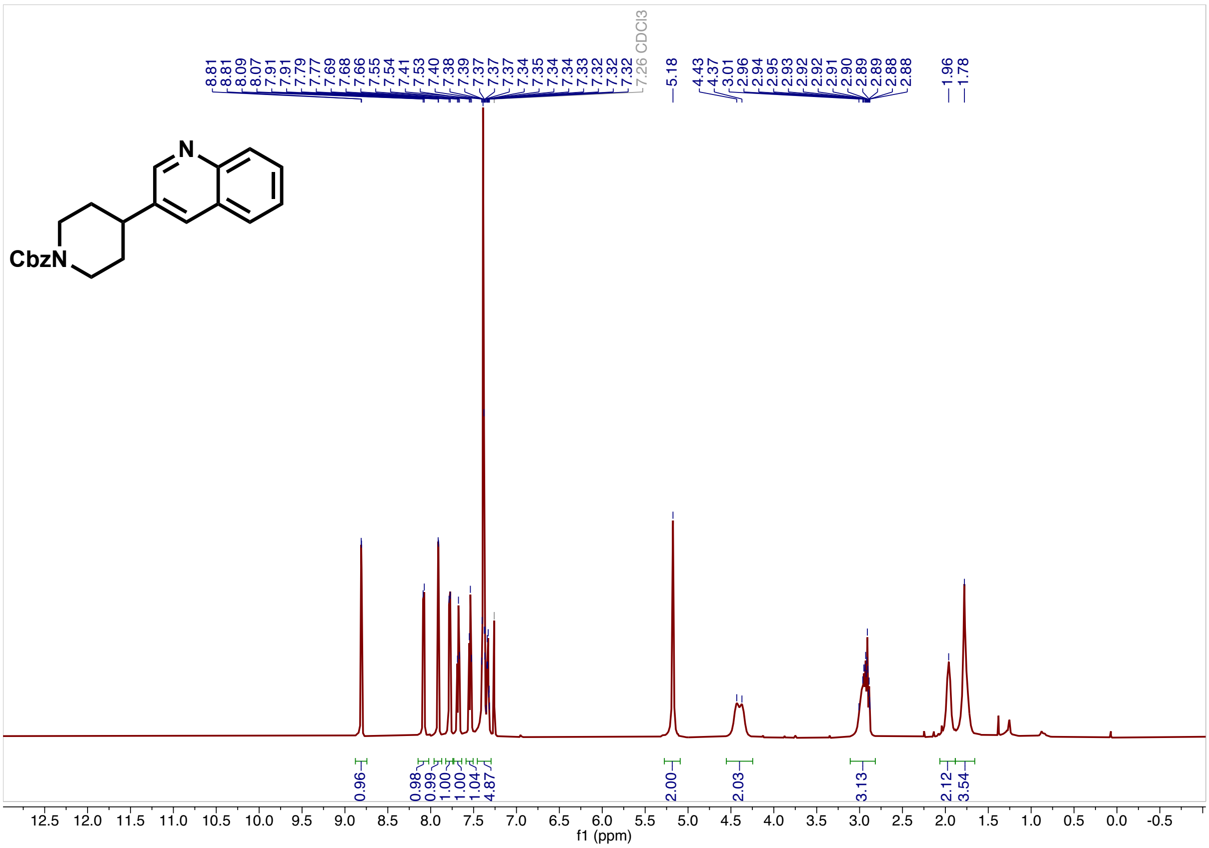

^{13}C NMR of Compound 14 (151 MHz, $CDCl_3$):

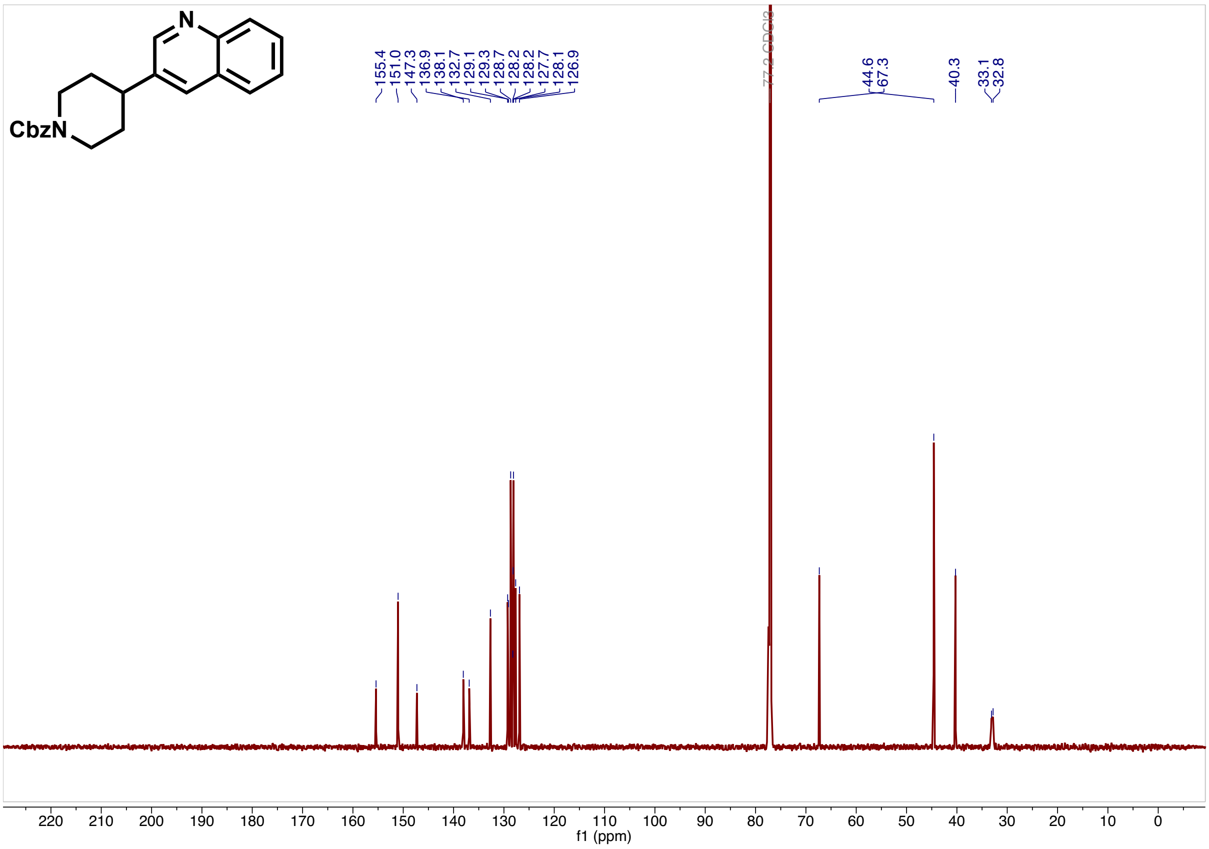

^{1}H NMR of Compound 16 (600 MHz, $CDCl_3$):

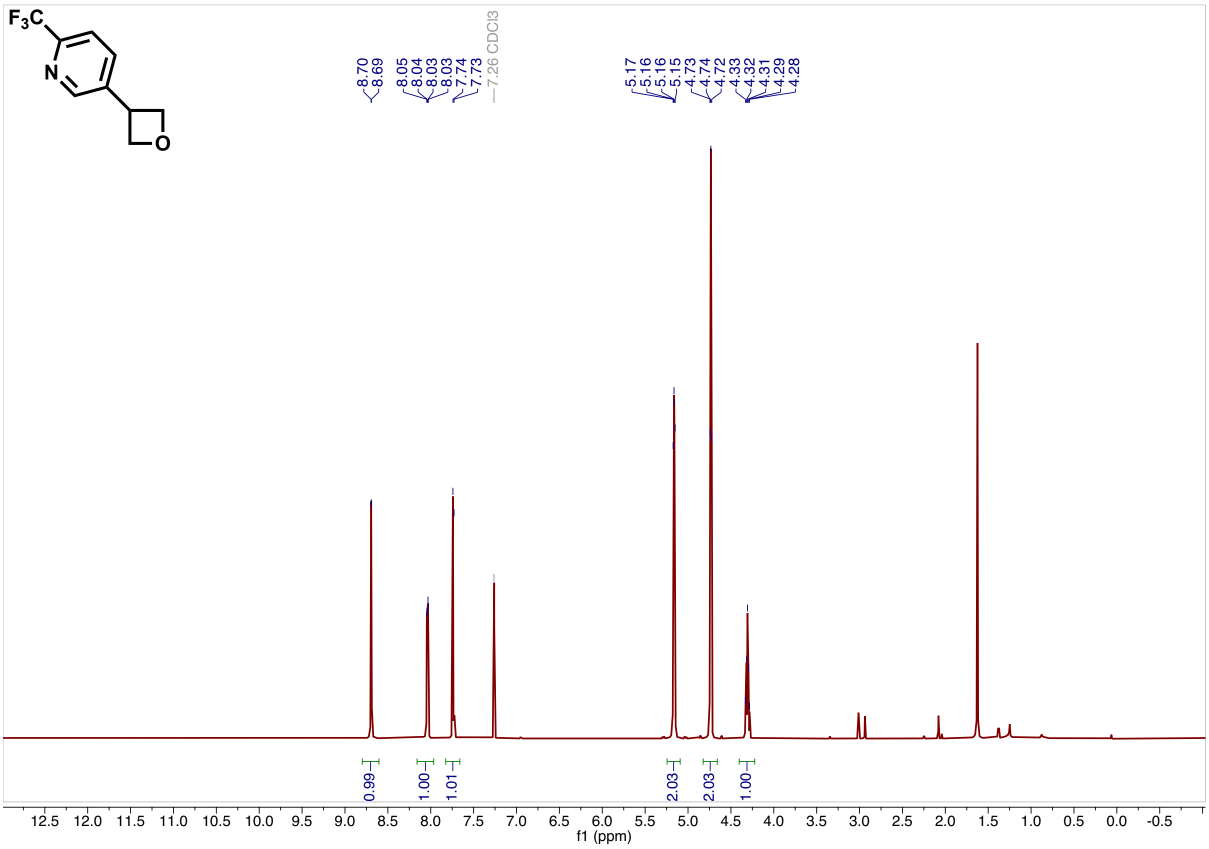

^{13}C NMR of Compound 16 (151 MHz, $CDCl_3$):

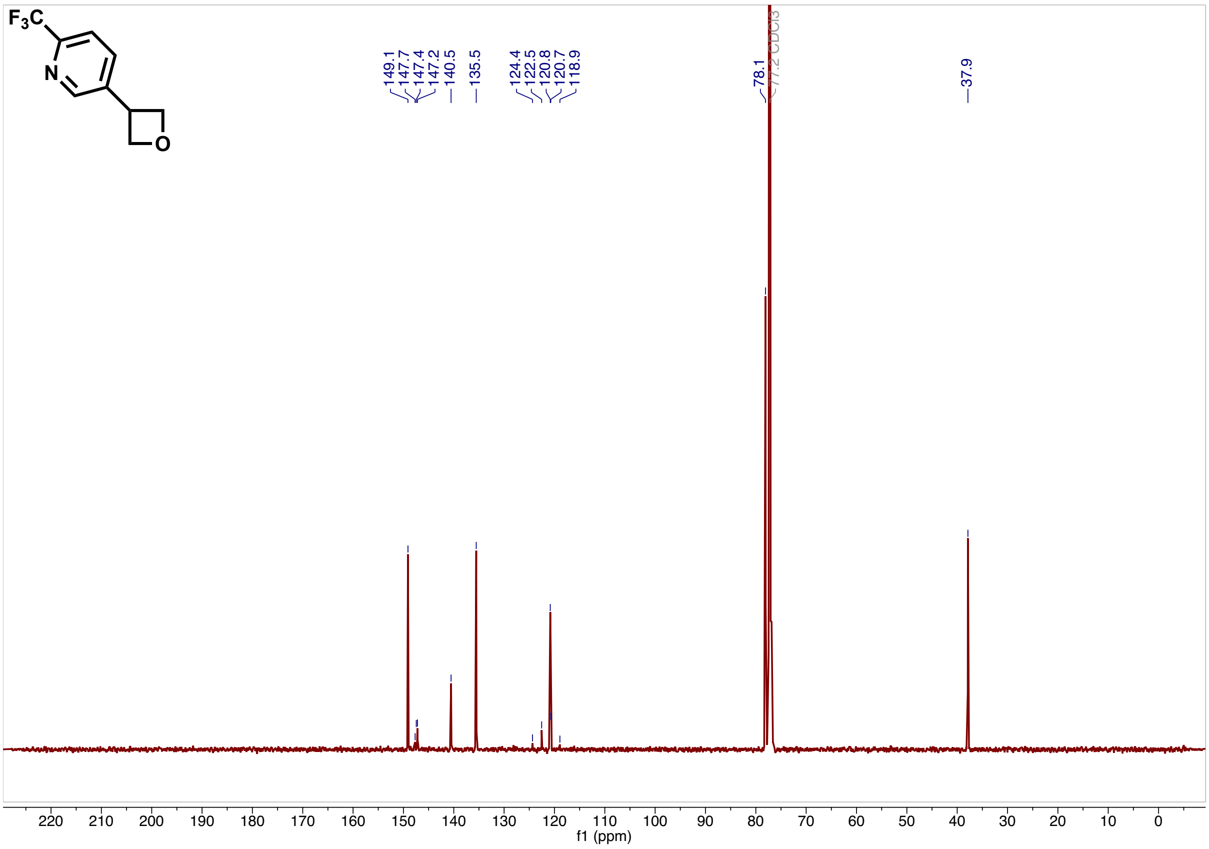

^{19}F NMR of Compound 16 (376 MHz, $CDCl_3$):

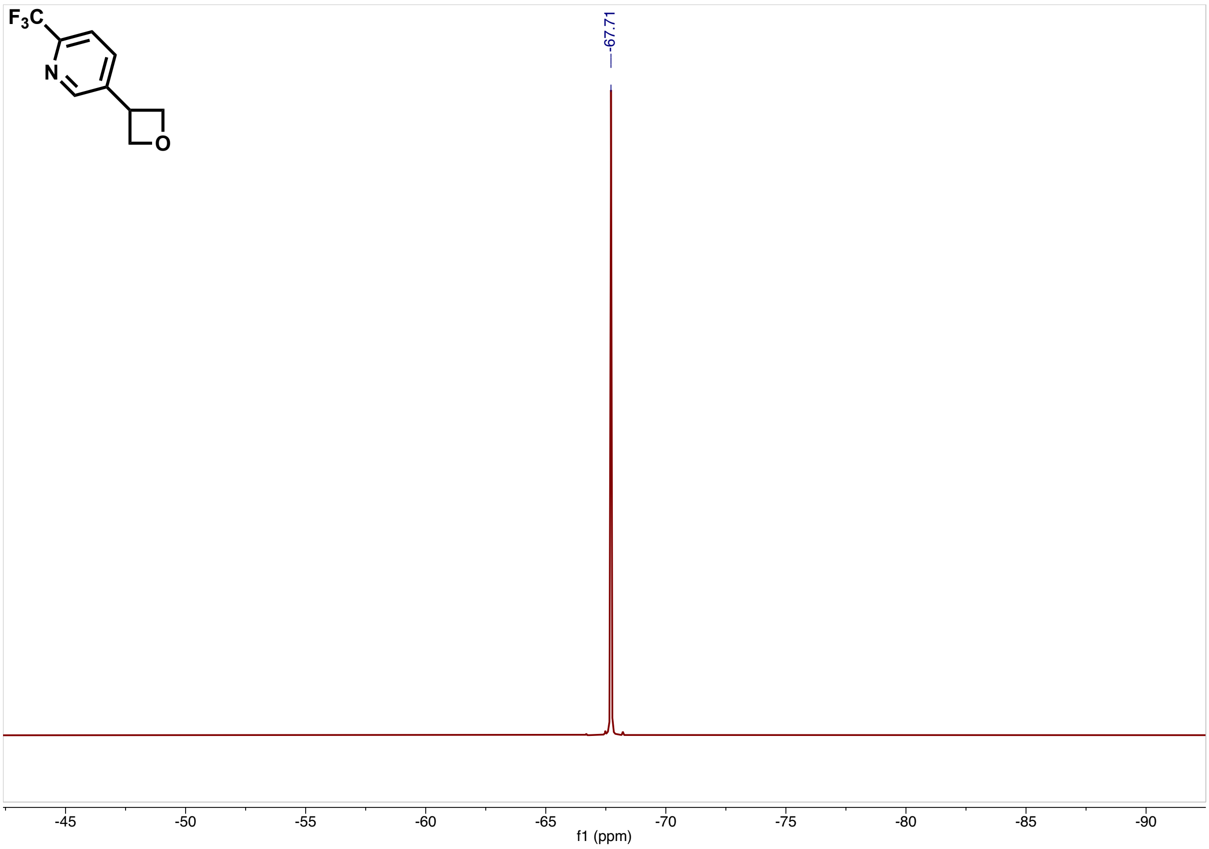

^{1}H NMR of Compound 17 (600 MHz, $CDCl_3$):

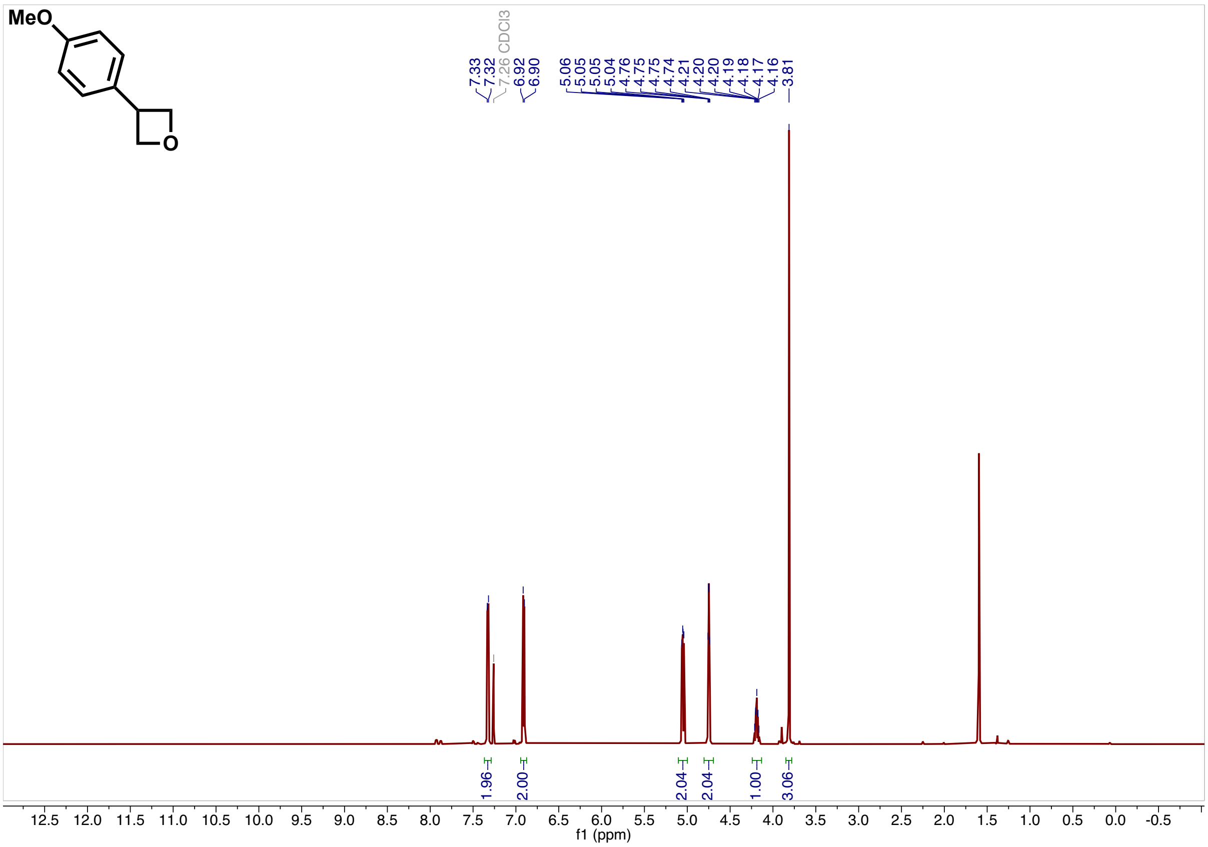

^{13}C NMR of Compound 17 (151 MHz, $CDCl_3$):

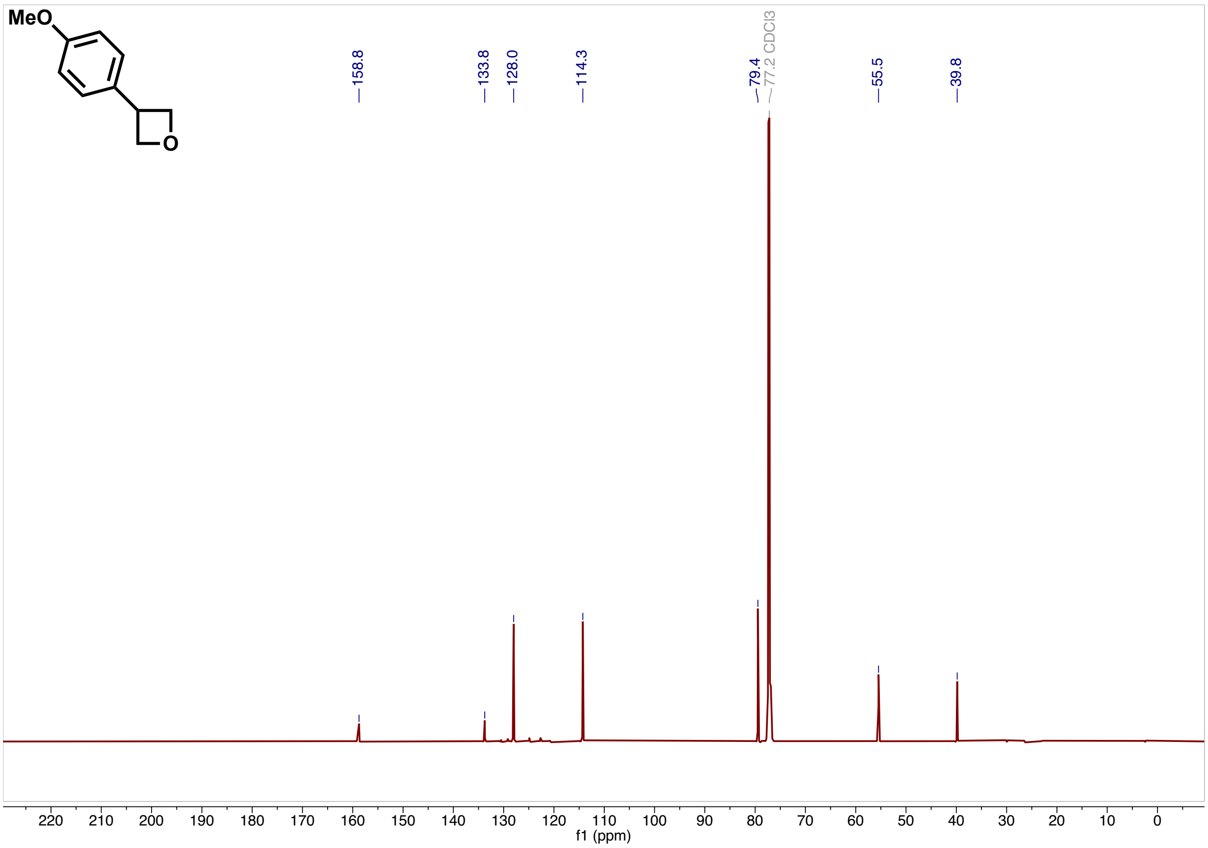

^{1}H NMR of Compound 18 (600 MHz, $CDCl_3$):

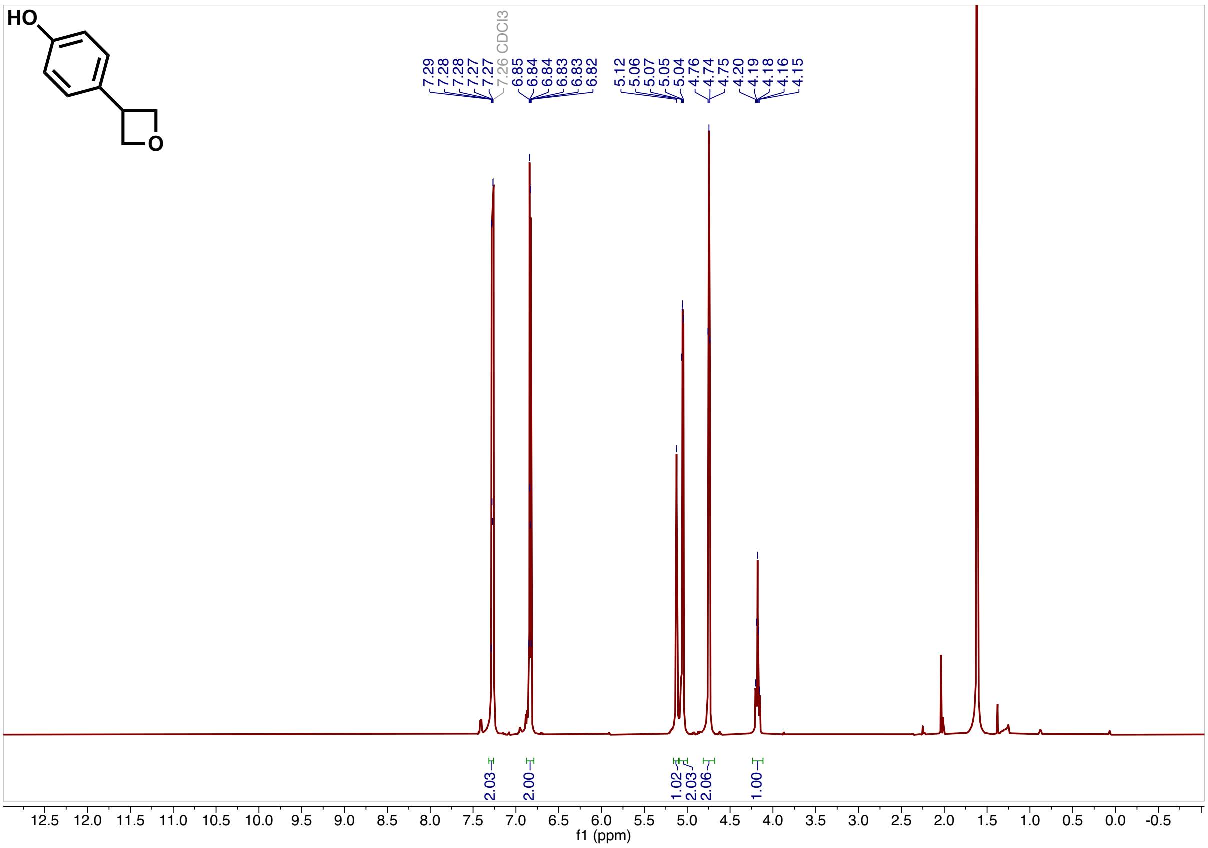

^{13}C NMR of Compound 18 (151 MHz, $CDCl_3$):

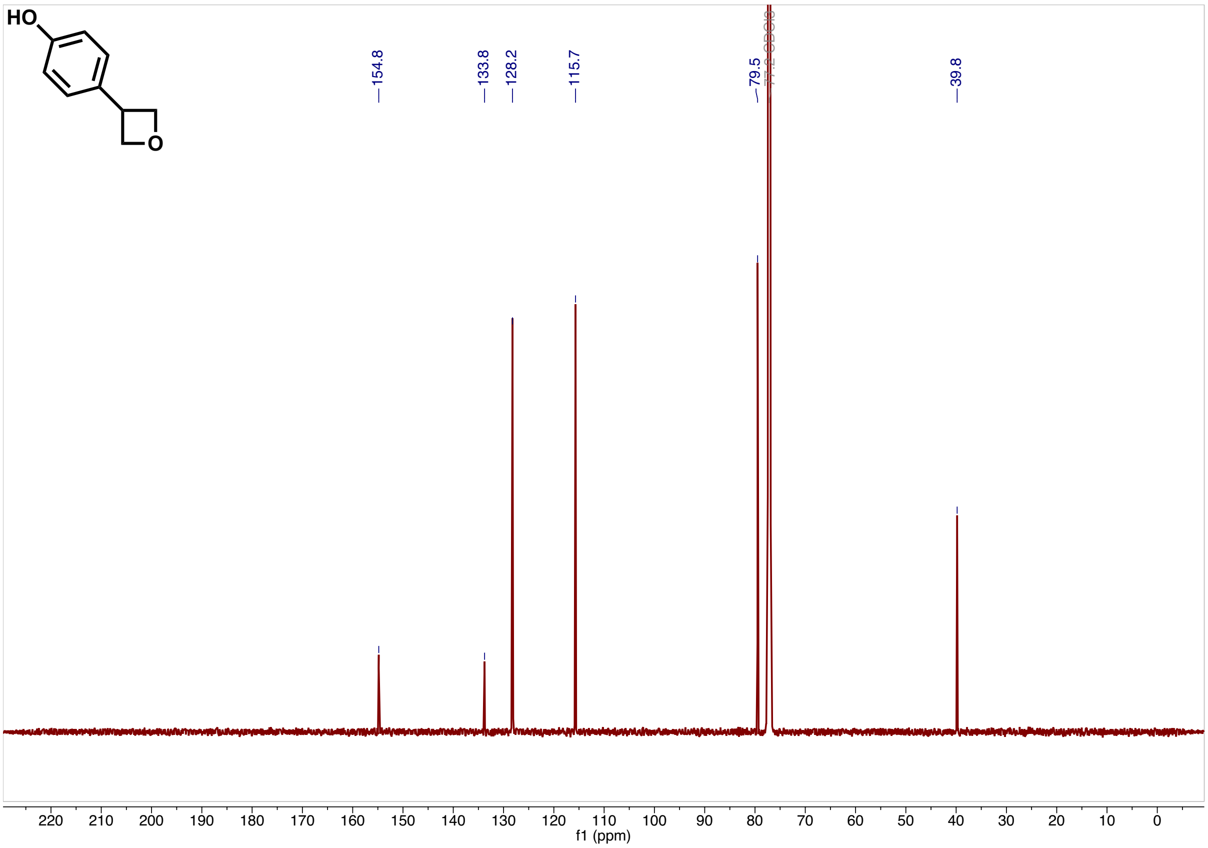

^{1}H NMR of Compound 19 (600 MHz, $CDCl_3$):

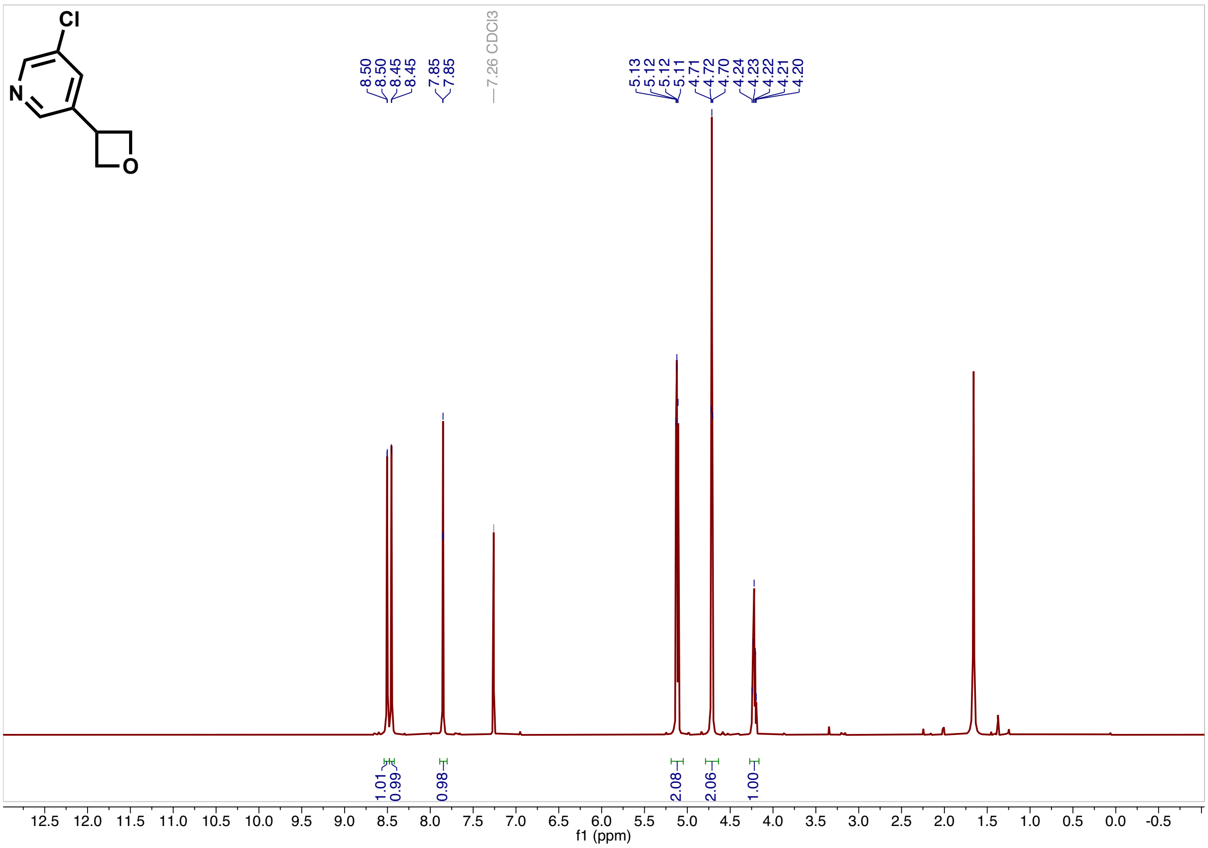

^{13}C NMR of Compound 19 (151 MHz, $CDCl_3$):

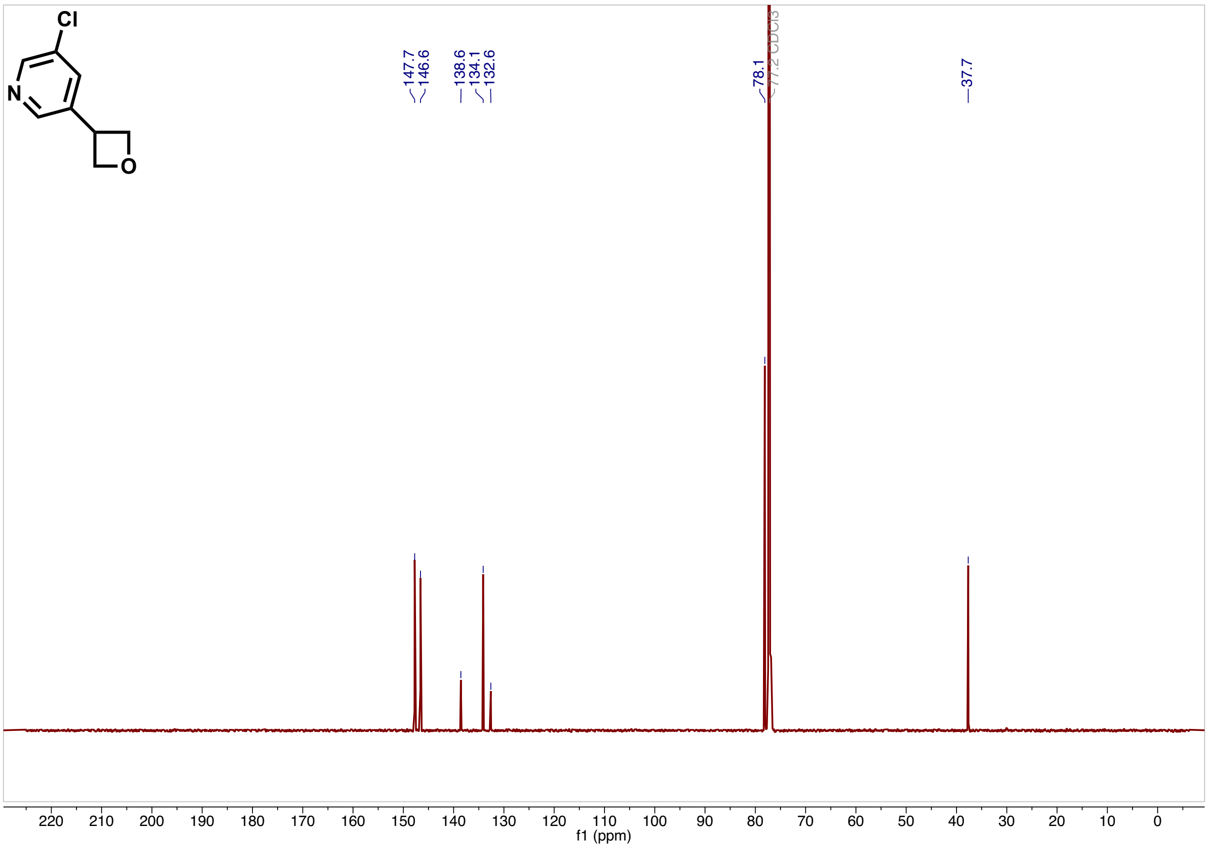

^{1}H NMR of Compound 20 (600 MHz, $CDCl_3$):

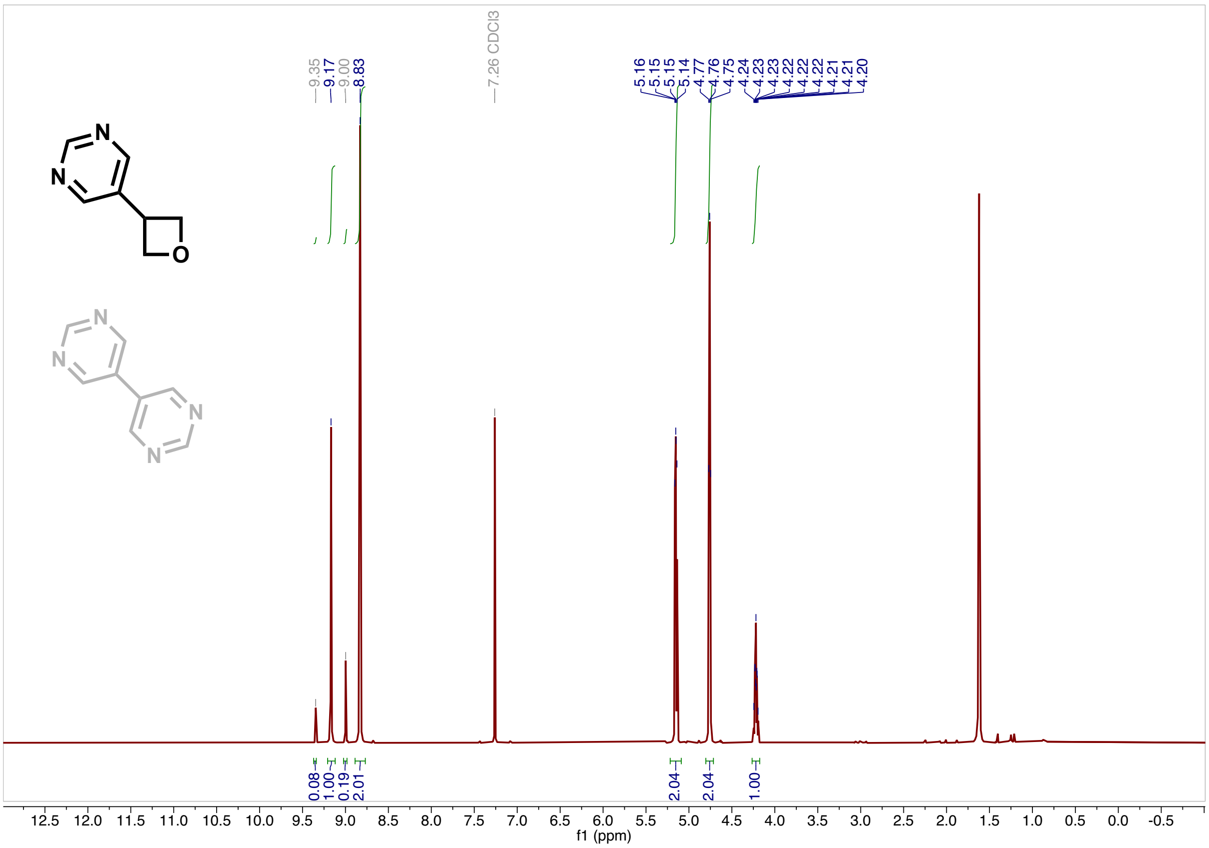

^{13}C NMR of Compound 20 (151 MHz, $CDCl_3$):

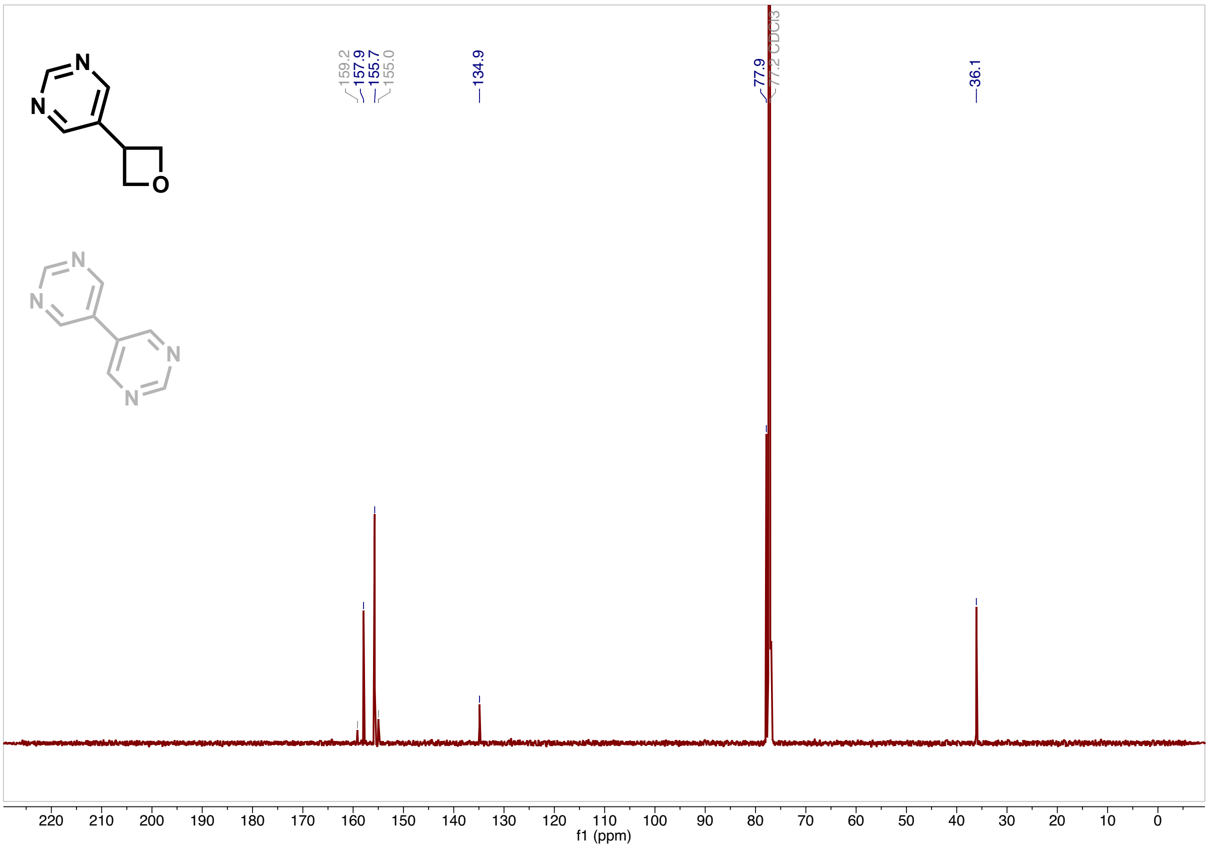

^{1}H NMR of Compound 22 (600 MHz, $CDCl_3$):

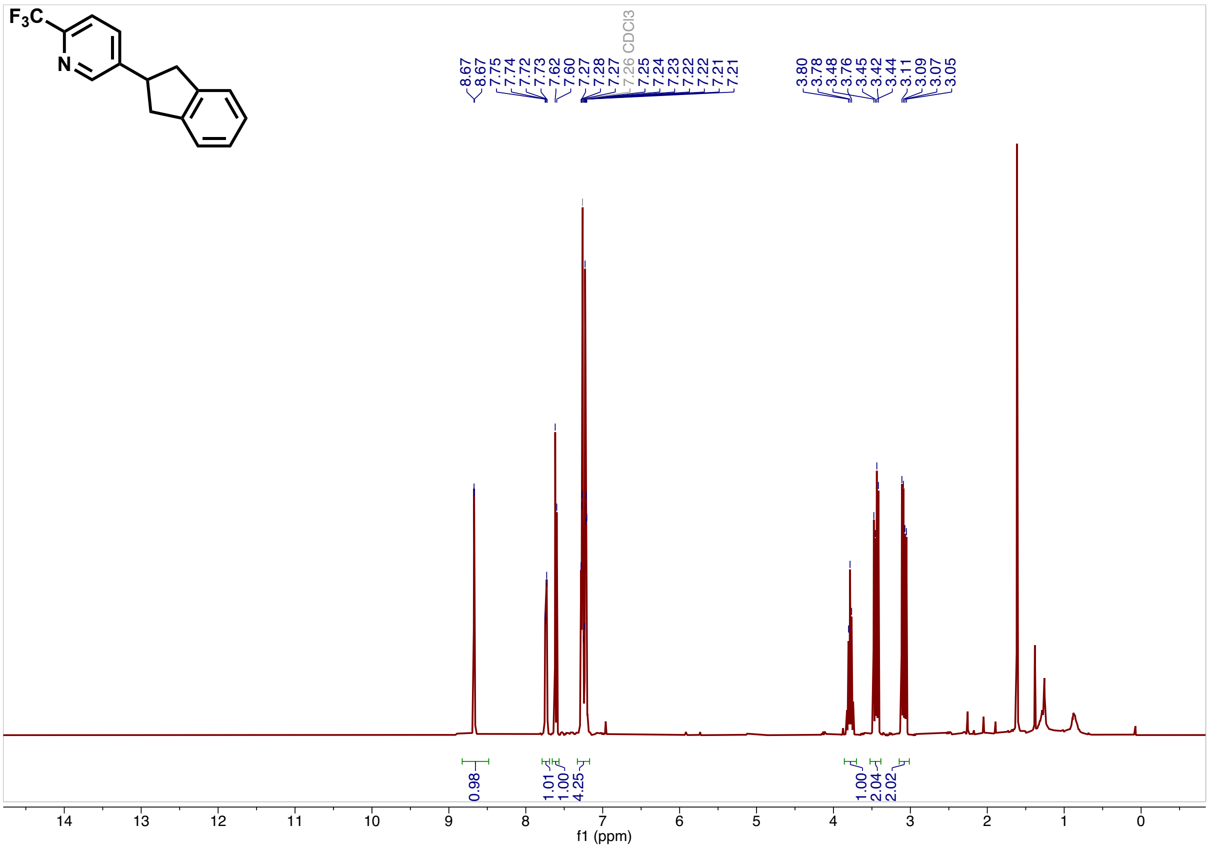

^{13}C NMR of Compound 22 (151 MHz, $CDCl_3$):

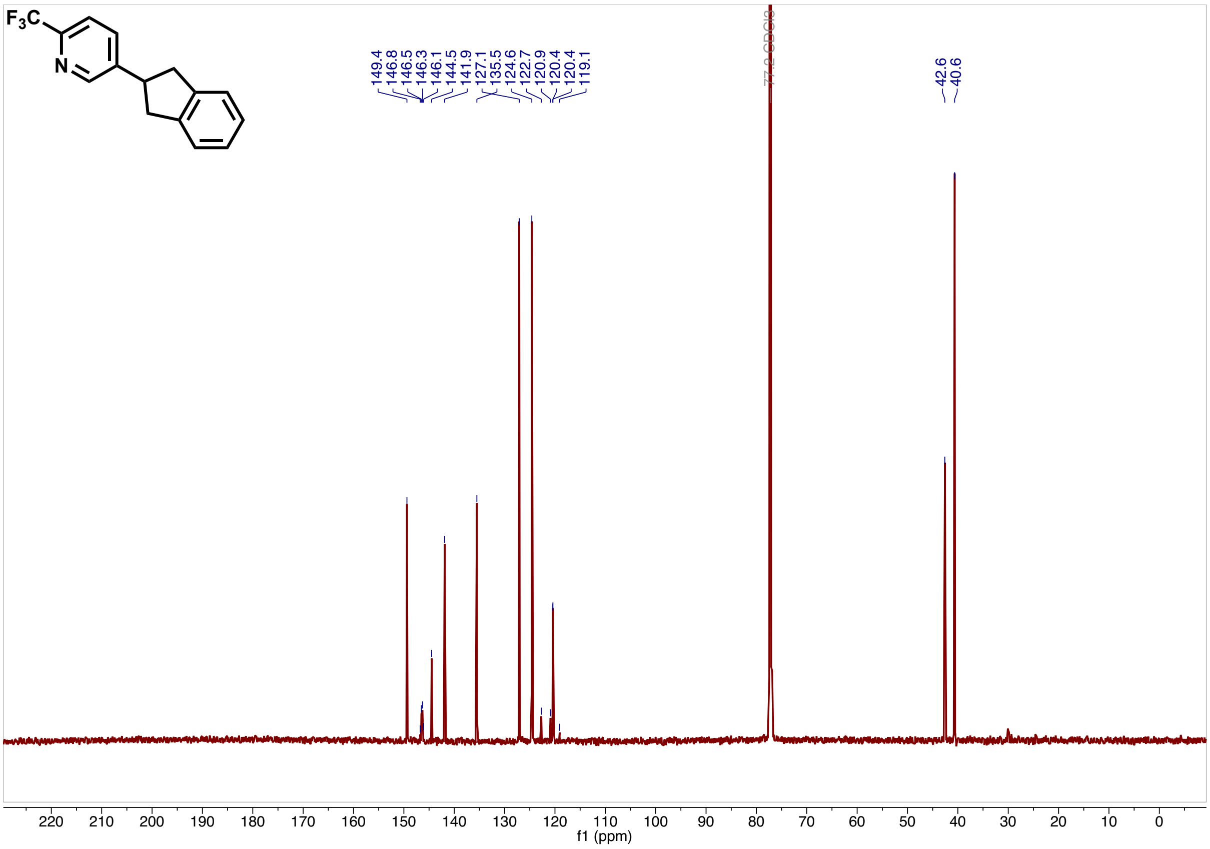

^{19}F NMR of Compound 22 (376 MHz, $CDCl_3$):

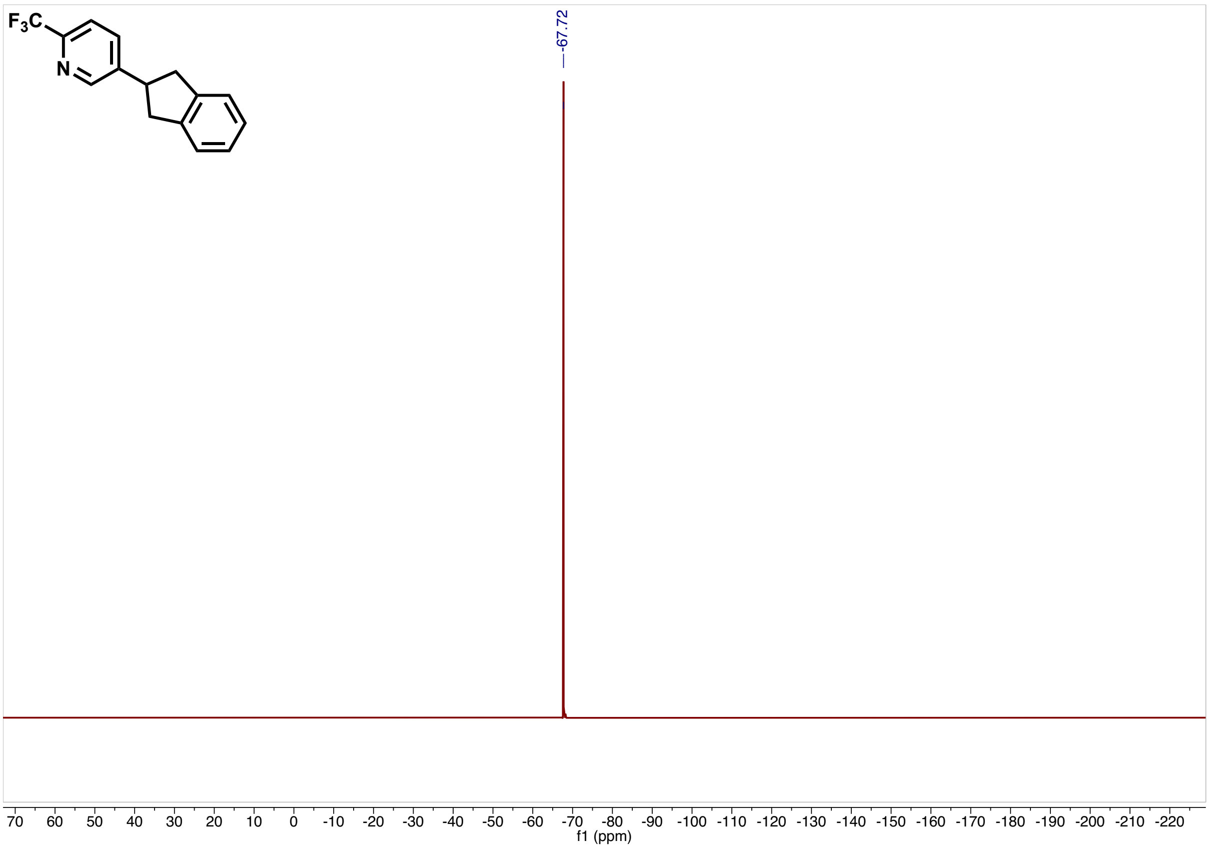

^{1}H NMR of Compound 23 (600 MHz, $CDCl_3$):

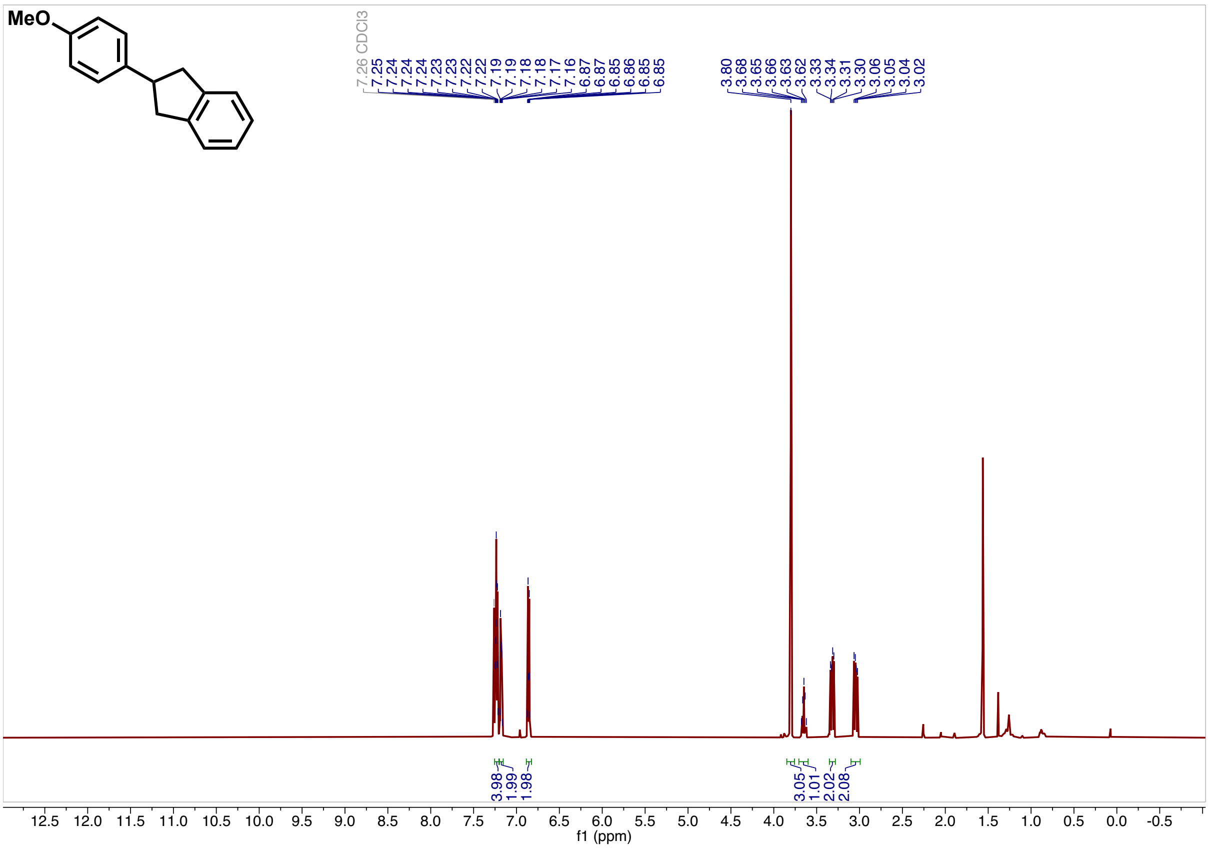

^{13}C NMR of Compound 23 (151 MHz, $CDCl_3$):

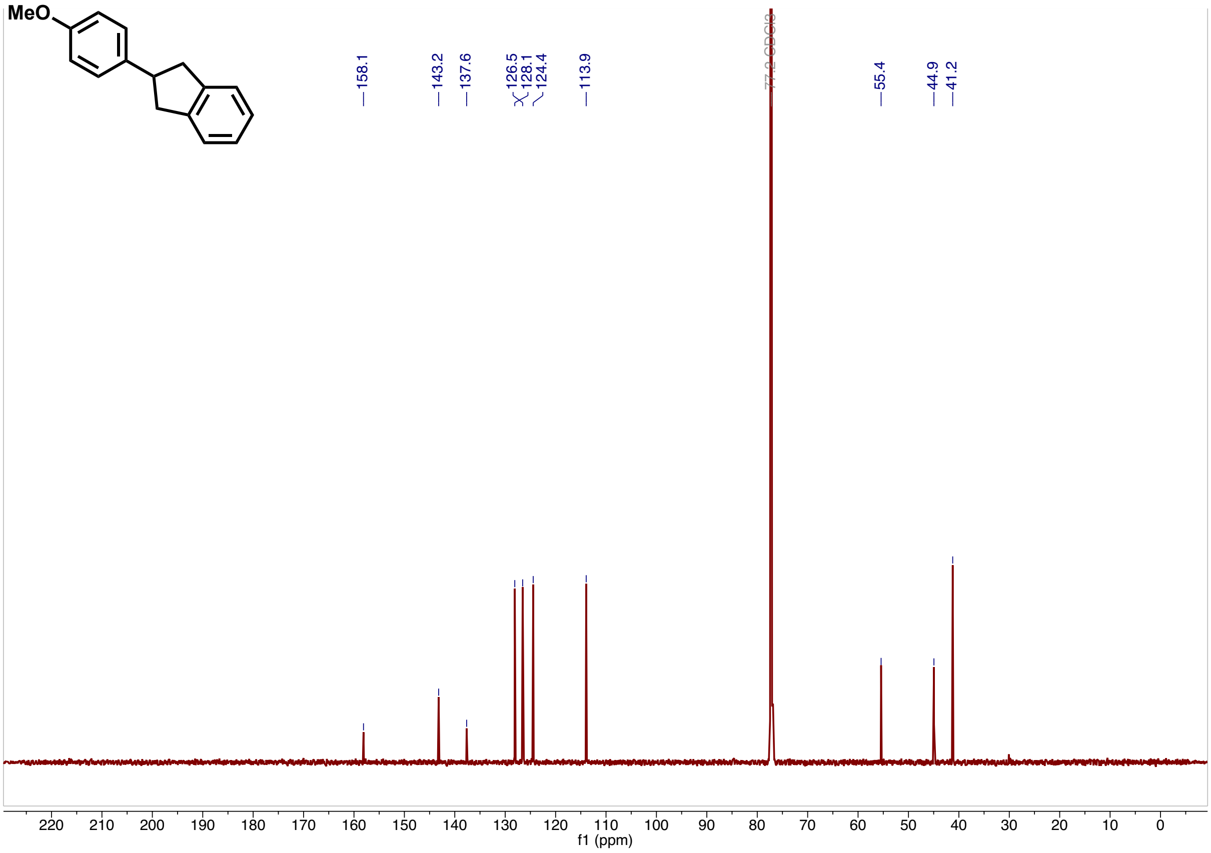

^{1}H NMR of Compound 24 (600 MHz, $CDCl_3$):

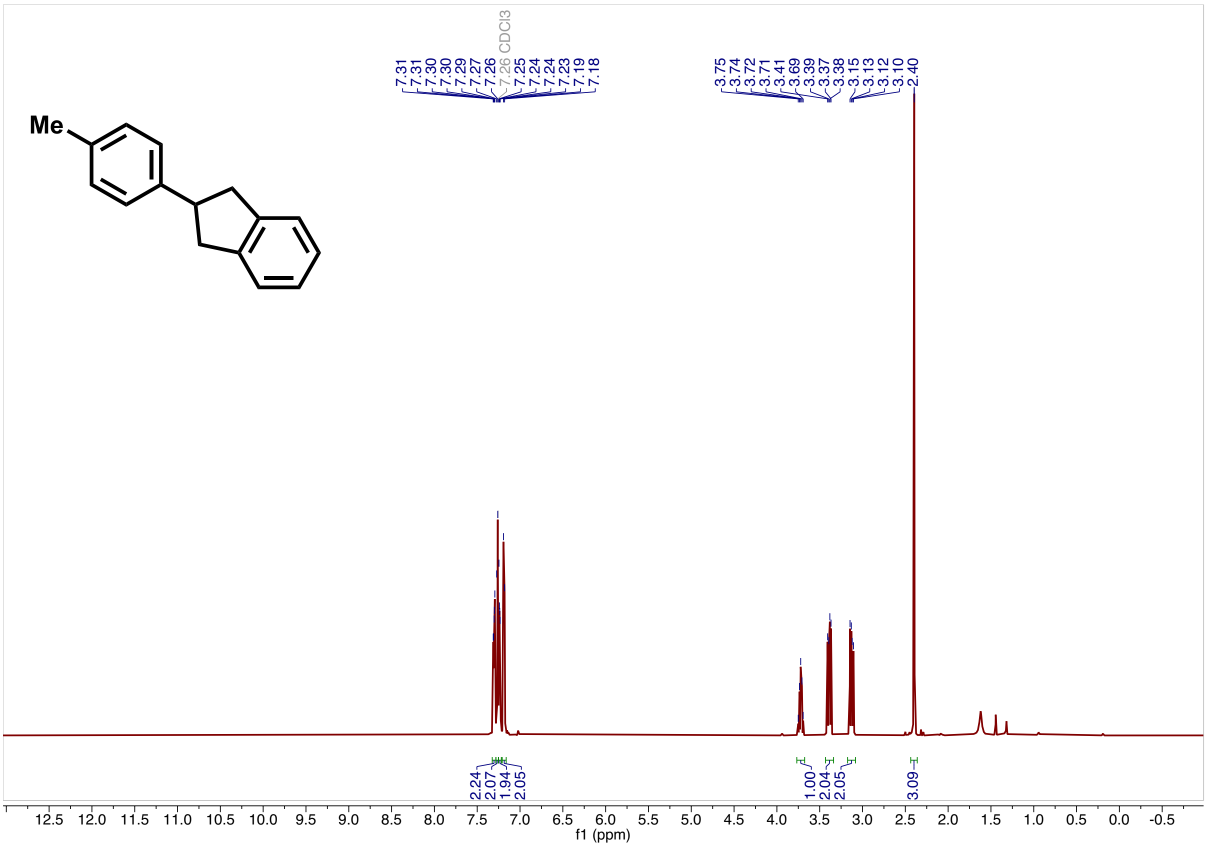

^{13}C NMR of Compound 24 (151 MHz, $CDCl_3$):

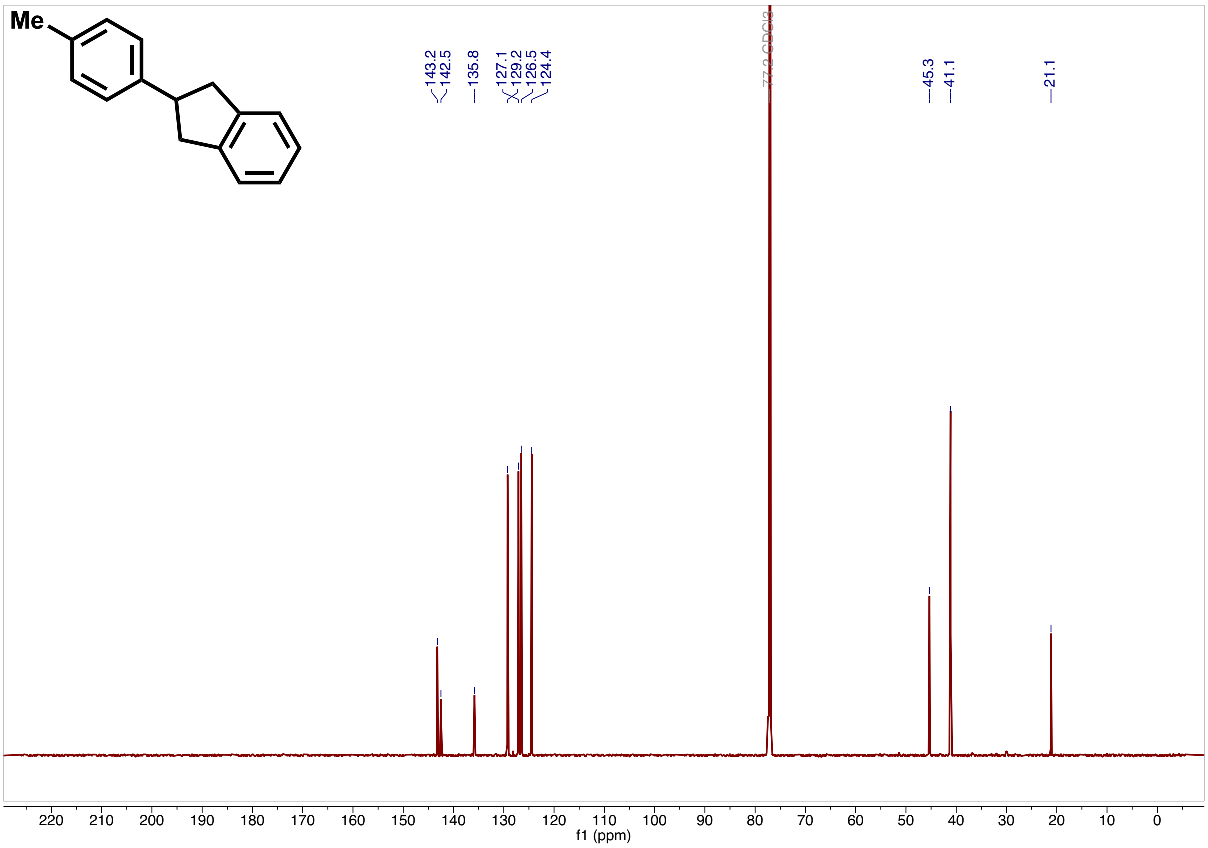

^{1}H NMR of Compound 25 (600 MHz, $CDCl_3$):

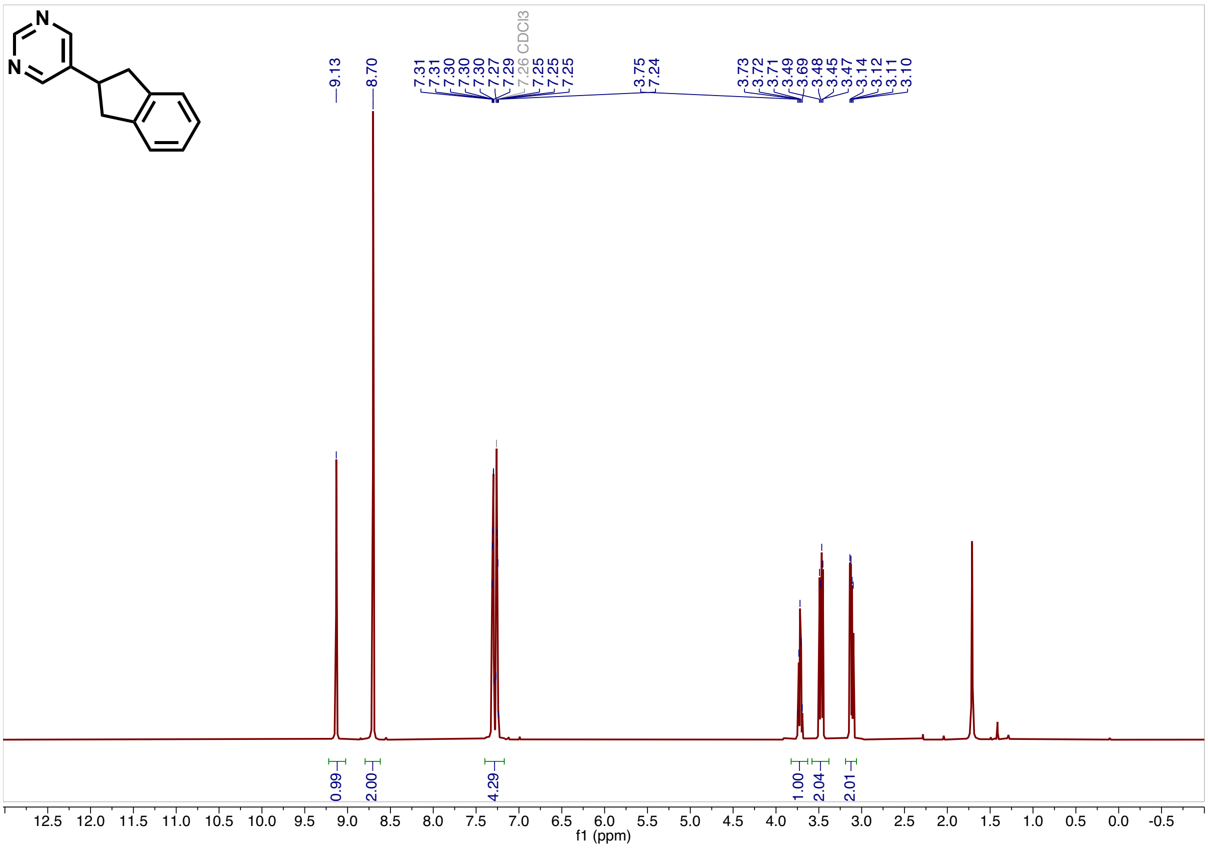

^{13}C NMR of Compound 25 (151 MHz, $CDCl_3$):

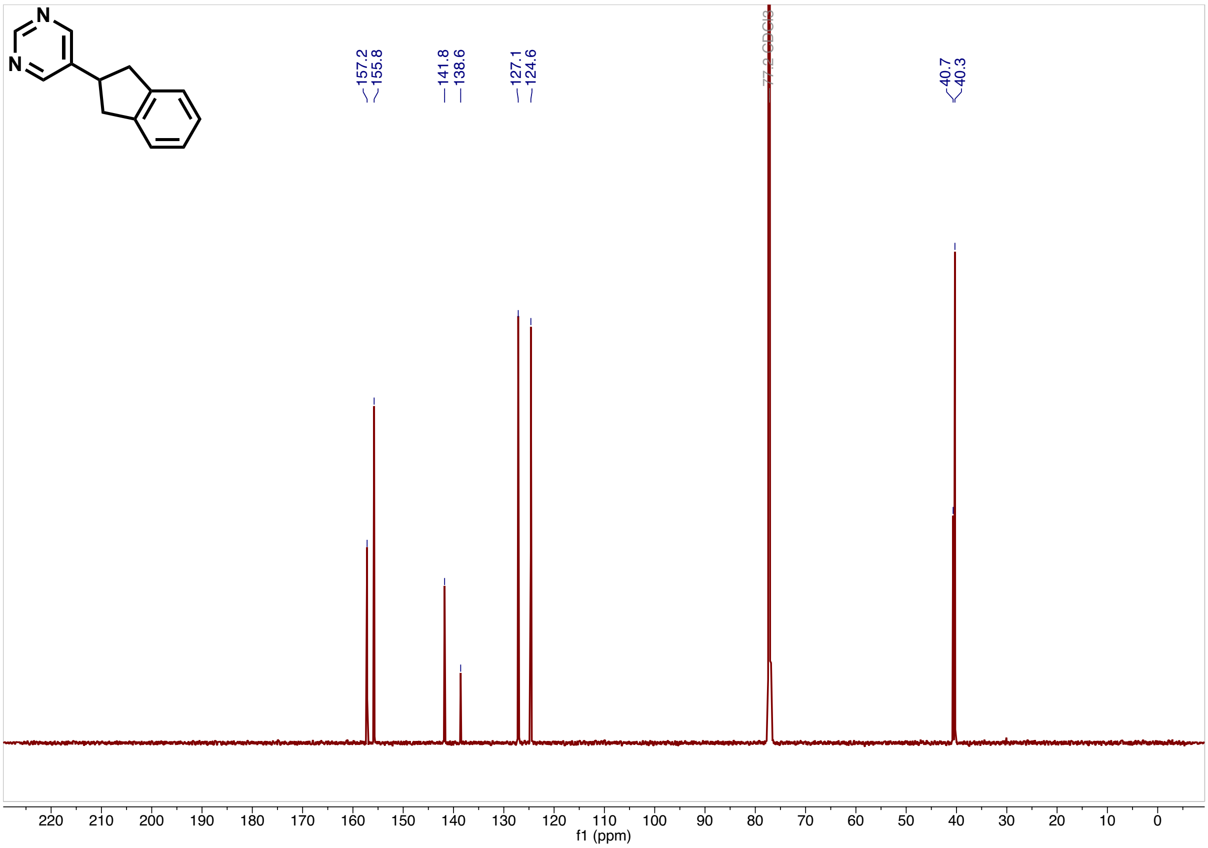

^{1}H NMR of Compound 26 (600 MHz, $CDCl_3$):

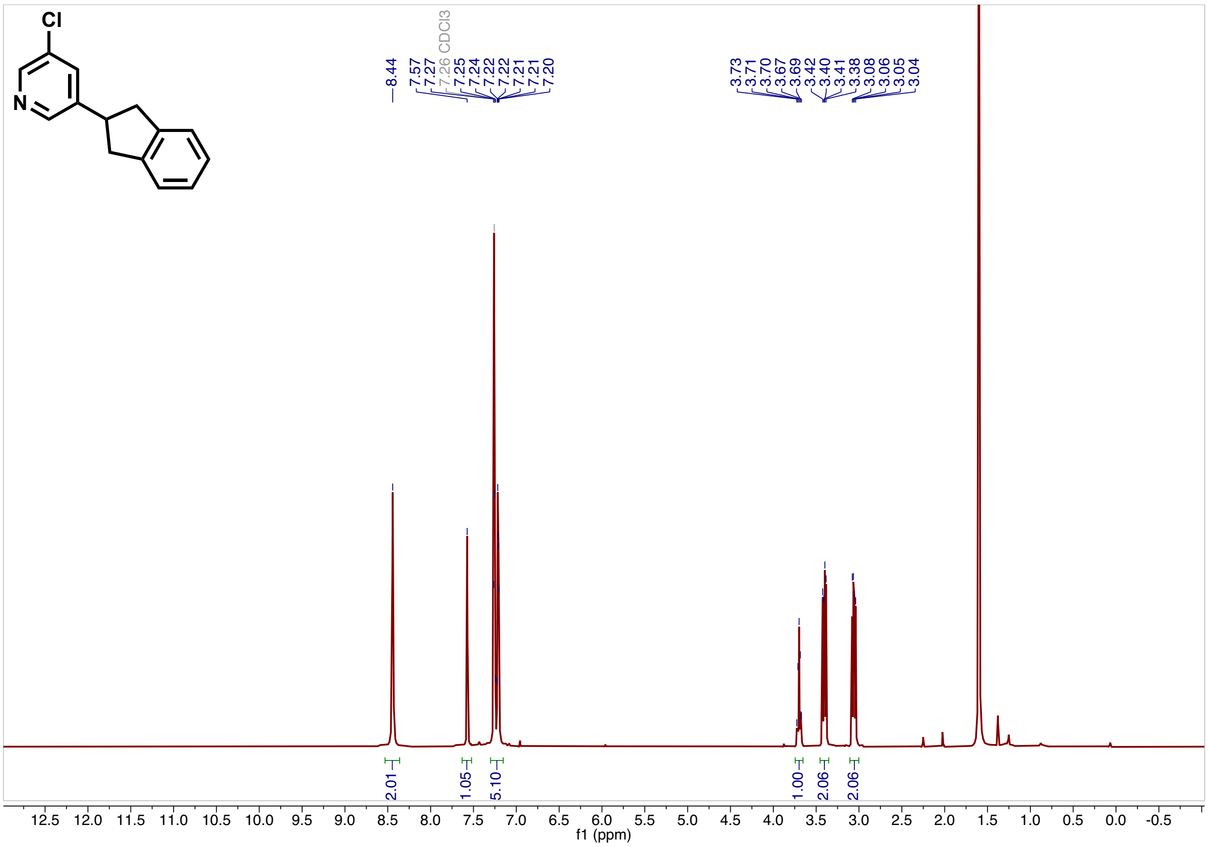

^{13}C NMR of Compound 26 (151 MHz, $CDCl_3$):

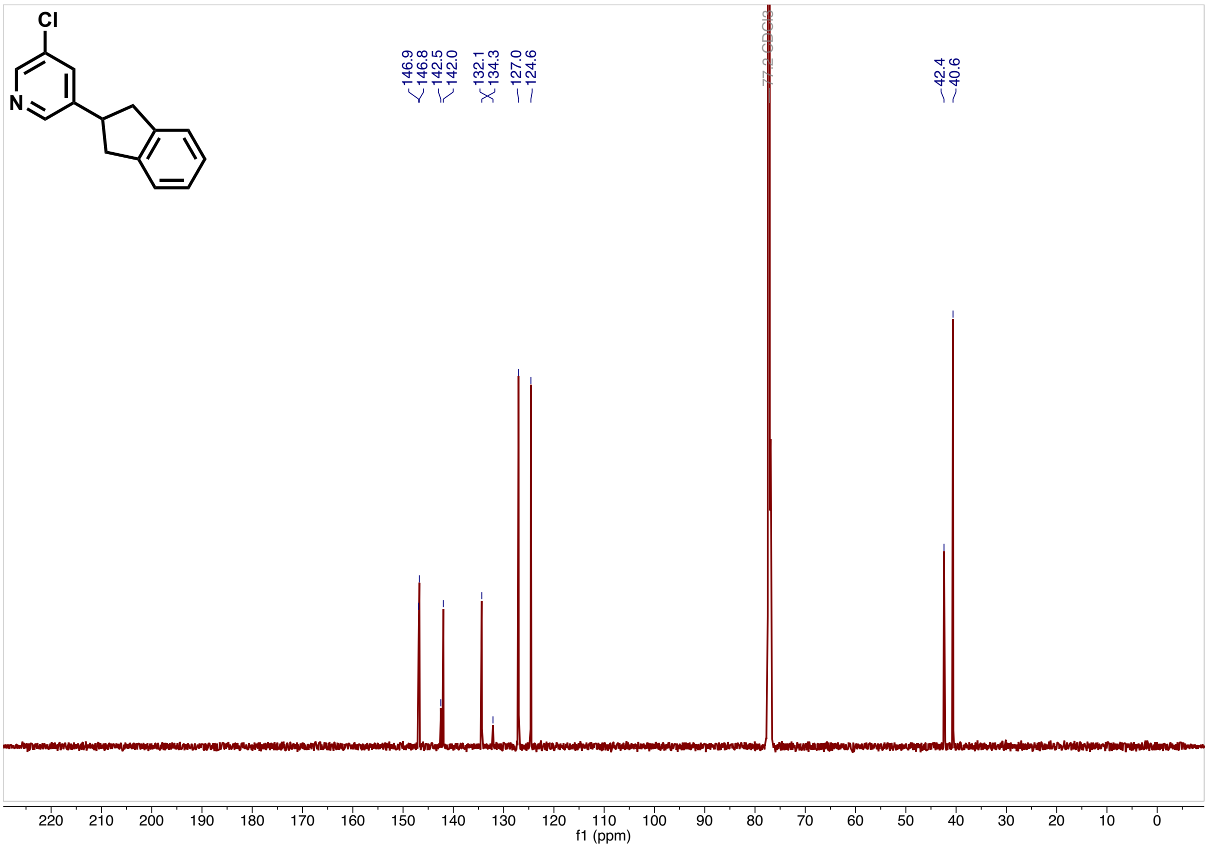

^{1}H NMR of Compound 28 (600 MHz, $CDCl_3$):

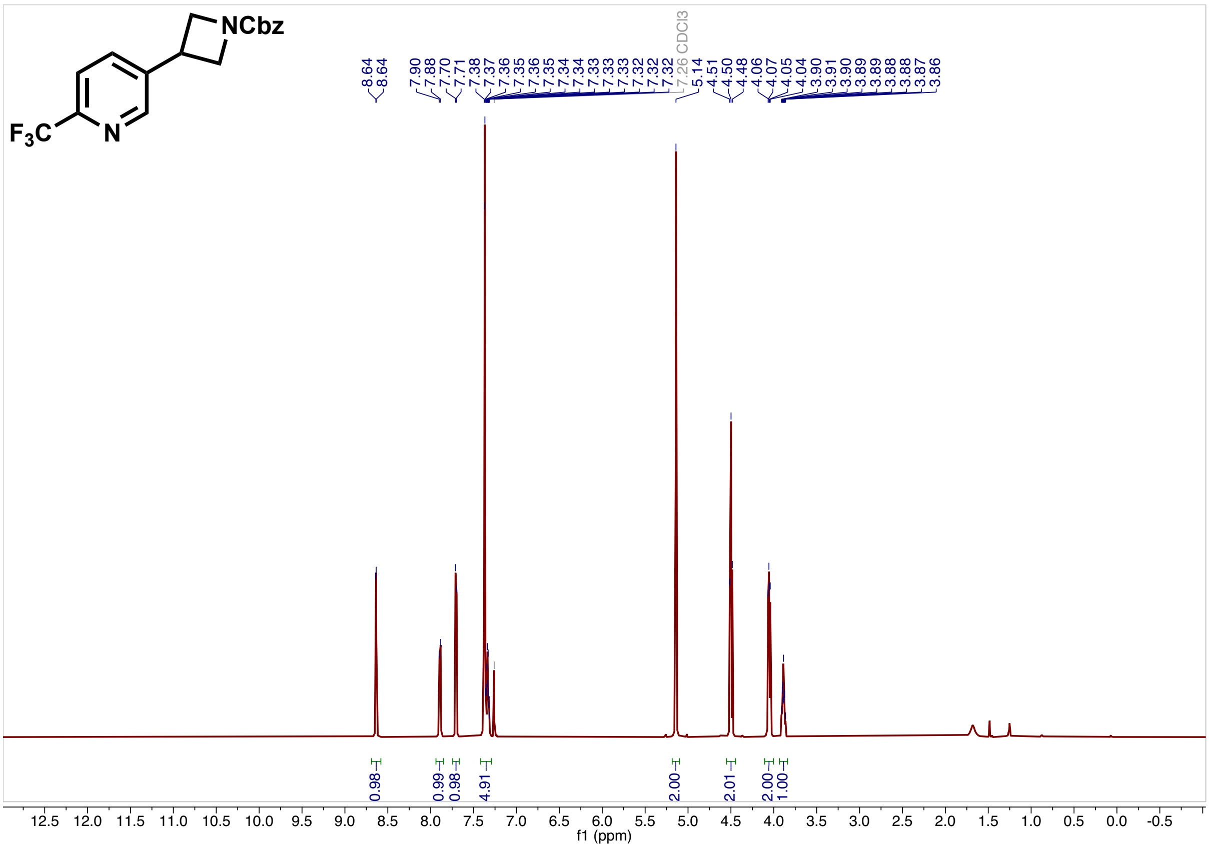

^{13}C NMR of Compound 28 (151 MHz, $CDCl_3$):

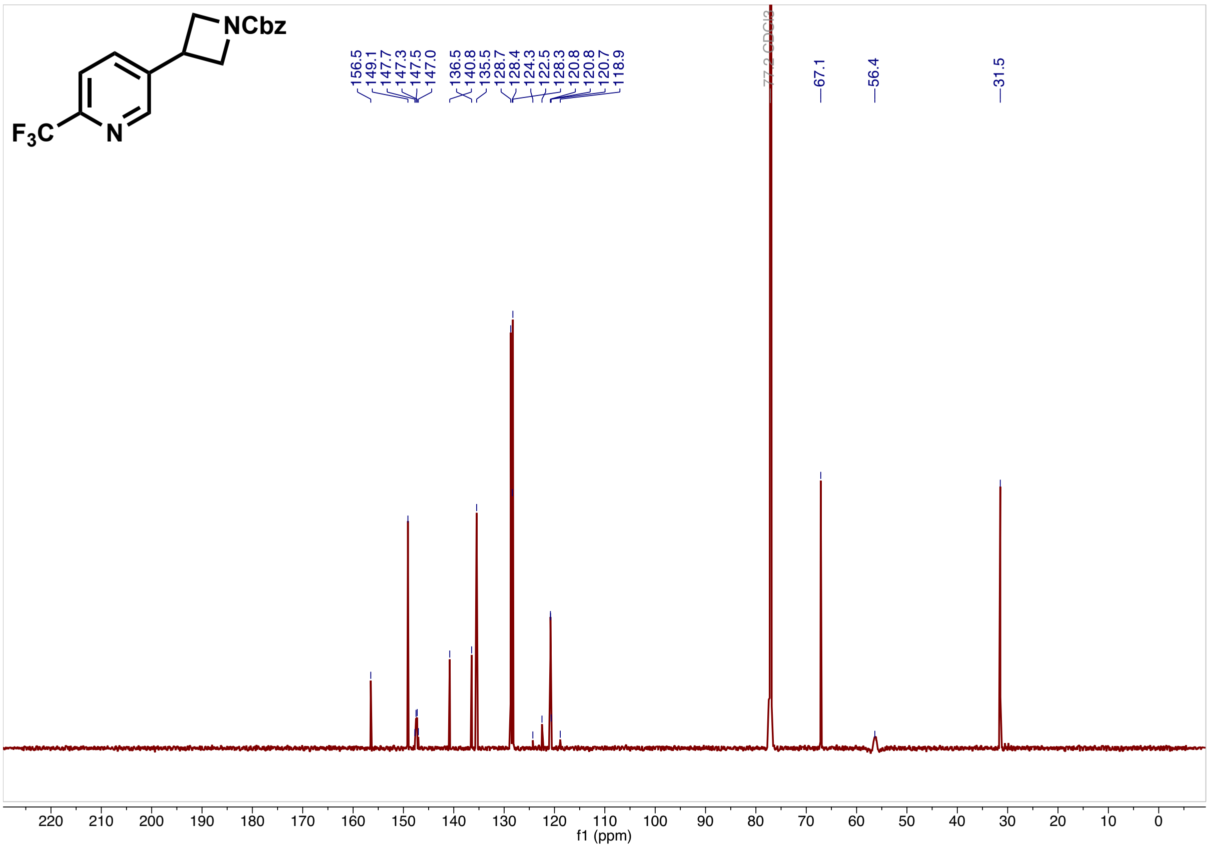

^{19}F NMR of Compound 28 (376 MHz, $CDCl_3$):

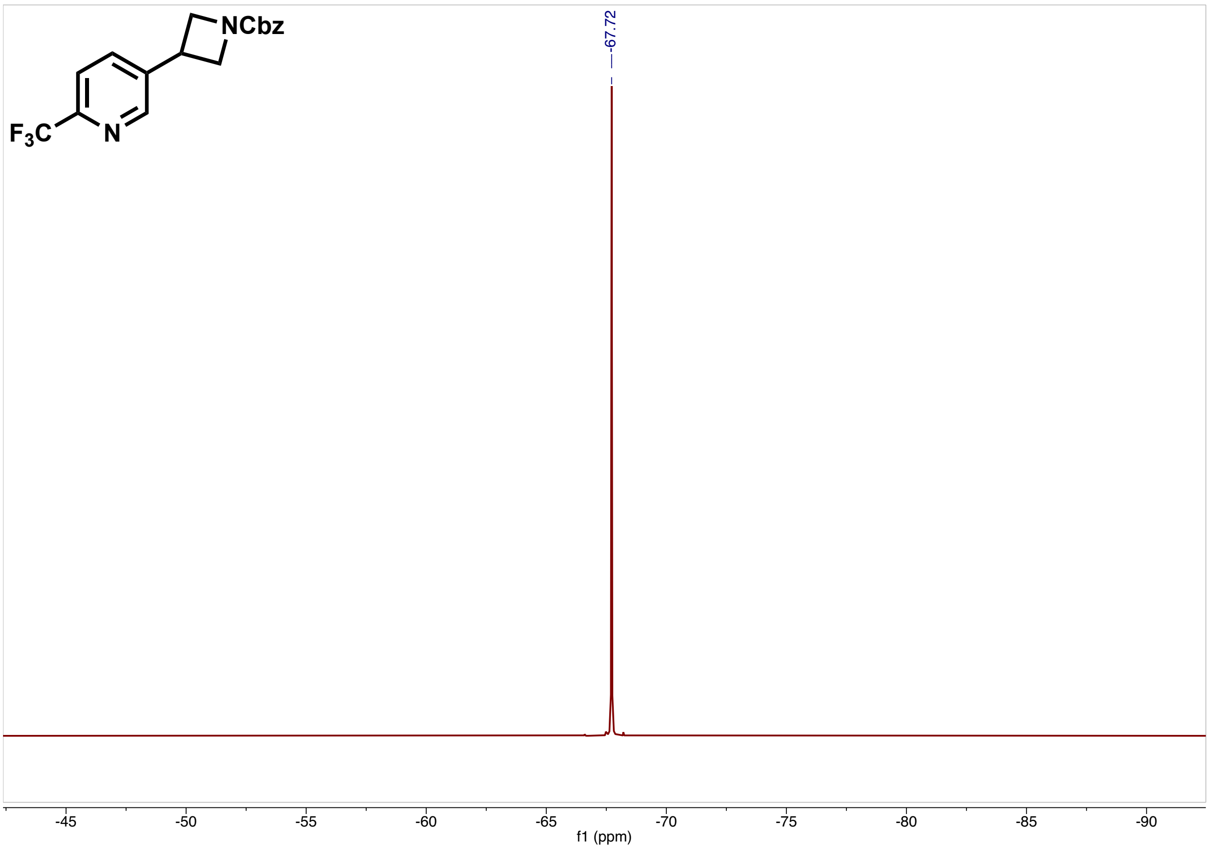

^{1}H NMR of Compound 29 (600 MHz, $CDCl_3$):

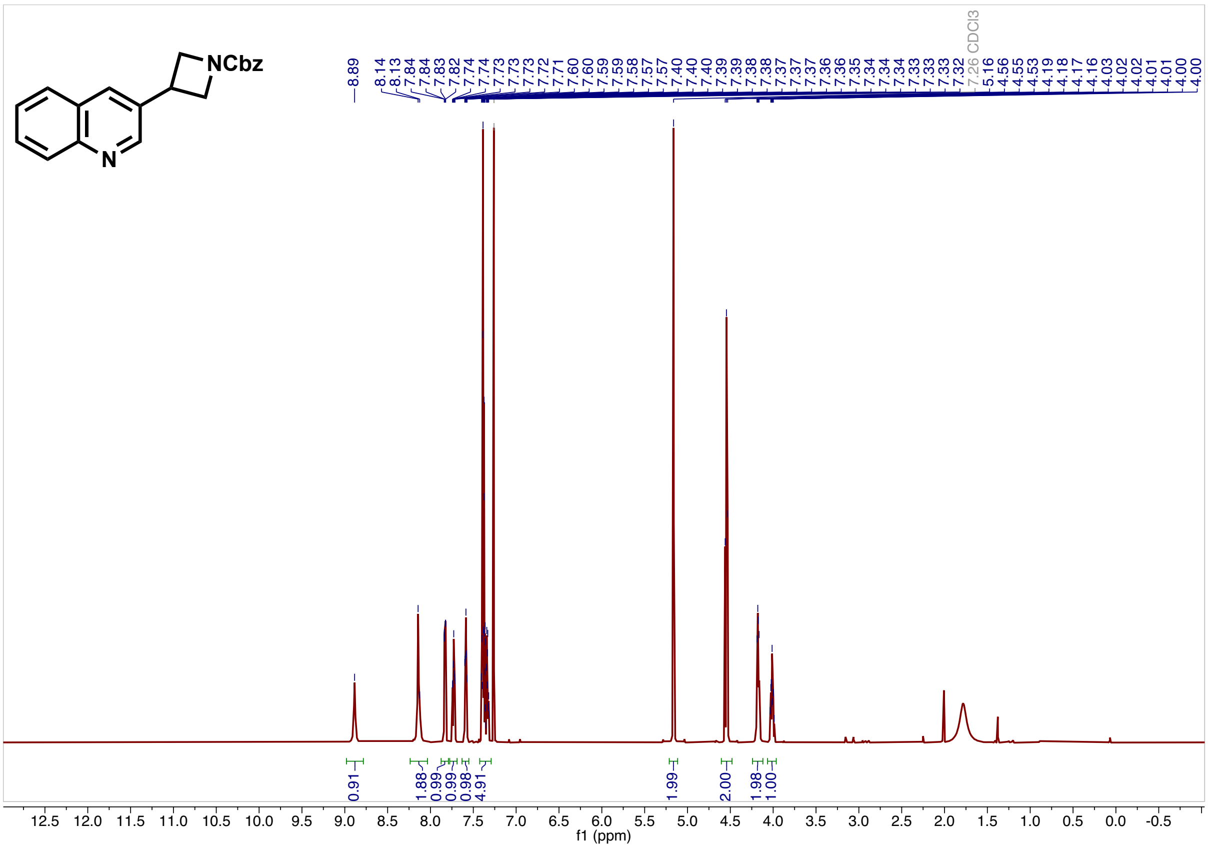

^{13}C NMR of Compound 29 (151 MHz, $CDCl_3$):

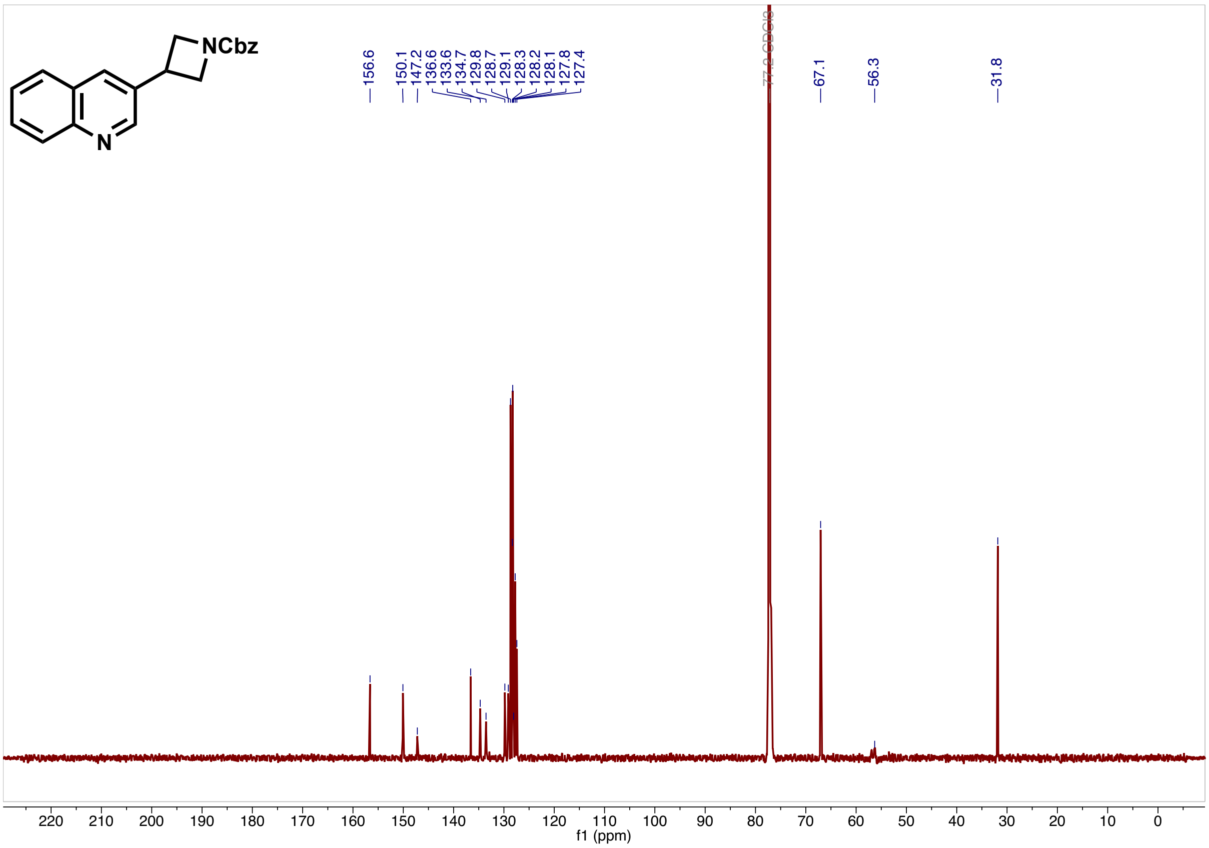

^{1}H NMR of Compound 30 (600 MHz, $CDCl_3$):

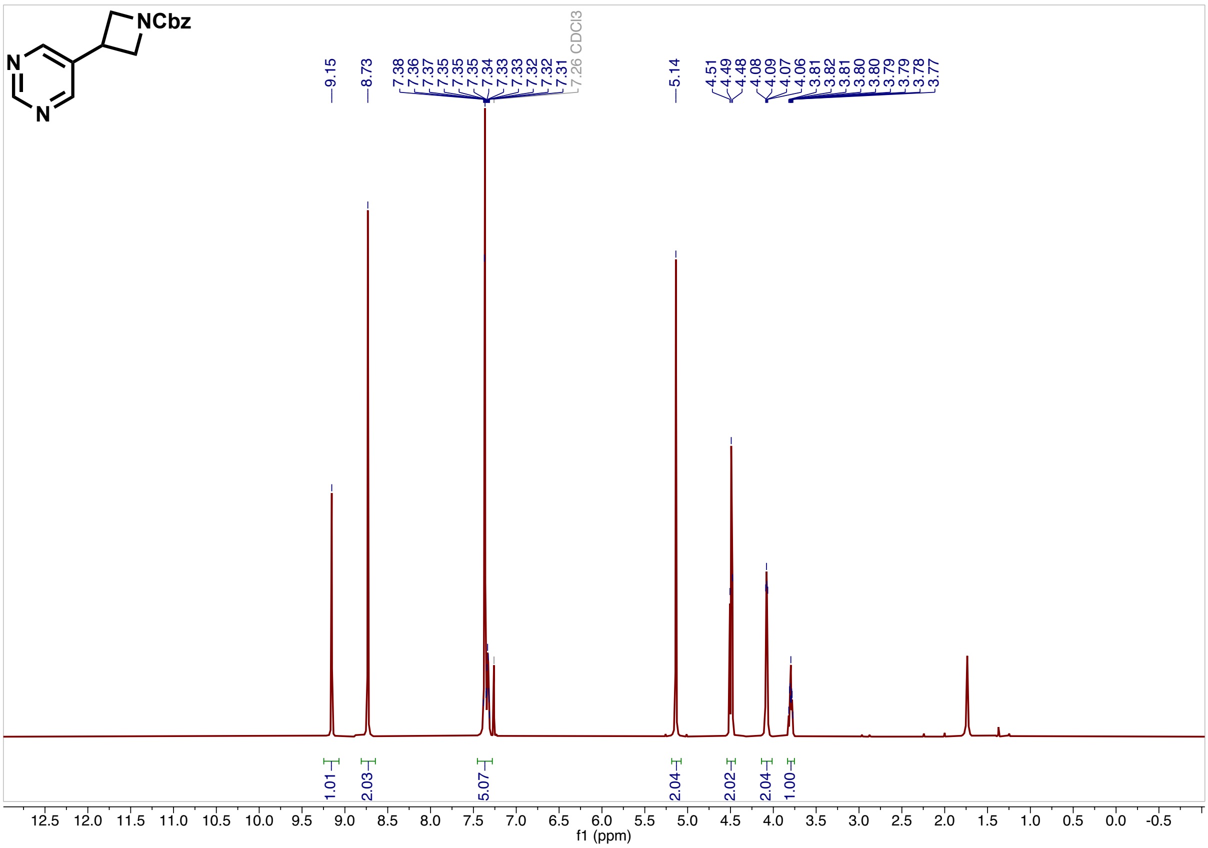

^{13}C NMR of Compound 30 (151 MHz, $CDCl_3$

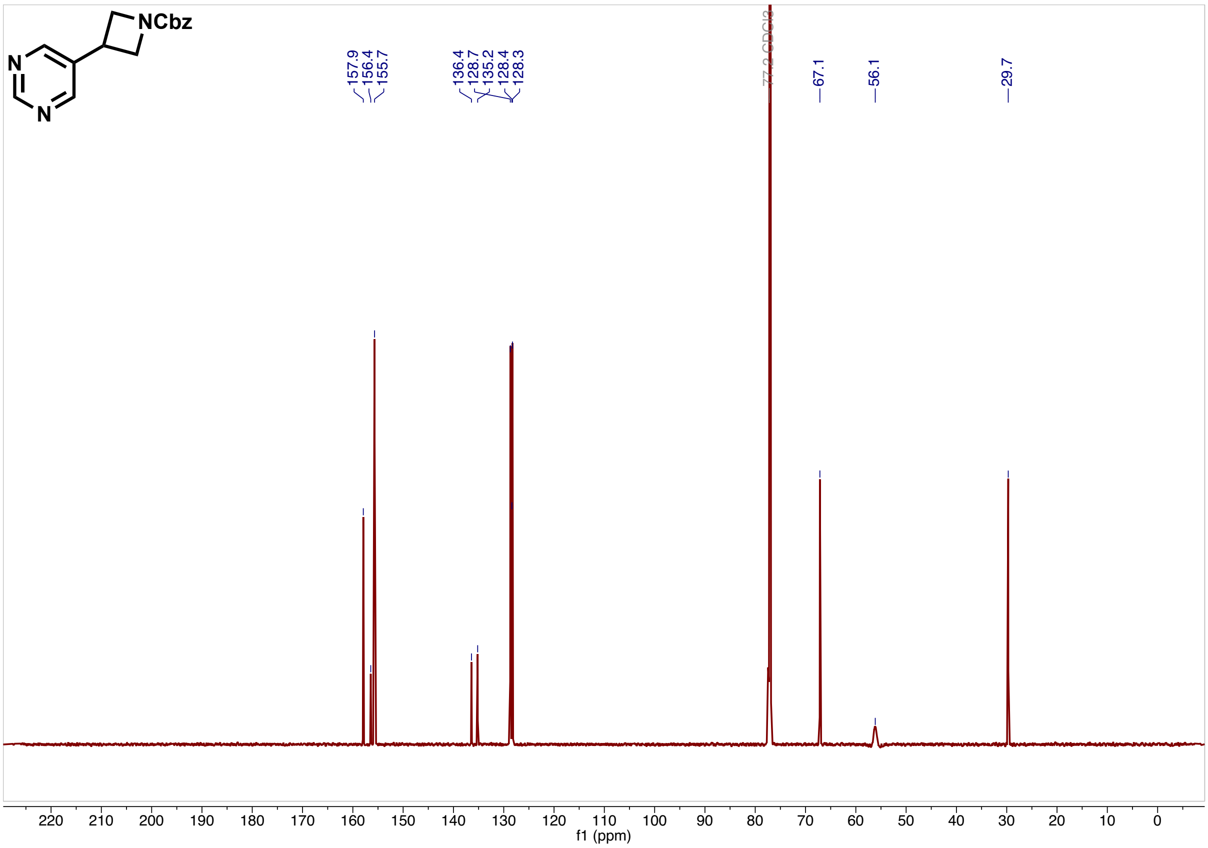

^{1}H NMR of Compound 31 (600 MHz, $CDCl_3$):

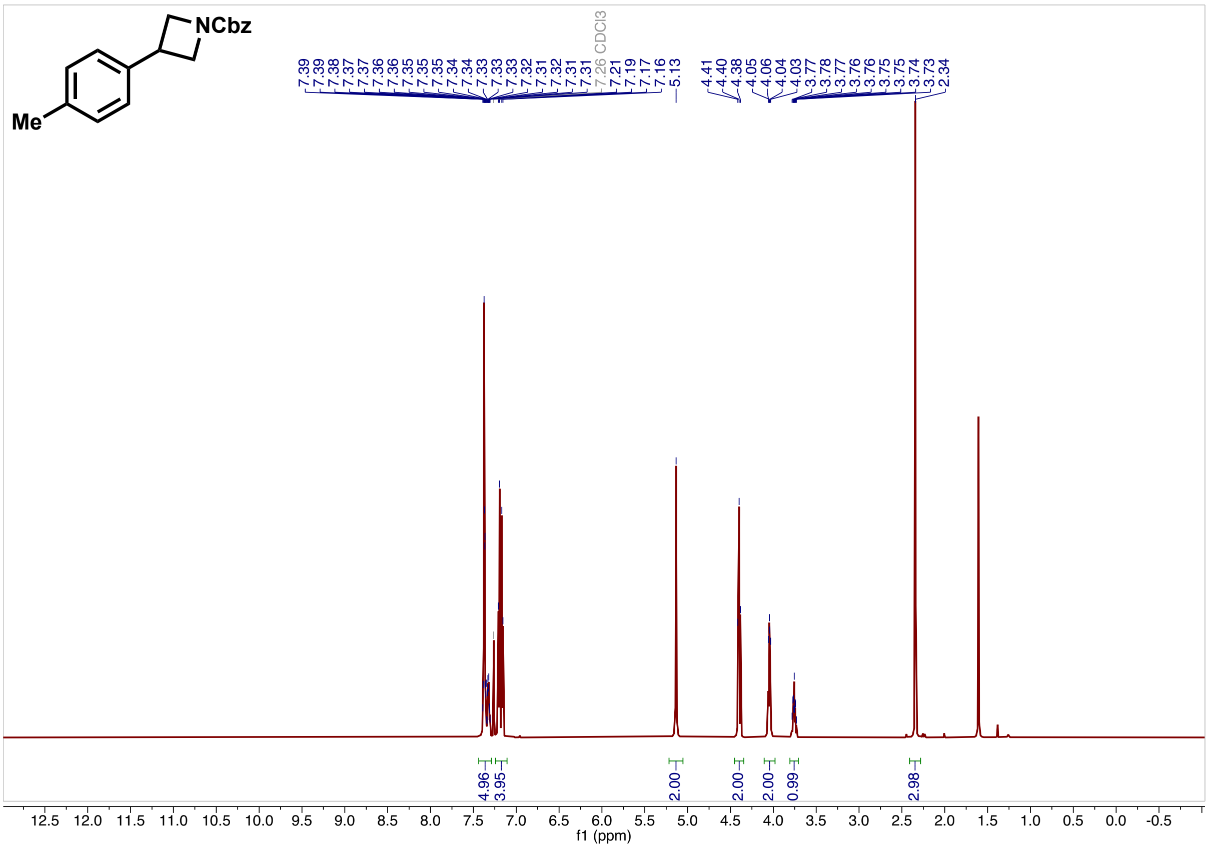

^{13}C NMR of Compound 31 (151 MHz, $CDCl_3$):

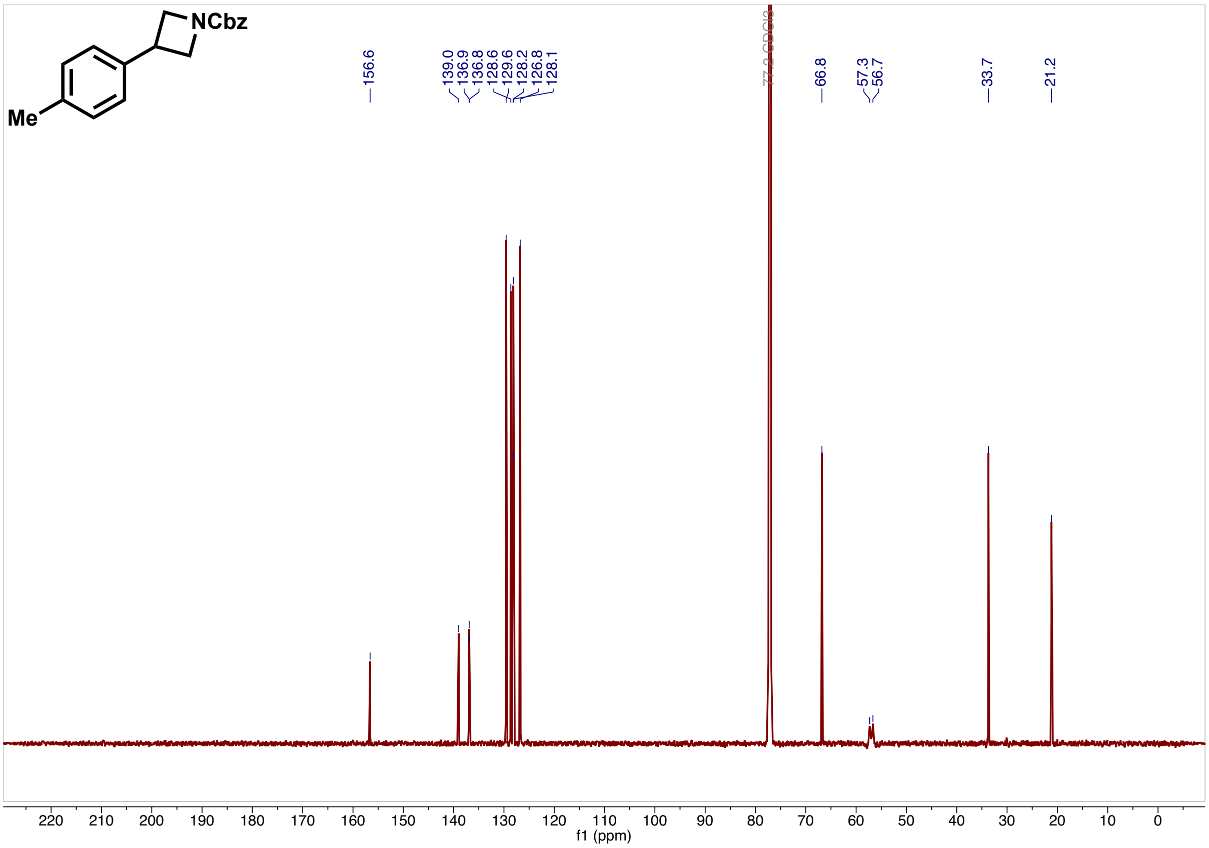

^{1}H NMR of Compound 32 (600 MHz, $CDCl_3$):

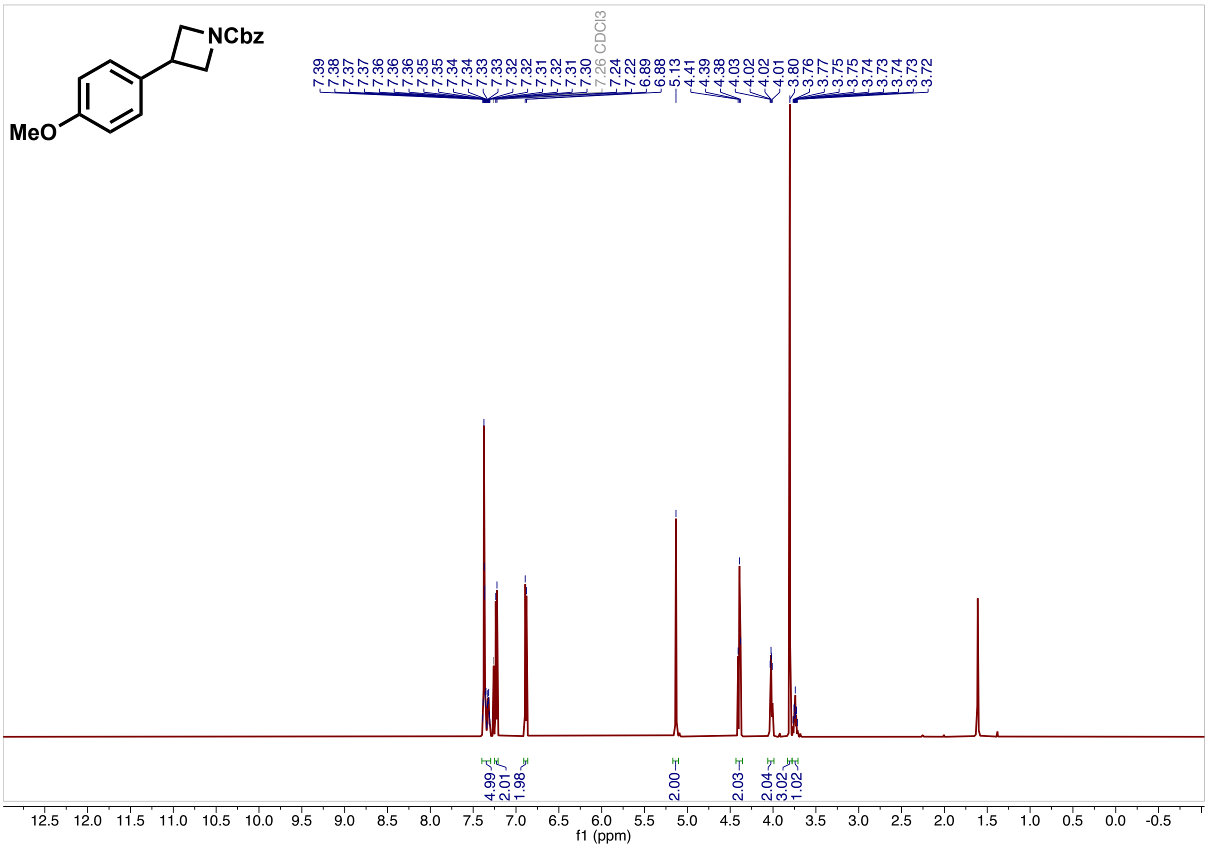

^{13}C NMR of Compound 32 (151 MHz, $CDCl_3$):

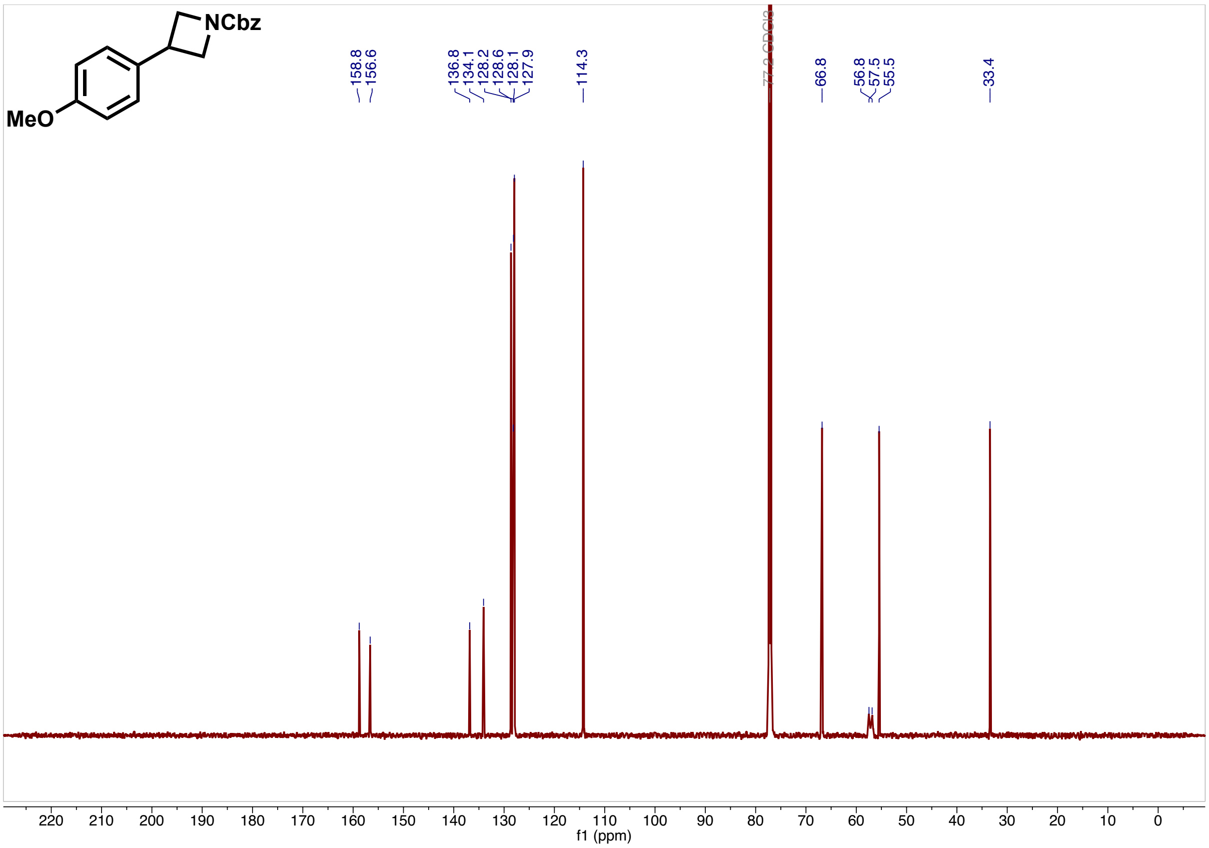

^{1}H NMR of Compound 33 (600 MHz, $CDCl_3$):

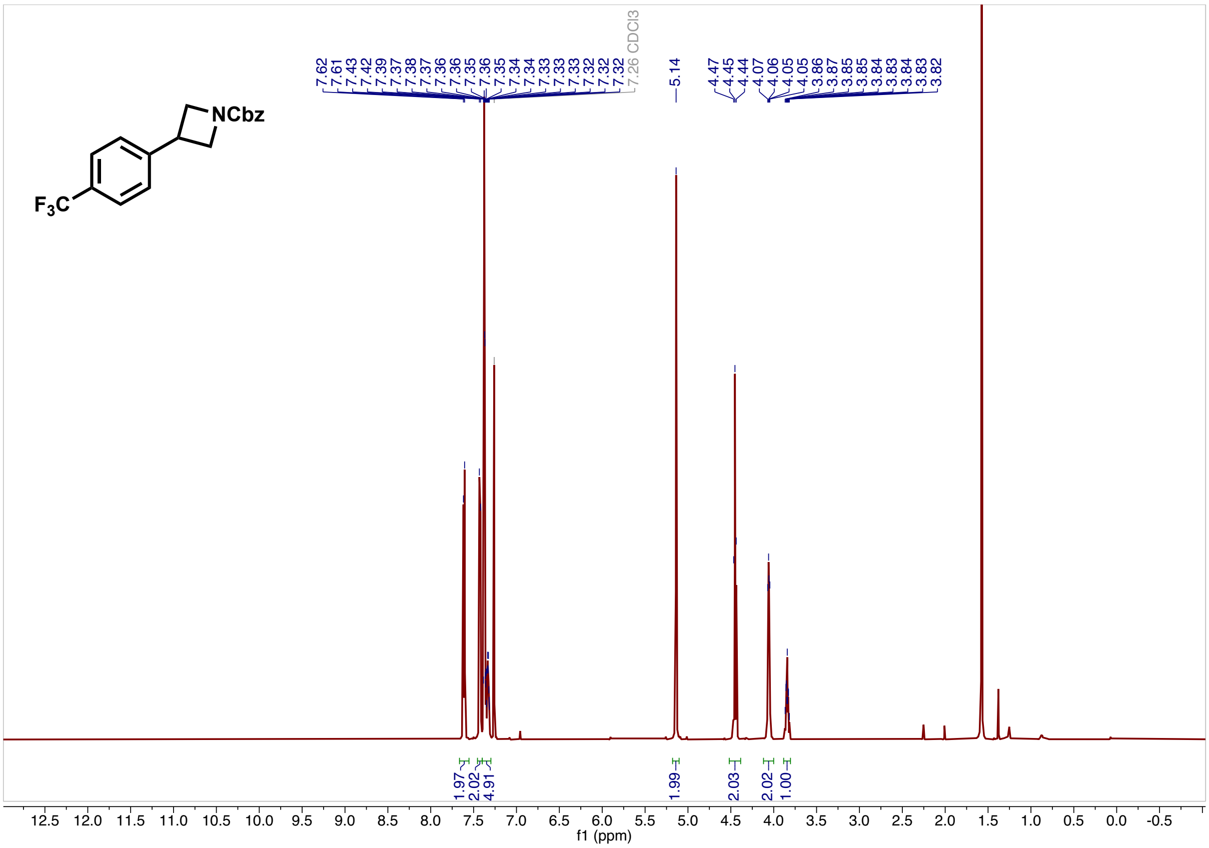

^{13}C NMR of Compound 33 (151 MHz, $CDCl_3$):

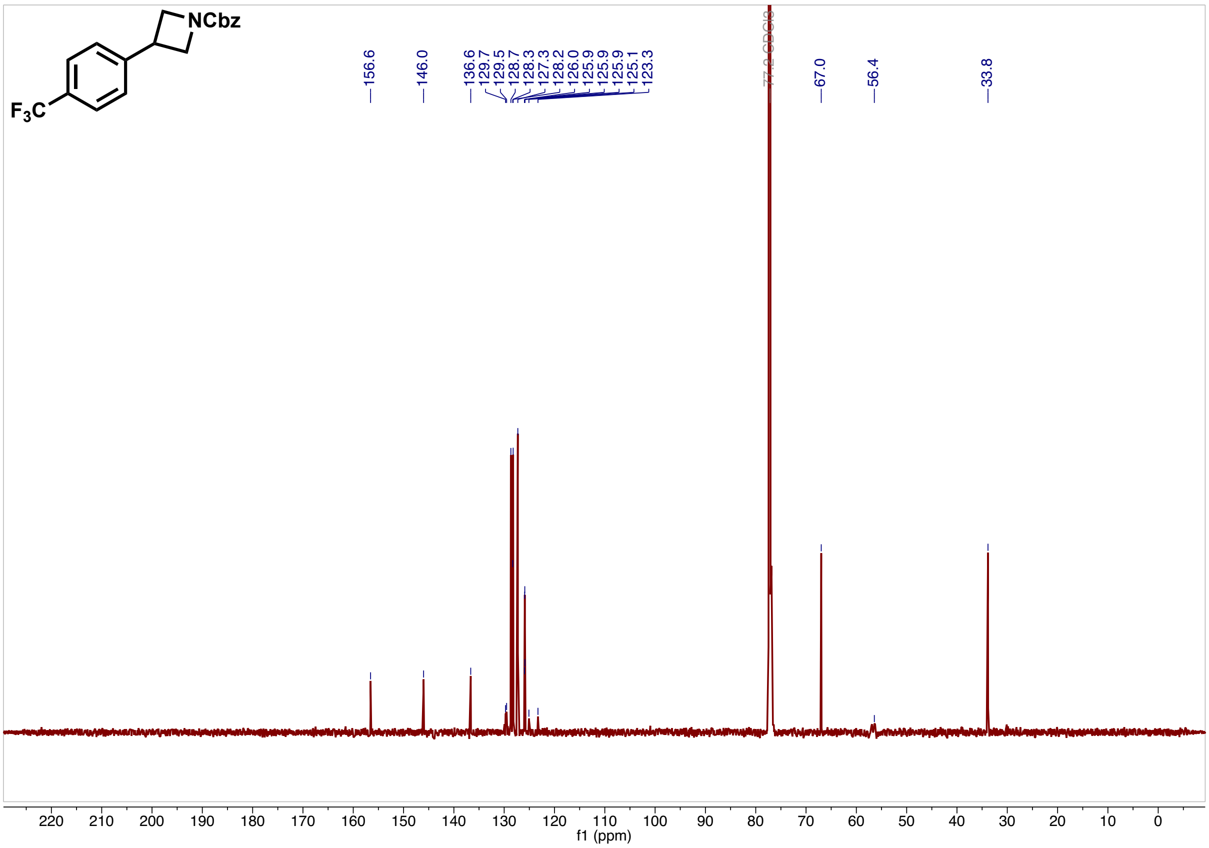

^{19}F NMR of Compound 33 (376 MHz, $CDCl_3$):

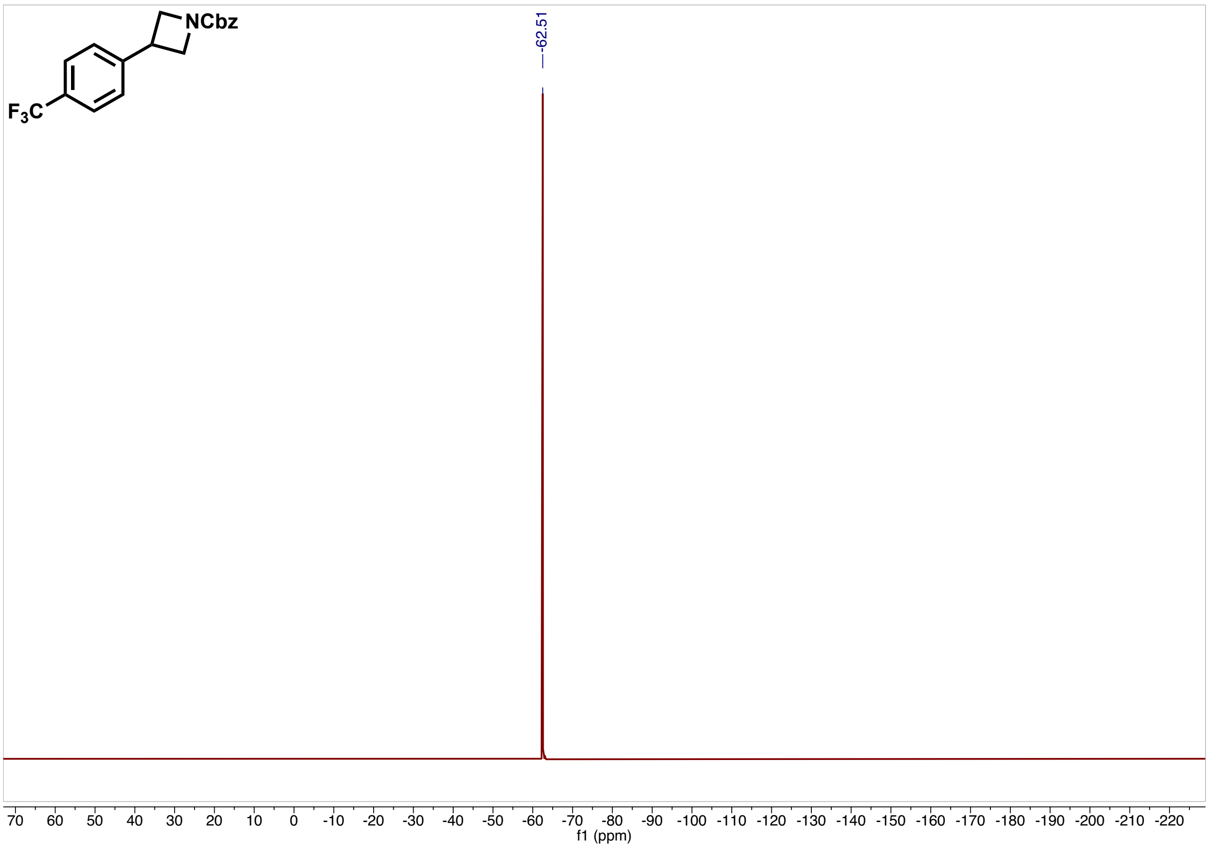

^{1}H NMR of Compound 34 (600 MHz, $CDCl_3$):

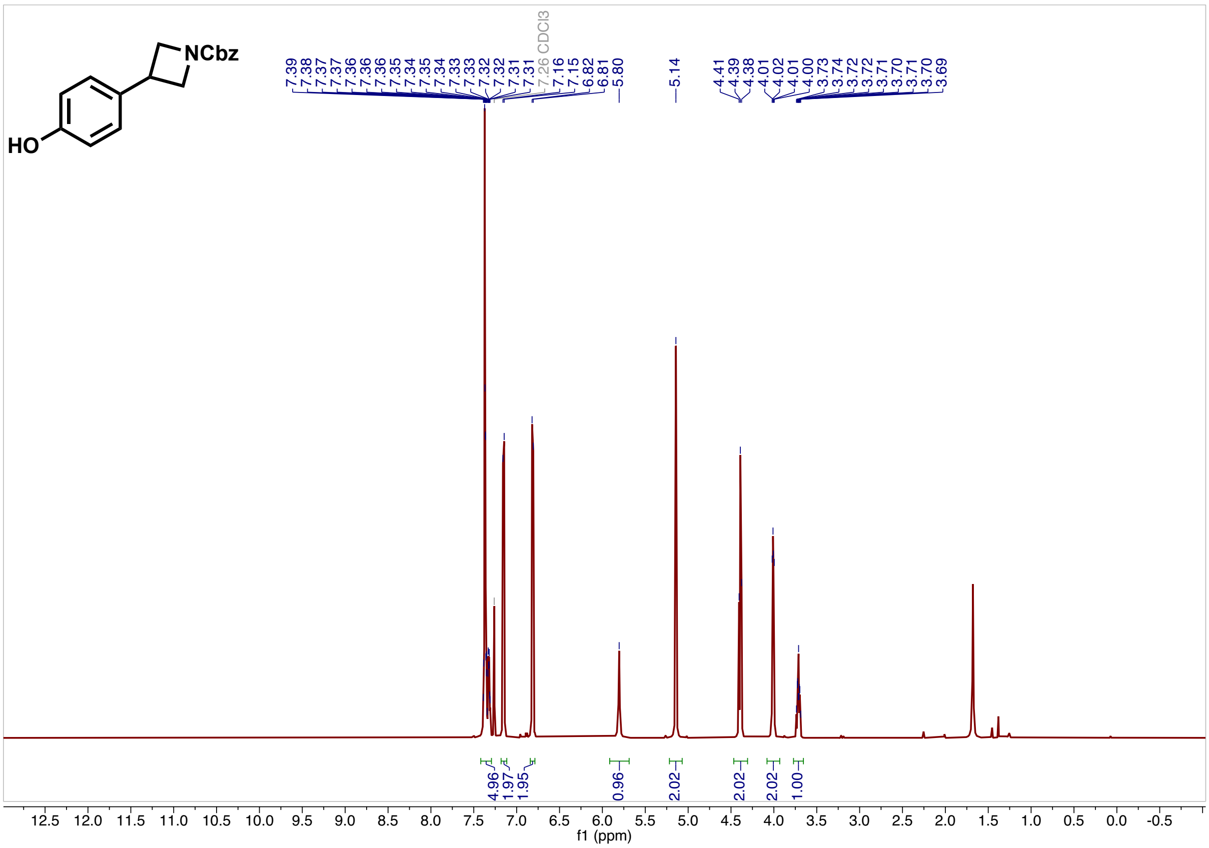

^{13}C NMR of Compound 34 (151 MHz, $CDCl_3$):

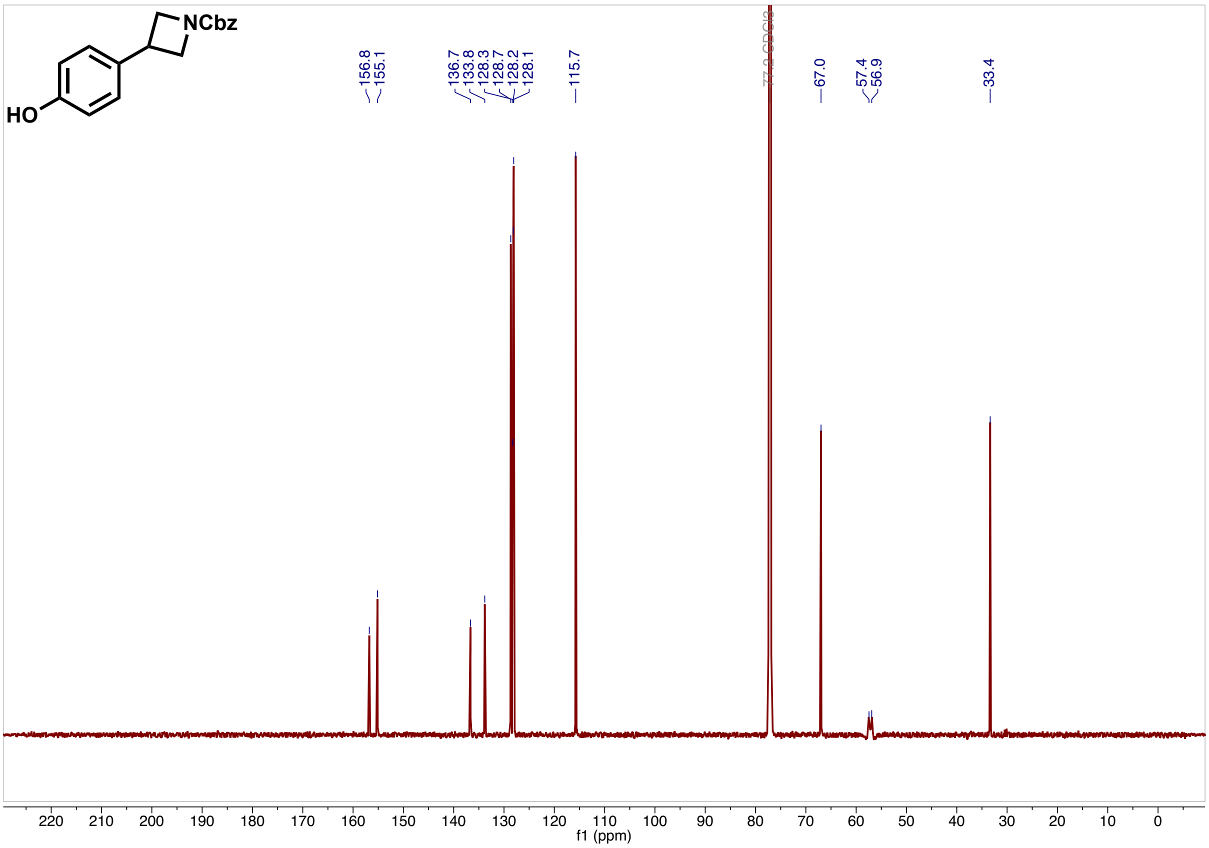

^{1}H NMR of Compound 35 (600 MHz, $CDCl_3$):

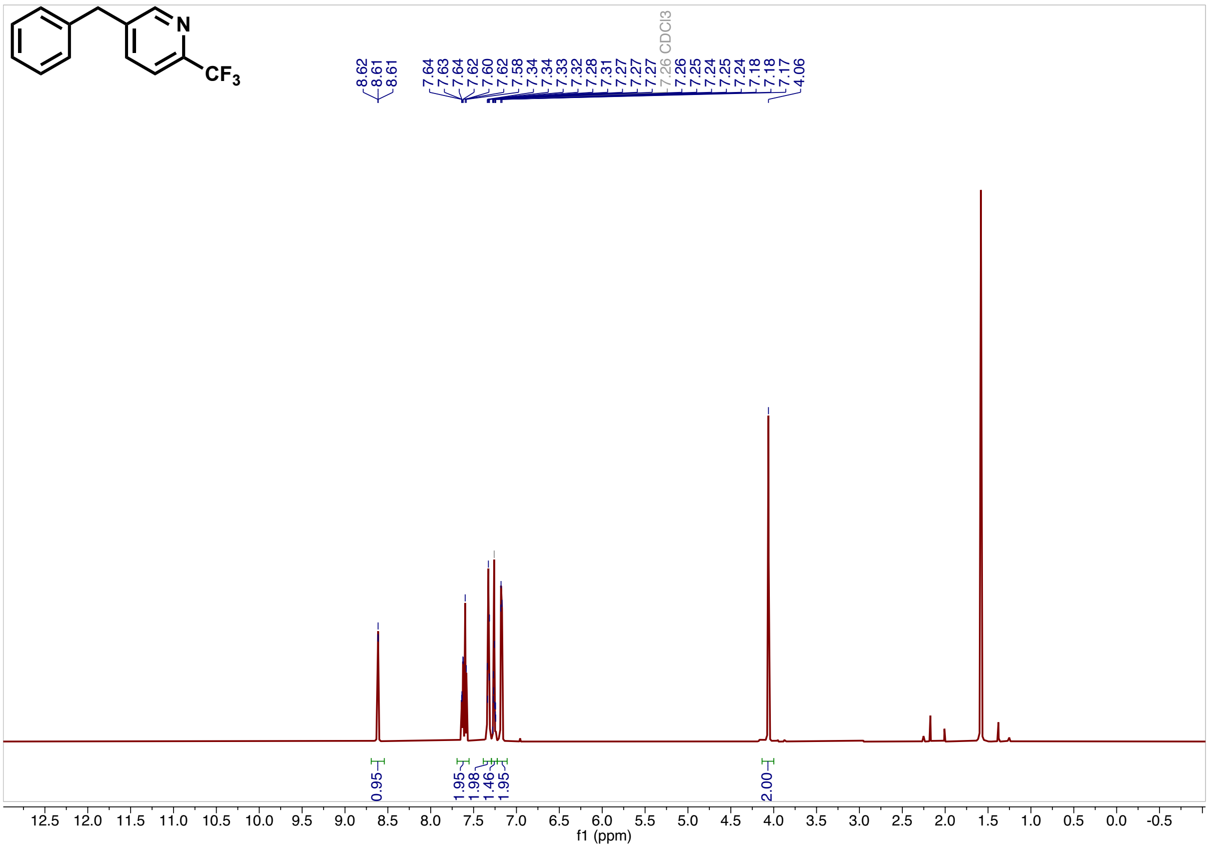

^{13}C NMR of Compound 35 (151 MHz, $CDCl_3$):

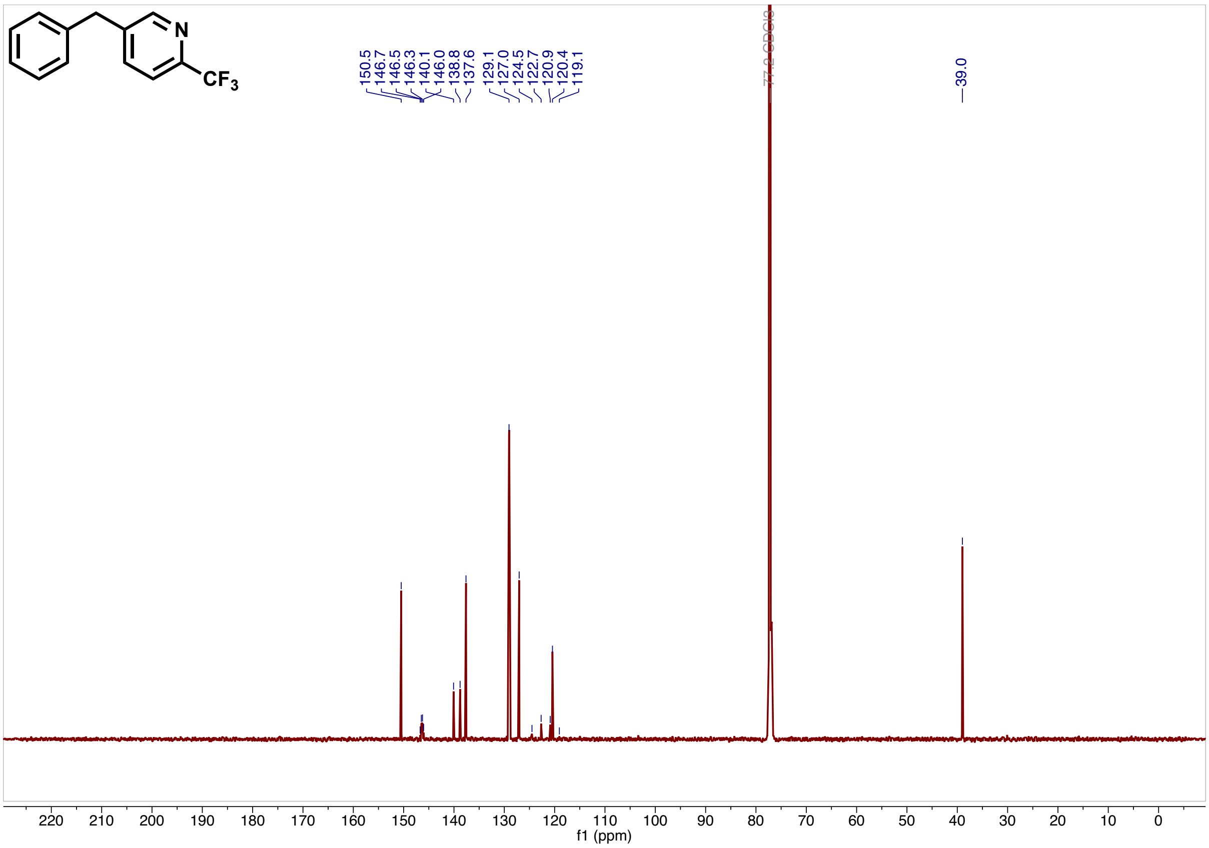

^{19}F NMR of Compound 35 (376 MHz, CDCl₃):

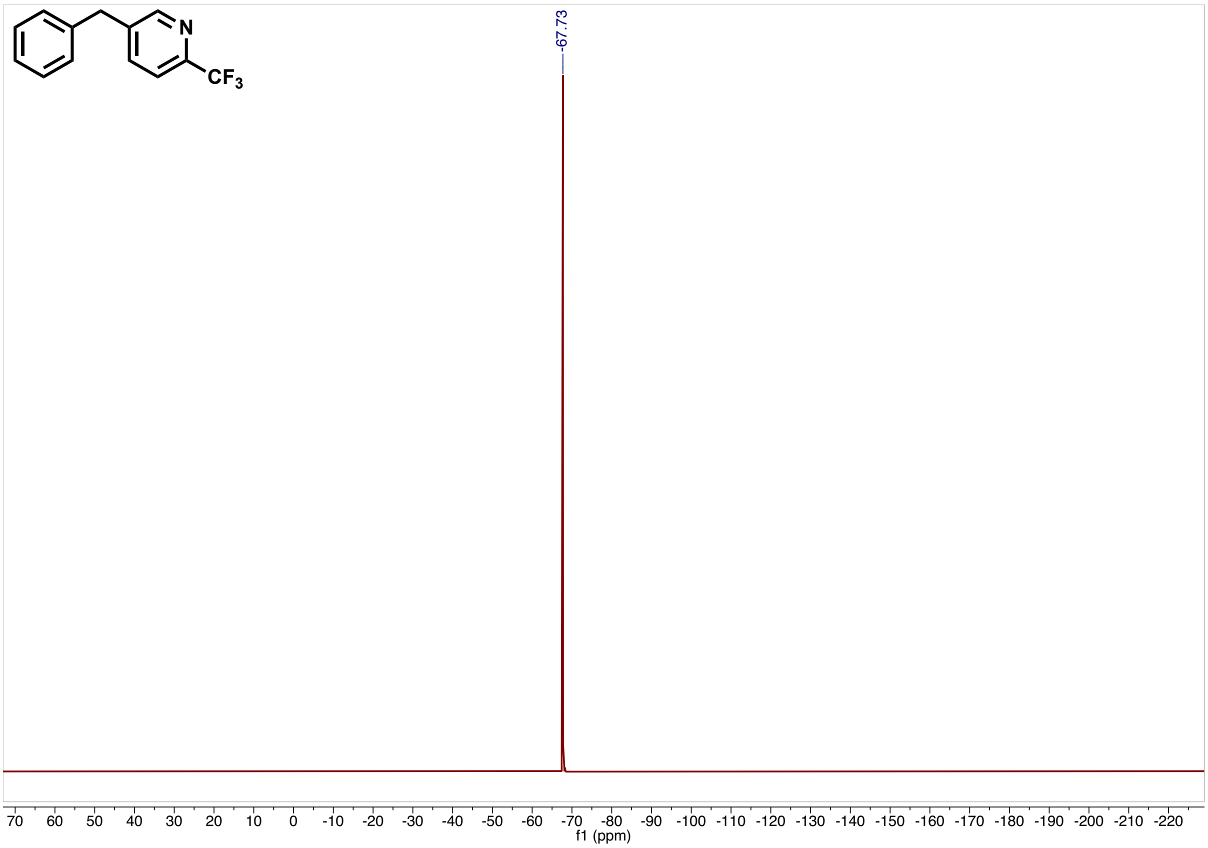

^{1}H NMR of Compound 36 (600 MHz, CDCl₃):

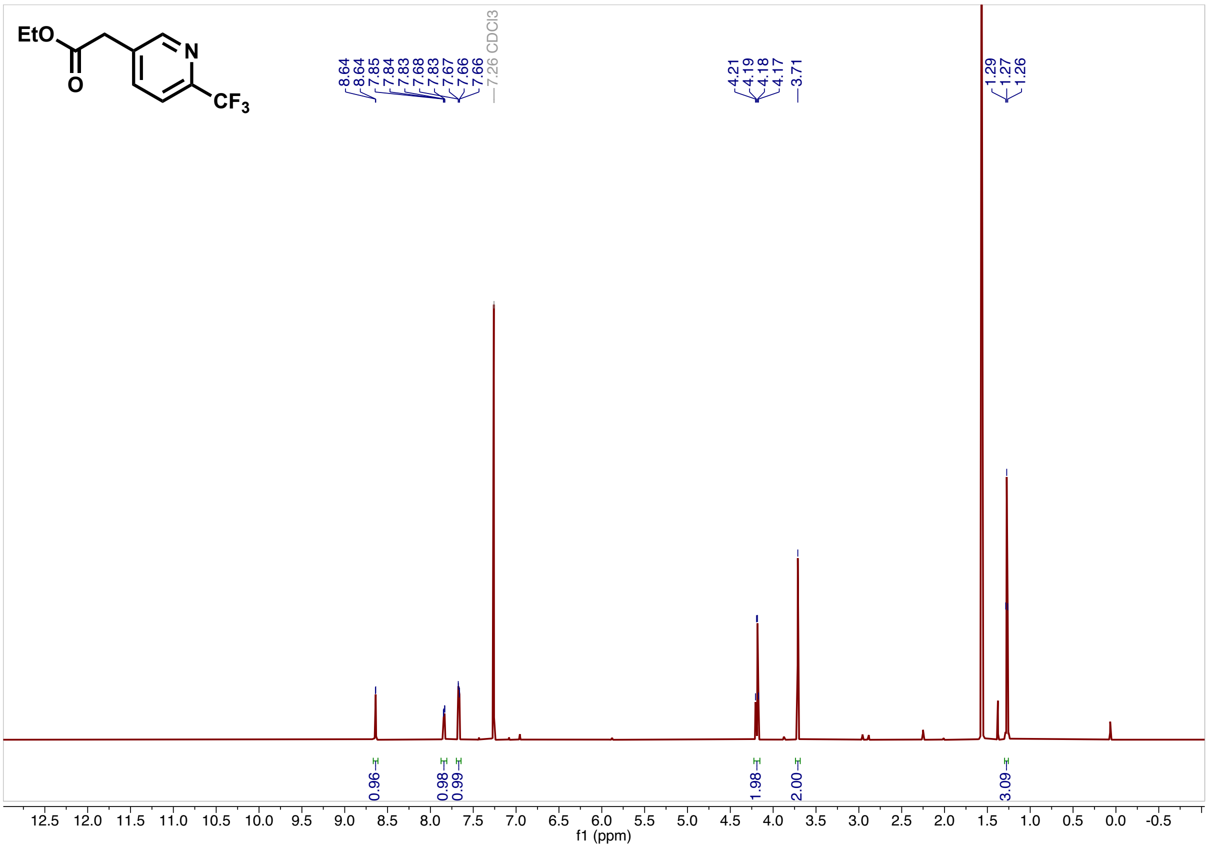

^{13}C NMR of Compound 36 (151 MHz, $CDCl_3$):

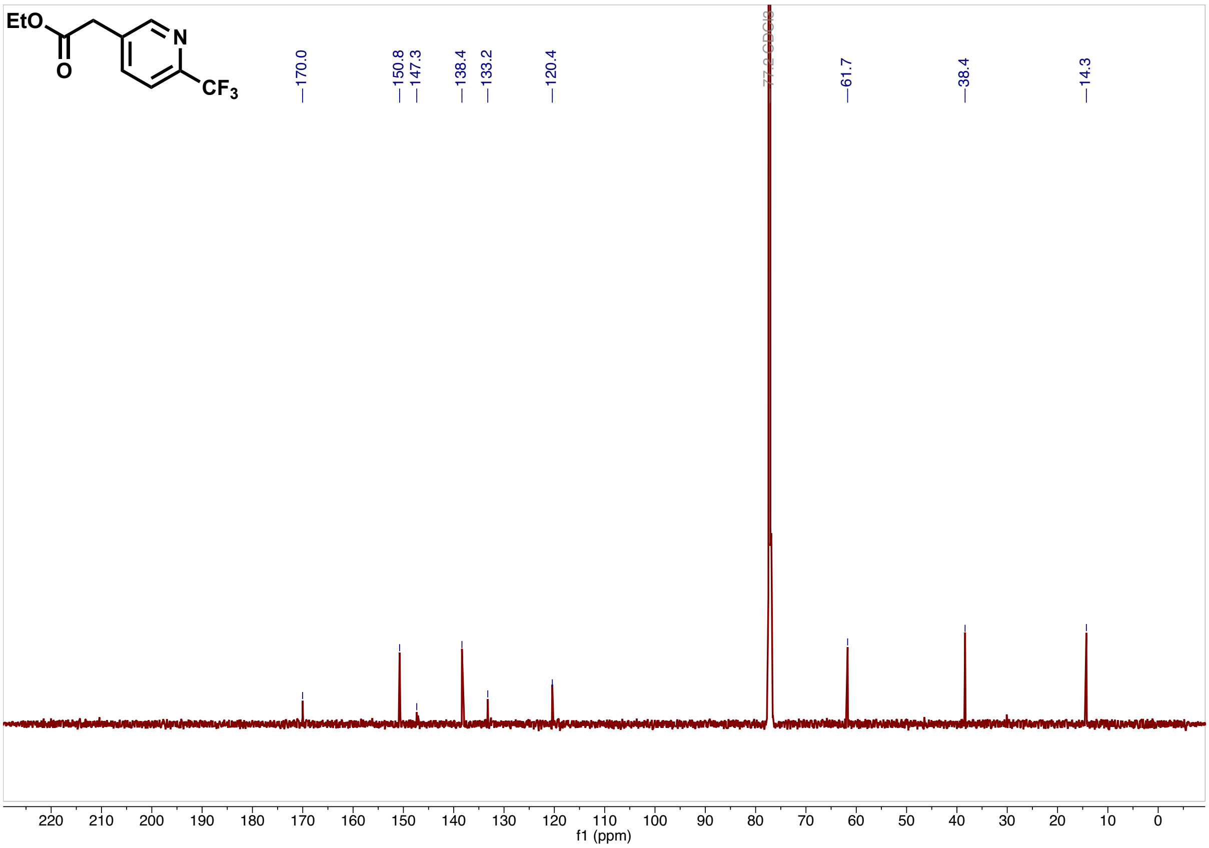

^{19}F NMR of Compound 36 (376 MHz, $CDCl_3$):

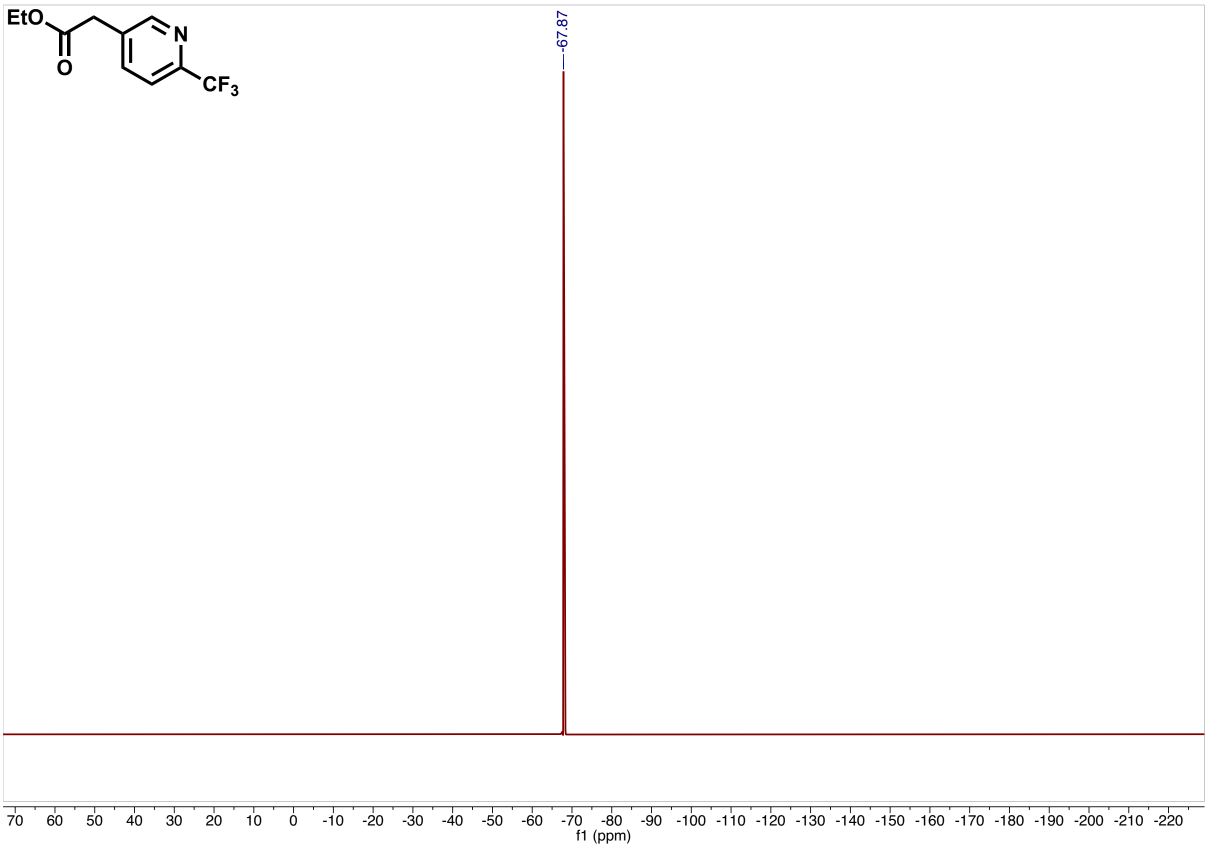

Supplement: Supplementary file 1 — Supporting File 1: anie71405‐sup‐0001‐SuppMat.pdf. [file ANIE-65-e9252206-s001.pdf]
